# Supplementary material for: Scalable and multiplexed recorders of gene regulation dynamics across weeks
Source: Nature. 2026 Jan 26;652(8111):1038–48. doi: 10.1038/s41586-026-10156-9 (PMC13102694; doi:10.1038/s41586-026-10156-9)
Supplement: Supplementary file 1 — This file contains additional details on the development and discussion of the CytoTape toolkit, Supplementary Figs. 1–24, Supplementary Tables 1–9, and Supplementary References. [file 41586_2026_10156_MOESM1_ESM.pdf]

nature portfolio

https://doi.org/10.1038/s41586-026-10156-9

Supplementary information

Scalable and multiplexed recorders of gene regulation dynamics across weeks

In the format provided by the authors and unedited

Supplementary information

Scalable and multiplexed recorders of gene regulation dynamics across weeks

Lirong Zheng, Dongqing Shi, Yixiao Yan, Bingxin Zhou, Jormay Lim, Yongjie Hou, Bobae An, Jason K. Adhinarta, Michael Lin, BumJin Ko, William C. Joesten, Mehul Gautam, Elie D.M. Huez, Eung Chang Kim, Emily G. Klyder, Boxuan Chang, Sethuramasundaram Pitchiaya, Michael T. Roberts, Denise J. Cai, Edward S. Boyden, Donglai Wei, Pietro Liò, Changyang Linghu

This file contains additional details on the development and discussion of the CytoTape toolkit, Supplementary Figs. 1–24, Supplementary Video 1, Supplementary Tables 1–11, and the Supplementary References.

Table of Contents

Design, screening, and in silico characterization for CytoTape structural monomer........5

Method for ProtSSN-based protein mutation prediction...9

Method for CPDiffusion-based protein sequence generation..10

Confidence analysis of CytoTape and XRI monomer structure predicted by AlphaFold3 11

L349K reduces fiber width in CytoTape 11

Characterization of the CytoTape assembly in cultured neurons, HEK, and HeLa cells 12

CytoTape preserves HEK cell morphology 15

Recovery of continuous time axis from timestamps along CytoTape.............................15

Development of CytoTape-based transcriptional recorders to measure gene regulation dynamics 17

Multiplexed, multi-week, multi-event recording of activity-dependent promoter-driven expression histories via CytoTape 19

CytoTape provides new insights into temporal principles of gene regulation dynamics 21

Spatiotemporally resolved, multi-brain-region, single-cell continuous recording in vivo 24

CytoTape shows superior recording performance compared to live GFP imaging. Long-term recording without continuous imaging: Spatial scalability recording across cell population and in vivo...27

Discussion, limitation, and future direction 28

Supplementary Fig. S1 Quantification of protein fiber width 32

Supplementary Fig. S2 Schematic of structural monomer binding kinetics in XRI and CytoTape during weeks-long recording (after 7 days) 33

Supplementary Fig. S3 Protein assembly design screening and characterization in cultured neurons..35

Supplementary Fig. S4 Statistical analysis of protein assembly width for B1 and C4 (left panel) and C6 and CytoTape (right panel) 36

Supplementary Fig. S5 Confidence analysis of AlphaFold3-predicted models for XRI and CytoTape monomers...37

Supplementary Fig. S6 Analysis of iPAK4 and CytoTape fiber formation in cultured neurons, HEK, and HeLa cells 39

Supplementary Fig. S7 CytoTape does not deform cell membranes or alter cell morphology....................40
Supplementary Fig. S8 Schematic of signal monomer binding kinetics of XRI and CytoTape during weeks-long recording (after 7 days) 41
Supplementary Fig. S9 iPAK4 deforms cell membranes and alters cell morphology, whereas CytoTape does not 42
Supplementary Fig. S10 Scalable temporal labeling readout of CytoTape in HEK cells43
Supplementary Fig. S11 CytoTape encodes temporal information in HeLa cells, HEK cells, and cultured neurons 45
Supplementary Fig. S12 XRI loses temporal information in HEK for 3 days and cultured neurons for 15 days....................46
Supplementary Fig. S13 Time courses of Arc-promoter-driven expression and CREB activity in cultured neurons measured by timelapse imaging of GFP reporter 48
Supplementary Fig. S14 Control experiments and cross-validation of transcriptional recorders for tracking gene regulation dynamics with CytoTape 50
Supplementary Fig. S15 CytoTape enables waveform recording of Fos-, Egr1-, and Arc-promoter activities in cultured mouse hippocampal glial cells under chemical stimulation 51
Supplementary Fig. S16 CytoTape enables recording of Fos- and Hspa1a-promoter activities in HEK cells 54
Supplementary Fig. S17 CytoTape enables recording of Fos-, Arc-, and Egr1-promoter activities and NPAS4 activity in cultured neurons 56
Supplementary Fig. S18 Temporal analysis of multiplexed recording of Arc- and Egr1-promoter-driven expression histories with CytoTape in cultured neurons....................57
Supplementary Fig. S19 Comparison of CREB activity following forskolin stimulation recorded by live GFP imaging and CytoTape in HEK cells 59
Supplementary Fig. S20 Live-cell GFP reporter imaging exhibits baseline drift over time unrelated to the biological activity of interest....................61
Supplementary Fig. S21 CytoTape captures two sequential stimulations of CREB activity undetectable by time-lapse GFP imaging under low dose forskolin treatments in HEK cells....................62
Supplementary Fig. S22 Confocal images of JF_{585} and JF_{635} on CytoTapes in HEK cells before and after photobleaching....................63

Supplementary Fig. S23 No optical crosstalk was observed between the Alexa Fluor 546 dye in the 561 nm channel and the Alexa Fluor 594 dye in the 594 nm channel under the imaging conditions used in this work 64

Supplementary Fig. S24 Comparison of CytoTape and XRI systems 65

Supplementary Video S1 Tissue-wide computational segmentation of CytoTape-vivo fibers and cell morphology for in vivo applications 66

Supplementary Table S1 Sequences of protein motifs used in this study 67

Supplementary Table S2 Constructs of signal monomer used in cultured neurons in this study 71

Supplementary Table S3 Constructs of signal monomer used in HEK293T in this study 72

Supplementary Table S4 Constructs of signal monomer used in HeLa in this study 73

Supplementary Table S5 Constructs of timestamp monomer used in cell culture in this study 74

Supplementary Table S6 Constructs used in the mouse brain in vivo in this study 75

Supplementary Table S7 Constructs of structural monomer tested in the mouse brain in vivo in this study 76

Supplementary Table S8 Constructs of structural monomer designs tested in this study 77

Supplementary Table S9 Comparison of XRI, iPAK4, and CytoTape in multiple cell types in cell culture and in vivo 79

Supplementary Table S10 List of reagents and resources used in this study 82

Supplementary Table S11 Details of statistical analysis 83

Supplementary references 84

Design, screening, and in silico characterization for CytoTape structural monomer

We first set out to design an intracellular linear protein assembly that enables multiplexed, weeks-long recording (**Fig. 1a**). We reasoned that recording more kinds of information for longer durations would demand an increased information storage capacity of the linear protein assembly. Because information is encoded and stored into molecular tags along the protein assembly in protein tape recording systems[1,2], the storage capacity is in principle proportional to the product between the number of the tags along the elongation (longitudinal) axis of the assembly (but not along the lateral axis orthogonal to the elongation axis) and logarithm base 2 of the number of possible tag variants at each tag location (*i.e.*, bit depth of the tag). Increasing the maximum length of the assembly permitted in live cells would enhance the former (for a given line density of tags defined by the line density of tag-bearing monomers of the protein assembly), while increasing the number of tag variations would enhance the latter, and we proceeded with these two directions.

We first worked towards increasing the maximum permitted length of the protein assembly in live cells. We reasoned that if the assembly is rigid, once its length reaches the size of the cell, further elongation of the assembly will either be disrupted by the intracellular spatial constraints or distort the cell membrane that has a risk of causing unwanted physiological changes of the cell being recorded[3,4]. If the assembly is flexible and could curl up in a thread-like manner in cells, it may reach lengths much longer than the cell size. In addition, we speculated that minimal lateral growth of the assembly orthogonal to the elongation axis is critical to support time-resolved recording, since in our previous work we observed that the temporal order of tag-bearing monomers can no longer be retrieved when there is significant lateral growth of the assembly over time[1]. We decided to perform protein engineering based on the XRI design[1] because, compared to the iPAK4 design[2,5-7], XRI showed slight bending in cells suggesting it is not completely rigid, XRI has no detectable sequence homology to eukaryotic genes, and XRI has a symmetrical bidirectional growth that can be conveniently used for cross-validation of recorded information from live cells. In addition, the XRI design utilizes the 1POK(E239Y) protein subunit engineered from the *E. coli* isoaspartyl dipeptidase IadA to achieve supramolecular self-assembly[8]. This assembly has been experimentally identified as a unique “agglomerate-type” arising from specific interactions among well-folded subunits, in contrast to the “aggregate-type” resulting from non-specific interactions among misfolded subunits, and has been reported to be inert in live cells without altering cellular physiology via electrophysiology, transcriptomics, proteomics, and immunohistochemistry analysis *in vitro* and *in vivo*[1,9]. We hypothesized that if we could reduce the thickness of the XRI assembly, we would soften its mechanical rigidity and increase its flexibility. We also speculated that the thickness of the protein fiber is influenced by electrostatic interactions among amino acids (AAs) on the lateral binding surfaces and the size of protein monomer, and thus minimizing lateral binding kinetics of protein monomer may suppress lateral growth of protein assembly. This, in turn,

would result in thinner assemblies that are more flexible and better suited for long-term growth and recording in live cells.

The molecular architecture of the XRI protein monomer[1] consists of three distinct domains: the self-assembling scaffold domain 1POK(E239Y)[8], the insulator domain derived from *E. coli* maltose-binding protein (MBP), and an unstructured glycine-rich linker with an epitope tag between the two domains[1]. The scaffold domain facilitates protein self-assembly, the linker connects the scaffold to the insulator, and the insulator domain reduces lateral growth of the protein assembly[1]. We first modified each of the domains individually to gain insights into how changes in each domain alter the assembly morphology in live mammalian cells, expecting that later we could combine these changes and insights to build a new protein monomer as a whole.

The overall design and screening workflow proceeded as follows (**Fig. 1b**). To begin, multiple computational and rational strategies were employed to generate and optimize protein monomers, covering the design space widely. These candidates were fused to the hemagglutinin (HA) epitope tag and then expressed in primary cultures of mouse hippocampal neurons via calcium phosphate transfection and evaluated across multiple timescales (7, 14, and 18 days after transfection) (see **Table S1** for amino acid sequences of the motifs and **Table S8** for all tested constructs). Following immunofluorescence staining of the HA tag, confocal microscopy was used for imaging, and statistical analyses were performed on the measured widths of resulting protein assemblies (**Fig. S1**). To benchmark our new designs, we measured the protein assembly width of the XRI design at 7, 14, and 18 days. The XRI assembly showed a fiber-like morphology that progressively increased in thickness over 18 days (**Fig. 1c**, highlighted in red; the schematic lateral binding of the structural monomer is shown in **Fig. S2**), suggesting continuous lateral incorporation of protein monomers.

We independently optimized the scaffold (1POK(E293Y)), the linker, and the insulator (MBP) domains by using multiple design methods described below (**Fig. 1b**). For scaffold domain, we employed three distinct design strategies in parallel: (1) applying a sequence generation model, *CPDiffusion*[10], to design novel scaffold AA sequences (we set the target sequence identity between generated sequences and the original scaffold sequence to be below 80% to favor novel designs and to broadly sample the sequences space) (referred to as the A1 family designs), (2) using a self-supervised protein sequence and structure representation model, *ProtSSN*[11], to predict beneficial single-site mutations (referred to as the A2 family designs), and (3) exploring alternative naturally existing self-assembling proteins[12-16] as potential replacements for the original scaffold domain (referred to as the A3 family designs). For the linker domain, we first relocated the HA epitope tag to the C-terminus (whereas in the XRI design, the HA epitope tag is positioned next to the linker domain between 1POK(E239Y) and MBP) and then optimized the linker by varying its length (referred to as the B family designs). For the insulator domain, we conducted homology-based

sequence searches (BLAST)[17] to identify MBP homologs with different radius of gyration (R_g) and also performed truncation and duplication of the original MBP (referred to as the C family designs). ***CPDiffusion* for protein sequence generation:** In our initial attempt to apply *CPDiffusion* to the design of protein fibers, five variants (A1-1 to A1-5) were generated that incorporated 80 to 120 amino acid substitutions (70–80% sequence identity to the original 1POK(E293Y) scaffold). These designs formed fiber-like assemblies in cultured neurons after 7 days, with thickness comparable to that of XRI (Mann–Whitney U (MWU) tests with Bonferroni correction, $P = 0.855, 0.585, 1.000, 0.952, 0.585$ for A1-1 to A1-5, respectively; significance threshold = 0.01; **Fig. 1c**, **Fig. S3**). However, the majority of observed structures exhibited low aspect ratios (< 3) and limited spatial continuity (*i.e.*, they did not form long and continuous fibers), which rendered them unsuitable for our protein tape recording application. ***ProtSSN* for single site mutation**: We next deployed *ProtSSN* to score and rank single-site saturation mutations of the 1POK(E293Y) scaffold and selected the top 2 mutations, L349K and E149I, for further experimental evaluation. We found that the L349K mutant (design A2-2) resulted in thinner protein fibers compared to the XRI fibers at 7 days of expression (**Fig. 1c**) (MWU test, $P = 0.020$). Lateral growth for this design was still observed between day 7 and day 14, but not between day 14 and day 18, indicating an improved suppression of long-term lateral growth compared to the XRI design. In contrast, the E149I mutant (design A2-1) did not lead to noticeable changes in fiber thickness compared to the XRI design (**Fig. 1c**) (MWU test, $P = 0.751$). Based on these observations, we retained the L349K mutation for subsequent design iterations. **Natural scaffold**: In nature, many proteins can form thin fibers, such as the *B. subtilis* cytoskeletal protein $MreB_{BS}$[16], *E. coli* cytoskeletal protein $MreB_{EC}$[14,15], and cytochrome-based nanowires from prokaryotes[12,13]. To explore whether these proteins have the potential to form thin and well-structured fibers in live mammalian cells, we replaced 1POK(E239Y) with these proteins in the XRI design (designs A3-1 to A3-5) and then tested them in cultured neurons. We found these designs failed to form well-structured fibers and instead produced irregular, intertwined assemblies or puncta (**Fig. 1c**, **Fig. S3**). We therefore decided not to further pursue these designs since their assembly morphologies are unlikely to support spatially resolved signal encoding and subsequent readout. **Linker optimization**: We next optimized the linker domain (**Fig. 1c**, **Fig. S3**). We hypothesized that the 36-AA long linker domain between 1POK(E239Y) and MBP may allow large conformational fluctuations in the protein monomer (this is supported by the molecular dynamics (MD) simulations described below) and some of the permitted monomer conformations could potentially provide unwanted lateral binding surfaces along the fiber, resulting in lateral growth over time. Thus, we shortened the linker to 2 AAs while also moving the HA tag next to the linker to the C-terminus of the entire protein monomer (design B-1) and found that it indeed results in thinner protein fibers (MWU test, $P = 0.0150$) with reduced lateral growth over 18 days, indicating that linker

shortening is a working design element. **MBP size tuning and optimization**: We also optimized the MBP domain (designs C1 to C5) (**Fig. 1c**, **Fig. S3**). When the original 366-AA MBP domain from *E. coli* in the XRI design was replaced with a smaller, homologous 246-AA MBP from *K. pneumoniae* (*Kp*MBP) identified by BLAST (design C-1), fibers with asymmetrical thickness (one end thicker than the other end) were observed after 7 days of expression in cultured neurons (MWU test, $P = 0.879$). When replacing the original MBP with a larger, homologous 453-AA MBP from *P. stutzeri* (*Ps*MBP) identified by BLAST (design C-2) or with tandem repeats of the original 366-AA *E. coli* MBP (design C-3), only puncta but not fibers were formed. We also fused mEGFP to the XRI monomer using a self-cleavage P2A linker (design C-5), expecting the monomeric mEGFP can sparsely and stochastically coating along the fiber to block lateral monomer binding and reduce lateral growth[1], but found this design has a fiber thickness comparable to the XRI fiber (MWU test, $P = 0.699$) that is also increasing over weeks. Knowing that shortening the unstructured linker domain decreases fiber thickness and suppresses lateral growth, we took the design B-1 above with reduced linker length and further removed the first four residues from the N-terminus of MBP (termed dMBP, 362 AAs), resulting in design C-4. Those four neighboring AAs to the linker domain were less structured than the rest of the MBP domain based on its crystal structure[18]. We hypothesized that removing these residues would further suppress lateral growth, as their flexible nature may contribute to linker-like behaviors. As expected, the resulting fibers were notably thinner than those formed by XRI (MWU test, $P = 0.003$) and B-1 (MWU test, $P = 0.0348$, **Fig. S4**) and exhibited no significant lateral growth over 7, 14, and 18 days. **Design Combination**: We combined the working design elements above and found that the incorporation of the L349K mutation, complete removal of the linker, moving the epitope tag to the C-terminus of the entire protein monomer, and the use of dMBP together resulted in a design that produced fibers thinner than those of XRI and all other designs while maintaining the same thickness over weeks (MWU test, $P < 0.0001$) (**Fig. 1c**, highlighted in blue). This optimized design is referred to as CytoTape throughout the rest of the paper (**Fig. 1d**).

To investigate the structural basis for the enhanced performance of CytoTape, we computationally analyzed the CytoTape design. AlphaFold3-predicted structural alignment between the XRI monomer and the CytoTape monomer revealed substantial conformational divergence (RMSD = 21.6 Å) (**Fig. 1e**). Analysis of the 1POK octamer structure showed that the L349K mutation is located on the lateral interface of the scaffold (**Extended Data Fig. 1a**), replacing a hydrophobic AA (leucine) with a hydrophilic AA (lysine). This AA mutation may have reduced lateral fiber growth by decreasing hydrophobic surface interactions among protein monomers at the lateral surfaces[19] and enhancing hydrophilic interactions between these lateral surfaces and the surrounding aqueous microenvironment (as most of the mammalian cytoplasm is water[20]). We further used MD simulations to characterize the conformational dynamics

of the CytoTape monomer. The CytoTape monomer exhibited a narrower free energy landscape than the XRI monomer (**Extended Data Fig. 1b**). Additionally, we calculated the distance between the centroids of the 1POK(E239Y, L349K) and dMBP, and found reduced conformational fluctuation in CytoTape monomer compared to XRI monomer (**Extended Data Fig. 1c**). Both results indicate that the increased conformational stability of the CytoTape monomer may have contributed to the formation of thinner protein fibers, as limiting the protein conformational fluctuations and transitions could in principle minimize unintended binding surfaces and favor only the longitudinal binding surfaces by design, thereby suppressing lateral growth.

Method for *ProtSSN*-based protein mutation prediction

ProtSSN[11] formulates a zero-shot mutation effect prediction task to suggest favorable modifications on a template protein. It takes the sequence and structure of a template protein (1POK(E239Y) in our case) as input and uses a deep learning model to extract residue-wise representations. Based on these representations, it produces a predicted matrix $\hat{Y} \in R^{l \times 33}$ for the template protein with each of l residues being one of 33 tokens, including 20 regular residue types and other special tokens defined for training purposes. This matrix can be used to compute mutation fitness scores by comparing the differences between mutants and the template protein at the residue level, thus identifying top-ranked candidate mutants. In the following sections, we describe the model architecture and scoring procedure in detail.

The core of *ProtSSN* consists of a pre-trained BERT-style masked protein language model (PLM) that captures long-range residue dependencies along the sequence, followed by a learnable equivariant graph neural network (EGNN)[21] that enhances local spatial interactions of residues. For an arbitrary input protein, we first define it as an undirected graph $G = (V, E, W_V, W_E, X_V)$ using a *k*-nearest neighbor (kNN) algorithm. Each node $v \in V$ corresponds to a residue. For each central node, undirected edges $e \in E$ are added to up to *k* nearest neighboring residues within a 30 Å radius in Euclidean space. Node features W_V are 1024-dimensional hidden embeddings extracted from the frozen ESM2-t33 model[22] (checkpoint available at https://github.com/facebookresearch/esm). Following graph denoising neural networks[23], edge features $W_E \in R^{|E| \times 93}$ capture pairwise relationships between connected residues based on inter-atomic distances, local backbone directions (N–C vectors), and relative sequence positions. Additionally, to maintain roto-translation equivariance and permutation invariance for node attributes during geometric message passing, X_V stores the 3D coordinates of residues in Euclidean space.

The parameters of the EGNN layers are learned through a self-supervised denoising task. For each residue in the input sequence, a random perturbation is applied with probability p using a replacement matrix $\Theta(\cdot)$, defined by empirical observations. This perturbation updates each residue v to $\hat{v}$ The frozen protein language model then

encodes the perturbed residue sequence and generates the node features $W_V = PLM_{frozen}(\hat{V})$, where $\hat{V} = \{\hat{v_1}, \ldots, \hat{v_l}\}$ is the perturbed sequence. The EGNN layers further process the representation, enhancing local environmental information, and output $W_V^L = EGNN(G)$. This is subsequently passed to the readout layers $\phi(\cdot)$ to provide the recovered sequence $\hat{Y} = \phi(W_V^L) \in R^{n\times 33}$. The training objective is to minimize $Loss(Y, \hat{Y})$, *i.e.*, the difference between the denoised and the ground-truth residue tokens.

Once the model is properly trained, it can be used to infer mutation effect scores for mutants by comparing with the template residue types V. In this case, the template sequence and structure are fed into the frozen PLM and trained EGNN layers (without additional perturbation, which is different from the training steps) to output $\hat{Y}$. Suppose the mutant of interest has mutated sites τ $(|\tau| \geq 1)$. Its fitness score is computed as $F_x = \sum_{\{t\in\tau\}} \quad log\, log\ p(\hat{y_t})\ -log\, log\, p(v_t)$, where y_t and v_t represent the mutated and wild-type residues at the tth position, respectively. While the absolute value of these fitness scores does not have a direct physical meaning, when multiple mutants are under consideration, their corresponding fitness scores are effective references for ranking the preference of these mutations. In the case of recommending single-site mutations for 1POK(E239Y) in this study, we employed *ProtSSN* to score single-site saturation mutations and selected the top 2 mutations for further investigation and validation through wet lab experiments.

Method for *CPDiffusion*-based protein sequence generation

CPDiffusion[10] is a discrete diffusion probabilistic model designed for the protein inverse folding task, *i.e.*, recovering the residue sequences $X^{aa} = \{x_1^{aa}, \ldots, x_i^{aa}, \ldots, x_l^{aa}\}$ conditioned on a given protein backbone $X^{pos} = \{x_1^{pos}, \ldots, x_i^{pos}, \ldots, x_l^{pos}\}$. To meet specific application needs and improve generation quality, the model allows the incorporation of additional conditions during the generation process, such as fixing conserved residue positions, providing reference secondary structure sequences, or including homologous sequences in the training set. In addition, the generated sequences are further refined through a series of structure-based filtering strategies to enhance the success rate of novel protein designs.

To achieve this generative objective, *CPDiffusion* establishes a progressive diffusion process over residue types and trains a denoising network to gradually recover the input sequence distribution $p(X^{aa}|X^{pos})$. The forward diffusion process adds noise step-by-step to one-hot encoded residue types based on a transition probability matrix (*e.g.*, the BLOSUM62 matrix) until time T, when $q(X_T^{aa})$ asymptotically converges to a uniform distribution and becomes independent of the initial state X^{aa}. In the denoising phase, a multi-layer EGNN is trained to predict X^{aa} from the noisy sequence X_t at time t, using the input protein backbone (represented as a *k*NN undirected graph) and additional

conditions (defined in the node attributes, such as secondary structure information). During training, the network parameters θ in $EGNN_\theta(\cdot)$ are learned from a joint training set consisting of protein crystal structures in CATH 4.2[24] and homologous sequences of 1POK (*i.e.*, *E. coli* IadA) along with their structures predicted by AlphaFold2[25], with the objective of minimizing the cross-entropy loss between the predicted sequences $\hat{p}(X^{aa}) = EGNN_\theta(X_t, E, t)$ and the ground-truth sequences X for each input protein. Optionally, a mask matrix M can be defined to fix conservative sites. In this case, the predicted sequences by EGNN requires an additional step of adjustment $\hat{p}(X_t) = M \odot p(X^{aa}) + (1 - M) \odot \hat{p}(X^{aa}|X_t)$. We fixed tyrosine (Y) at the 293 position of the amino acid sequence during the generation process to keep 293Y in all the generated novel sequences, because E293Y has been reported to be critical for the linear assembly of 1POK(E293Y)[8]. During inference, for a given target protein backbone (*e.g.*, the crystal structure of 1POK, *i.e.*, *E. coli* IadA), AA sequences X_t are sampled iteratively starting from a uniform sampling X_T at time T until time $t = 1$ when reaching $p_\theta(X^{aa}|X_1)$. Multiple sequences can then be generated by sampling from this distribution.

Following the above procedure, we used *CPDiffusion* to generate 100 sequences and predicted their structures using AlphaFold2. We then evaluated the predicted structures by comparing them to the original 1POK/IadA crystal structure in terms of RMSE, TM-score, and the residue-wise pLDDT trends of the generated sequences versus the wild-type template sequence predicted by AlphaFold2. Based on these criteria, we selected the optimal-performed 5 sequences for further experimental validation.

Confidence analysis of CytoTape and XRI monomer structure predicted by AlphaFold3

We used AF3 to analyze the PAE, iPTM, and pLDDT values of the CytoTape and XRI monomers. As shown in **Fig. S5**, both 1POK and MBP regions exhibit high-confidence predictions, which can be attributed to their presence in the AlphaFold training set as experimentally determined crystal structures. In the CytoTape monomer, the interaction region between 1POK and MBP also shows high-confidence predictions. This region corresponds to the C-terminus of 1POK and the N-terminus of MBP, both of which are resolved by X-ray crystallography. By contrast, the corresponding interaction region in the XRI monomer shows low-confidence predictions, due to the presence of flexible elements such as linker25 (25 AAs), the HA epitope tag (7 AAs), and linker3 (3 AAs). These regions form unstructured coils (resolved by X-ray crystallography) and lack defined secondary structure (α-helices or β-sheets), contributing to the reduced prediction confidence.

L349K reduces fiber width in CytoTape

To demonstrate that the L349K mutation reduces fiber thickness, we compared A2-2 with XRI and C-6 with CytoTape. In each pair, the only difference is the substitution of

leucine with lysine at position 349. As shown in **Fig. 1c** and **Fig. S4**, constructs containing the L349K mutation form significantly thinner fibers. These results indicate that L349K plays an important role in reducing the lateral thickness of protein assemblies.

Characterization of the CytoTape assembly in cultured neurons, HEK, and HeLa cells

We characterized the intracellular structure and kinetics of CytoTape in primary cultures of mouse hippocampal neurons, HEK, and HeLa cells, while comparing them to those of XRI and iPAK4. Cultured hippocampal neurons serve as a popular *in vitro* system for studying activity-dependent gene regulation dynamics and associated signaling activities and neuroplasticity outcomes[26-29]. HEK293T cells (referred to as "HEK" throughout the paper), derived from the human embryonic kidney, are commonly used to model transcriptional regulation in proliferative epithelial-like cells, among other usages[30,31]. HeLa cells, originating from human cervical cancer, provide a model of dysregulated gene expression and are frequently used to study cell cycle control, stress responses, and oncogenic pathways[32,33]. Here, we used a constitutive, ubiquitous promoter, the human ubiquitin C (*UbC*) promoter, to drive the continuous expression of the CytoTape monomer (fused to the HA tag for visualization via immunofluorescence imaging; the CytoTape monomer, with or without the HA tag, driven by the *UbC* promoter is referred to as the "structural monomer" throughout the paper) and produce growing protein assembly structures in mammalian cells. In cultured mouse hippocampal neurons, we observed differences in morphology among XRI, iPAK4, and CytoTape fibers after days- and weeks-long expressions. iPAK4 fibers induced noticeable distortions in cell membrane morphology as early as 3 days after calcium phosphate transfection (**Fig. S6a**), suggesting their limited capability to support multi-week intracellular recording. This may be attributed to previous observations that iPAK4 forms rigid crystalline fibers[2,6,7]. XRI fibers were capable of bending when being longer than the size of the soma and did not induce noticeable distortions in cell morphology, but exhibited progressive thickening after 7 days, reflecting sustained lateral growth (**Fig. 1f**, top row). In contrast, CytoTape fibers remain thin even after 18 days and exhibit substantial, thread-like bending without altering cell morphology, demonstrating its large potential for long-term recording (**Fig. 1f**, bottom row; **Fig. S7**). To characterize protein assembly kinetics in cultured neurons, we quantified the widths and lengths of XRI and CytoTape fibers at multiple time points up to 18 days (**Fig. 1g**). Both XRI and CytoTape underwent an initial phase of lateral thickening within the first 2 days, and XRI continued to grow laterally beyond this period. In contrast, CytoTape entered a plateau phase, maintaining a stable width thereafter (**Fig. 1g**, left panel), indicating a self-limiting lateral growth process. Additionally, CytoTape elongated more rapidly than XRI under the same plasmid dosage in transfection, resulting in longer assemblies between

day 4 to day 18 (**Fig. 1g**, right panel). This observation suggests that limiting lateral growth in CytoTape facilitates more efficient incorporation of protein monomers at fiber termini. We also characterized the flexibility of protein fibers by their maximum curvatures (**Fig. 1h**), indicating the thinner fibers of CytoTape are more flexible than the thicker ones of XRI, consistent with our initial design rationale. Additionally, we found over 80% of the CytoTape assemblies were localized in the neuronal soma, while the remaining ones were either partially extended to or fully located in the neurites (**Fig. S6f**). In the soma region, more than 95% of neurons (**Fig. S6g**) contained fibers rather than puncta.

To test whether CytoTape maintains its ability to capture and store physiological processes over multi-week timescales, we co-expressed a CytoTape monomer with a V5 epitope tag under the *Egr1* promoter[34] together with the CytoTape structural monomer in cultured neurons and 13 days after transfection and stimulated the neurons via KCl-induced depolarization (55 mM KCl, 1 h), a condition known to activate EGR1 expression[74] (the CytoTape monomer driven by an activity-dependent promoter is referred to as the "signal monomer" throughout the paper). For comparison, we performed the same experiment using XRI under identical conditions (**Extended Data Fig. 1d**, top row). We fixed the neurons 15 days after transfection and performed immunofluorescence imaging, and found that under this condition XRI did not report the expected V5 signal increase along the fiber. This outcome may result from significant lateral growth of fibers over time, which could permit V5-tagged signal monomers to be incorporated along the lateral surfaces of fibers and thus the temporal order of monomers along the longitudinal axis is no longer preserved (**Extended Data Fig. 1d**, second row; the schematic lateral binding of the signal monomer is shown in **Fig. S8**). In contrast, CytoTape maintained a thin, flexible architecture and reported symmetrical V5 signal peaks along the fiber. Quantitative analysis confirmed that CytoTape was capable of recording physiological signals two weeks post-transfection in cultured neurons (**Extended Data Fig. 1d**, right panel). We also tested whether CytoTape could resolve hours-scale temporal features of cellular activities and capture multiple cellular events over time, a desired capability for studying many cell biology processes. We performed two discrete KCl stimulations (55 mM KCl, 1 h each) separated by 12 h to the same neurons starting on day 5. We found CytoTape captured both events as distinct peaks along the fiber (**Extended Data Fig. 1e**, left panel), indicating its ability to resolve hours-scale sequential transcriptional events over time. This result was further supported by statistical analysis (**Extended Data Fig. 1e**, right panel).

Next, we evaluated the CytoTape performance in HEK and HeLa cells, comparing it to XRI and iPAK4. In HEK cells, XRI formed thick fibers and puncta (**Extended Data Fig. 1f**) while iPAK4 fibers distorted cell morphology (**Fig. S6a**; **Fig. S9a**), in agreement with previous reports[1,2]. In contrast, CytoTape produced thin, flexible, and well-structured fibers without altering cellular morphology (**Extended Data Fig. 1f**, **Fig. S7**

and **S9b**). Similarly, in HeLa cells, iPAK4 slightly changed cell morphology (**Fig. S6a**, right panel) and XRI assemblies appeared intertwined, whereas CytoTape consistently formed uniform linear structures (**Extended Data Fig. 1f**). We further tested whether CytoTape affects cell mitosis in HEK cells. The results show that CytoTape does not interfere with cell division nor split into two copies (**Fig. S6b**), which may be attributed to the thin and flexible nature of the fiber, allowing it to move and bend within the cell during division and eventually be inherited by one of the daughter cells (or by both daughter cells, if there are multiple fibers in the parent cell that may distribute into both daughter cells). Additionally, over 90% of transfected cells contained CytoTape fibers, rather than puncta, in HEK and HeLa cells (**Fig. S6h** and **i**). CytoTape's ability to maintain structural integrity and compatibility across cell types could be attributed to the low free-energy landscape of its monomer (**Extended Data Fig. 1b** and **c**), making it less affected by perturbations from the surrounding microenvironment. We also observed that the growth rate of CytoTape fiber varies across different cell types, with the monomer expression driven by the same *UbC* promoter and transfected at comparable plasmid dosages within the same order of magnitude (**Fig. 1g**, **Fig. S6d** and **e**); specifically, the growth rate was highest in HEK cells, followed by HeLa cells, and lowest in cultured neurons. We further tested whether CytoTape can capture physiological processes in HeLa cells. We expressed a V5-epitope–tagged reporter under the heat shock-responsive human *Hspa1a* promoter[35] and applied heat shock stimulation (42 ^{o}C, 1 h) in HeLa cells (**Fig. S6c**). We observed a clear increase in V5 signal towards the CytoTape termini, indicating that CytoTape can encode physiological signals in HeLa cells. Next, we examined the cellular and physiological states of HEK cells expressing CytoTape. As shown in **Extended Data Fig. 3**, the majority of HEK cells contained CytoTape fibers. We performed immunohistochemical characterization and fluorescence-based viability assays and found that CytoTape expression did not alter cellular or physiological state markers (**Extended Data Fig. 3**), including Cleaved Caspase-3 (apoptotic marker), GRP78/BiP (endoplasmic reticulum stress marker), Ki-67 (cell proliferation marker), TOMM20 (mitochondrial integrity marker), Ethidium Homodimer-1 (membrane permeability and DNA damage marker), and Calein-AM (live-cell viability indicator). We then examined the transcriptional activities and signaling of HEK cells expressing CytoTape, using markers including EGR1 (early growth response 1; IEG marker), pElk-1 (ERK-dependent transcription factor activation marker), pCREB (cAMP/PKA-dependent transcription factor activation marker), FOS (IEG marker), and pERK1/2 (ERK/MAPK pathway activation marker). **Extended Data Fig. 3** shows that CytoTape expression did not alter the transcriptional or signaling activities of HEK cells. In addition, we performed electrophysiology of cultured neurons expressing CytoTape and observed that CytoTape expression did not alter their intrinsic electrophysiological properties or calcium dynamics (**Extended Data Fig. 2**).

Collectively, these findings suggest CytoTape is a flexible and versatile intracellular recording platform across cell types, promising to achieve enhanced temporal scalability of recording durations.

CytoTape preserves HEK cell morphology

We observed that CytoTape fibers reached lengths of ~20-30 µm after 3 days of continuous expression in HEK cells (**Fig. S9b**). This exceeds the diameter of HEK cells, which is ~15-20 µm, indicating that the assemblies grew beyond the spatial confines of the cell body. Despite this, CytoTape fibers remained thin and flexible, exhibiting a thread-like morphology that curled within the cytoplasm without causing observable deformation of the plasma membrane or cellular architecture (**Fig. S9b**, right panel). By contrast, iPAK4 formed rigid crystalline fibers that distorted cell morphology (**Fig. S9a**), reinforcing our hypothesis that long, rigid assemblies are poorly tolerated in live cells (**Fig. S9a**, right panel). These observations validate our engineering rationale: flexible assemblies, such as those formed by CytoTape, can accommodate intracellular spatial constraints and achieve elongation over multiple days or weeks without perturbing cell structure. This behavior was consistent across a range of cell sizes, with CytoTape preserving cell morphology due to its flexibility, whereas iPAK4 induced shape distortion as a result of its rigidity.

Recovery of continuous time axis from timestamps along CytoTape

Protein-assembly-based recording systems convert the time dimension into the spatial dimension, and it is critical to establish the conversion relationship between the two dimensions. In XRI, a chemically inducible Cre system was used to initiate time encoding, enabling a global time calibration across cells. However, a single global time axis does not account for the variabilities in the nucleation time (*i.e.*, the time when the fiber begins to form) and the elongation rate of protein assembling across cells and cell types (**Fig. 1g**, **Fig. S6d** and **e**), and thus the precision of the space-to-time conversion is limited by these variabilities. Establishing individualized time courses at single-cell resolution instead would allow for more precise temporal interpretation of protein-assembly-based recordings, with each fiber recording and maintaining its own internal time axis. The iPAK4-based system demonstrated that the labeling of HaloTag molecules along linear protein assemblies via temporal switching of multiple HaloTag ligand dyes has effective time constants below 1 hour[2], providing sufficient temporal precision for our purpose to revolve and record long-term (multi-day and multi-week) cellular physiological activities. Therefore, we employed the HaloTag/dyes system as timestamps along the CytoTape. To implement this, we fused HaloTag to the C-terminus of the CytoTape monomer, which was expressed under the *UbC* promoter (referred to as the "timestamp monomer" throughout the paper) (**Fig. 2a**). By sequentially switching dyes of distinct colors, and using the cell fixation event as the

final timestamp (corresponding to the termini of the fiber), we introduced discrete timestamps along the fiber to calibrate its own time axis. To recover the continuous time axis along the CytoTape, we calculated the relationship between the spatial axis and the time axis by spatiotemporal interpolation of the timestamps. Although this interpolation introduces time uncertainties in fiber segments in between the timestamps, the resulting time accuracy (< 1 day) from day-scale dye-switches is sufficient for our purpose to resolve days- and weeks-long cellular events. Nevertheless, we speculate that increasing the density of timestamps along the fiber improves the precision of the reconstructed time axis, enabling more accurate temporal profiling at single-cell resolution.

We first tested HaloTag-based time encoding in HEK and HeLa cells by co-expressing the CytoTape structural and timestamp monomers. On the day of plasmid transfection into HEK cells, JF_{585} (Janelia Fluor) dye was added to the culture medium. Two days later, the medium was washed five times and then replaced with JF_{635}-containing medium (**Fig. 2b**). We observed that the dye-switching events are successfully recorded as timestamps along individual fibers across the cell population (**Fig. 2c**; large-scale readout of CytoTape timestamps across HEK cell population is shown in **Fig. S10**), resulting in a decline in JF_{585} intensity accompanied by a simultaneous rise in JF_{635} intensity upon dye switch (**Fig. 2d**). Additionally, we observed that the positions of timestamps along fibers varied among cells, confirming that single-cell–level timestamps can improve the precision of the reconstructed time axis (**Fig. 2e**, top panel). Since this case included only one dye-switching event and the fixation event, we applied a linear interpolation to approximate the elongation kinetics, resulting in a low-precision estimate of the continuous time axis (**Fig. 2e**, bottom panel). We also tested this timestamp approach in HeLa cells (**Fig. S11a**). JF_{635} dye was added on day 0, switched to JF_{585} on day 2, then switched back to JF_{635} on day 3, and cells were fixed on day 4 (**Fig. S11b**). As expected, two distinct dye-switching events were encoded along the CytoTape fibers (**Fig. S11c**), allowing us to generate a more precise time axis via interpolation (**Fig. S11d**). We further tested a two-dye switching scheme (JF_{585} and JF_{635}) for timestamps in HEK cells, which also performed well (**Fig. S11e-f**).

Next, we tested whether CytoTape recording is temporally scalable, achieving user-defined control of temporal resolution and recording duration by adjusting both the timing of dye switches and the duration of fiber elongation. To demonstrate this unique capability (**Fig. 2f** and **g**), we tested three distinct temporal configurations in cultured neurons: (1) a single dye switch at day 3 followed by fixation at day 5 (**Fig. 2h**, top row); (2) two dye switches at days 4 and 8 followed by fixation at day 9 (**Fig. 2j**, top row); and (3) two dye switches at days 5 and 10 followed by fixation at day 15 (**Fig. 2l**, top row). Single-fiber results (**Fig. 2h**, **j**, and **l**, bottom row) and statistical analysis (**Fig. 2i**, **k**, and **m**, top panel) showed one signal transition in the single-dye-switch condition and two transitions in the double-dye-switch condition. For each case, we performed

interpolation between the spatial axis along the fiber and the time axis using the dye switching and cell fixation time points, to recover the continuous time axis along the fiber (**Fig. 2i**, **k**, and **m**, bottom panel). These results validate that both temporal resolution and timescale can be flexibly defined by the user based on specific experimental needs. Finally, we quantified the percentage of CytoTape that successfully incorporated HaloTag dye timestamps. In both cultured neurons and HEK cells, over 90% of fibers showed timestamp incorporation, while in HeLa cells, the success rate exceeded 80% (**Fig. S11g**). We also tested whether XRI can preserve temporal information in neurons and HEK cells (**Fig. S12**). We found that, due to lateral binding of monomers in XRI fibers after long-term recording in neurons and the instability of fiber formation in HEK cells, XRI cannot robustly record temporal information, which is consistent with the results shown in **Extended Data Fig. 1d**.

To evaluate temporal resolution of CytoTape, we performed dye-switching experiments using HaloTag ligands with given labeling intervals (Δt = 24 h, 3 h, 2 h, and 0.5 h, **Fig. 2n-p**) as done in the iPAK4 system[40], showing CytoTape achieves at least 0.5 h temporal resolution. We also demonstrated that CytoTape can achieve sub-daily temporal resolution while maintaining stability during long-term recording (**Fig. 2q-s**). These results demonstrate that CytoTape can reliably record analog transcriptional signals with down to minutes-scale time precision, despite non-linear fiber growth.

Development of CytoTape-based transcriptional recorders to measure gene regulation dynamics

Immediate early genes (IEGs) play a critical role in cell biology, acting as rapid responders to diverse stimuli and serve as gateways in the regulation of many cellular processes, such as those associated with gene expression, cell cycles and states, and cell plasticity in both health and disease[36-40]. We tested CytoTape with four well-characterized IEG promoters of *Fos*[41], *Egr1*[34], *Arc*[42], and NPAS4[43,44], which have been used extensively to link reporter expression to these IEG activities. We also tested the phosphorylated cAMP response element-binding protein (pCREB)-responsive promoter with CytoTape[45], since the transcription factor activity of CREB has been reported to play a key role in regulating IEG expression and is involved in a broad range of cellular processes, including cell proliferation, differentiation, survival, immune responses, calcium signaling, metabolism, and stress adaptation[46-49]. We previously validated the fidelity of the XRI recording system in cultured neurons, demonstrating the protein tape recorder-based readout of *Fos*-promoter activity closely mirrored the results captured by conventional *Fos*-GFP reporter assay via live-cell timelapse imaging of GFP under identical stimulation conditions[1]. This direct comparison indicates that temporal signals from activity-dependent promoters can be reliably embedded and decoded from self-assembling protein structures in live cells. We further performed the same GFP reporter assay using the *Arc*- and CREB activity-dependent promoters under KCl and FSK

stimulations, respectively, to further validate the fidelity of downstream CytoTape recordings (**Fig. S13**). For CytoTape, we used the IEG- or pCREB-responsive promoter to drive expression of the CytoTape monomer fused to the V5 epitope tag, *i.e.*, the signal monomer, and co-expressed it with the structural monomer under the constitutive *UbC* promoter in cultured neurons, to enable post-fixation readout of the recorded promoter activities via the V5 signal intensity profile along the fiber (**Extended Data Fig. 4a**). The signal monomer plasmid was diluted to 25% of the amount of the structural monomer plasmid in transfection to ensure that the structural monomers dominate the fiber formation and elongation, thereby providing a consistent substrate for the incorporation of signal monomers over time. Neurons were stimulated on day 5 after transfection with either KCl, to induce depolarization and activate IEG activities[50], or forskolin (FSK), to raise intracellular cAMP levels and activate the CREB activity[51], and then fixed and immunostained against HA and V5 tags on day 7 (**Extended Data Fig. 4b** and **c**). As expected, in KCl or FSK stimulated neurons, we observed low pre-stimulation baseline of V5 immunofluorescence intensity at the center of the CytoTape fiber and peak(s)-like V5 intensity profiles on each of the two sides of the fiber that eventually declined towards the fiber termini (**Extended Data Fig. 4d-l**). This characteristic pattern was absent in unstimulated neurons, where V5 signals remained at a uniformly low baseline level along each fiber (**Fig. S14**). These results are in agreement with the recordings from the GFP reporter assay (**Fig. S13**). Although when averaged across cells, the mean signal appeared to be a single peak waveform, we found that individual neurons could exhibit complex waveform patterns. For example, *Arc* and pCREB signals in some neurons display two peaks following a single KCl stimulation and FSK stimulation, respectively. This result indicates that the temporal dynamics of these recorded transcriptional activities over days are highly heterogeneous across cells even under identical stimulation conditions (**Extended Data Fig. 4e-m**, fourth row, three representative traces are highlighted in magenta). This could be attributed to the fact that genetically identical neurons can exhibit markedly different gene expression dynamics in response to the same stimulus, particularly in complex regulatory pathways like those involving immediate early genes[52]. We also compared the recorded signals across multiple CytoTape assemblies within the same neuron, when there are more than one assembly per neuron (**Fig. S14n**). We found that distinct assemblies within the same cell reported highly similar signal waveforms, suggesting that CytoTape robustly records transcriptional activity in cells and that the cell-to-cell variability observed in our previous experiments may reflect biological differences in cellular transcriptional responses. To assess the sensitivity of the CytoTape recorders, we titrated a series of doses and durations of KCl and FSK to neurons expressing the structural monomer and the signal monomer under IEG- and pCREB responsive-promoters. We found stronger and longer stimulation protocols yielded higher and steeper peak(s) in the V5 signal waveforms than weaker or shorter

stimulations (**Extended Data Fig. 4n-r**), demonstrating that CytoTape is an analog recorder that captures not only the transcriptional events but also the amplitudes of these events in response to variable physiological inputs. Importantly, in the absence of stimulation, V5 signals remained flat at the baseline level over time, confirming that the observed responses were specifically induced by the applied stimuli rather than spontaneous neuronal activities. Interestingly, CytoTape also captured IEG-promoter-driven gene expression in mouse hippocampal glial cells that were co-cultured with neurons, showing its potential for studying gene regulation dynamics in glia and the coordination of gene regulation between neuronal and glial cell populations (**Fig. S15**).

Multiplexed, multi-week, multi-event recording of activity-dependent promoter-driven expression histories via CytoTape

We next evaluated whether CytoTape could simultaneously record gene regulation dynamics and timestamps by integrating activity-dependent promoters with the previously described timestamp strategy via HaloTag and dye switches. We co-expressed the structural monomer, the timestamp monomer, and the *Fos* signal monomer in HEK cells[53,54] (**Fig. S16a**). JF_{635} dye was added on day 0 and followed by a switch to JF_{585} on day 2 concurrent with FSK stimulation (10 μM, 1 h) (**Fig. S16b**). Cells were fixed on day 3. Following immunostaining, CytoTape fibers exhibited clear dye switches and *Fos*-promoter-driven CytoTape monomer expression across the cell population (**Fig. S16c**, left panel). Spatially localized V5 signal bands were observed along the fibers, indicating capturing of stimulus-induced transcriptional activity (**Fig. S16c**, right panel). To temporally resolve these signals, we reconstructed a continuous time axis by interpolating the timestamps and fixation time point, and then aligned the resulting timeline with the V5 intensity profile. Plotting the V5 signal relative to baseline as a function of reconstructed time revealed a distinct peak after day 2, consistent with the actual timing of FSK stimulation (**Fig. S16d**). Furthermore, we observed variability in the shape and amplitude of V5 signal waveforms across individual cells, suggesting heterogeneous *Fos*-promoter-driven transcriptional dynamics within the cell population. To test whether CytoTape could resolve sequential transcriptional events, we performed two FSK stimulations separated by 1.5 days in the same HEK cell culture (**Fig. S16e**). We recovered two discrete peaks in the V5 signal profile, corresponding to the timing of each stimulation (**Fig. S16g**, left panel). We further tested the heat shock-responsive *Hspa1a* promoter to evaluate whether CytoTape could record cellular stress responses in HEK cells, a process that gained popularity in cell stress and survival research[35,55,56] (**Fig. S16h**). After heat shock treatment, we observed clear V5 signal induction along fibers (**Fig. S16j**, left panel), while the untreated control group showed no significant changes in the V5 signal (**Fig. S16g** and **j**, right panel). In addition, we demonstrated CytoTape recording of the time courses of IEG-promoter-driven activities (*Fos*, *Egr1*,

Arc, and NPAS4) in response to KCl stimulation in cultured neurons with timestamps (**Fig. S17**).

To evaluate CytoTape's potential for long-term recording, we extended the recording period to 21 days. We first co-expressed the structural monomer, the timestamp monomer, and the *Fos* signal monomer in cultured neurons (**Fig. 3a**). JF_{585} dye was added on day 0, followed by JF_{635} on day 18, and JF_{503} on day 20. Cells were fixed on day 21 (**Fig. 3b**). Neurons were stimulated with 55 mM KCl for 1 h on both day 18 and day 20. After 21 days of CytoTape growth, CytoTape formed long, flexible fibers (**Fig. 3c**). We then recovered the time axis from timestamps and analyzed the V5 signal intensity along the fiber and observed two distinct peaks corresponding to the two sequential stimulation events, with the V5 signal onset occurring after each stimulation. Additionally, we observed variability in the shape and amplitude of V5 signal waveforms across individual cells under identical KCl stimulation conditions, suggesting heterogeneity in transcriptional dynamics in neurons (**Fig. 3d**). We further analyzed the amplitudes of the two peaks following the two identical KCl stimulations across neurons and found no significant difference between the two peaks (**Fig. 3e**, left panel). We also calculated the full width at half maximum (FWHM) of the V5 signal peaks (**Fig. 3e**, right panel) and found that the FWHMs were on the order of several hours and were comparable between the two sequential V5 signal peaks, indicating that CytoTape can resolve hours-scale transcriptional activity. We tested an additional stimulation condition—15 uM FSK for 1 h on both day 3 and day 5, representing closely spaced dual stimulations—during long-term (21-day) recording with CytoTape in cultured neurons (**Fig. 3f-i**). CytoTape successfully resolved both events, and the FWHM of the V5 peaks (**Fig. 3j**) showed no significant differences, correlated with the identical stimulation conditions. We also tested CytoTape with widely spaced dual stimulations—15 µM FSK for 1 h on day 3 and 1.5 h on day 15 (**Fig. 3k-n**). As expected, CytoTape successfully recorded both events, reporting distinct FWHMs of the signal peaks that correlated with the two different stimulation strengths (**Fig. 3o**). The above results demonstrate that CytoTape enables resolving multiple physiological events over three weeks.

To evaluate whether CytoTape can achieve simultaneous recording of multiple IEG-promoter-driven signals—a desired capability for studying complex processes such as development, disease progression, and neural computation[57-59]—we co-expressed the structural monomer, the timestamp monomer, the *Fos* signal monomer fused to the V5 tag, and the *Arc* signal monomer fused to the OLLAS tag in cultured neurons (**Fig. 3p**). Dye switches were performed on day 6 and day 7 as timestamps, and cells were fixed on day 8 (**Fig. 3q**), and neurons were stimulated with 10 µM FSK for 1 h on day 6. We observed that both *Fos*-promoter-driven (V5) and *Arc*-promoter-driven (OLLAS) signals increased after FSK stimulation, validating that CytoTape can simultaneously record multiple distinct gene regulation dynamics along the same fiber and within the same cell

(**Fig. 3s**). Further analysis of the signal waveforms revealed that, while the *Fos*-promoter-driven signal initiated earlier than the *Arc*-promoter-driven signal, the two signals exhibited synchronized peak timing (**Fig. 3t**). To assess the multiplexing capacity of CytoTape, we co-expressed the structural monomer, the timestamp monomer, the CREB activity signal monomer fused to the HA tag, the *Egr1* signal monomer fused to the E tag, the *Fos* signal monomer fused to the OLLAS tag, the *Arc* signal monomer fused to the V5 tag, and the NPAS4 activity signal monomer fused to the FLAG tag in HEK cells (**Fig. 3u**). Timestamps were introduced by dyes switches on days 2 and 3, a 1-h stimulation with 50 μM FSK was applied on day 3, and cells were fixed on day 5 (**Fig. 3v**). We found that CytoTape can simultaneously record both the timestamps and the responses in the five distinct gene regulation dynamics in response to FSK stimulation, all within a single protein fiber (**Fig. 3w**). Unlike other signals, we did not observe a significant change in the NPAS4 signal (**Fig. 3w** and **x**), in agreement with the literature that the NPAS4 promoter cannot be activated by FSK[60].

CytoTape provides new insights into temporal principles of gene regulation dynamics

We next applied CytoTape to investigate the temporal relationship between the CREB activity and the FOS activity—two extensively studied transcription factor activities involved in cell proliferation, signal integration, and transcriptional regulators in human cell lines such as the HEK cells[61]. While CREB is canonically recognized as an upstream activator of FOS, we explored whether they could exhibit complex dynamical features and non-linear temporal couplings beyond simple, linear correlations[62,63]. Such regulatory complexity could support history-dependent responses, nonlinear signal integration, and memory-like behaviors of gene regulatory networks[64]. To independently track the dynamics of each component, we co-expressed the structural monomer, the timestamp monomer, and two signal monomers driven by the pCREB-responsive promoter (regulated by phosphorylated CREB protein) and the *F*-RAM promoter (regulated by FOS protein activity)[43] (**Fig. 4a**), respectively, in HEK cells for 4 days (**Fig. 4b**), with FSK (50 μM, 1 h) applied on day 2.25 (6 h after day 2) to activate the cAMP–CREB pathway.

We first averaged the signals across all HEK cells (**Extended Data Fig. 5a**) and found that, on average, CREB activation precedes FOS expression, consistent with the expected upstream role of CREB in the transcriptional cascade. To further investigate the temporal relationship of the CREB–FOS transcription factor activities in single cells, we calculated their time-lagged correlations within individual cells, to quantify how changes in one signal are correlated with the changes in another. Dimension reduction of the time-lagged correlation analysis revealed two distinct clusters of signal pairs in single cells (**Fig. 4d**), defined as Type 1 and Type 2. Cells in the Type 1 cluster ("decoupled mode") displayed low or no correlation between CREB and FOS activities

at any time lag, indicating a decoupled regulatory mode (**Fig. 4e**, left panel). By contrast, cells in the Type 2 cluster ("coupled mode") showed strong positive correlations around zero-time lag, indicative of tightly coupled CREB and FOS dynamics (**Fig. 4e**, right panel). This bifurcation was further evident in the recorded waveforms: while Type 2 cells showed a canonical CREB–FOS coupling pattern with post-stimulation FOS induction (**Fig. 4g**), FOS responses in Type 1 cells appeared to be more irregular and chaotic despite a comparable CREB activation pattern (**Fig. 4f**). In addition to the differences in CREB and FOS dynamics following FSK stimulation, we hypothesized that their pre-stimulation baseline activity patterns may reflect aspects of cellular states and thus contribute to the divergent post-stimulation responses observed in these two types. Interestingly, although the level of fluctuation (quantified by line length, a popular metric in waveform analysis of neuronal electrophysiology[65]) of CREB activity in the pre-stimulation period was similar between the two types (**Fig. 4h**), the pre-stimulation fluctuation level of FOS activity was modestly higher in the decoupled group. In addition, following stimulation, decoupled cells exhibited significantly lower FOS activity levels than their coupled counterparts (**Fig. 4i**). This suggests that a history of a more active FOS activity—potentially reflecting a refractory or saturated regulatory state—may attenuate the cell's transcriptional response to CREB activation[66]. As mechanistic and causal investigations of this process are beyond the scope of this study, future research may elucidate whether a form of molecular homeostasis is involved, where prior activities or occupancy of associated molecular components dampens the responsiveness of FOS to the subsequent CREB activation—a potential regulatory mechanism that is difficult to capture without simultaneous multiplexed recording at the single-cell level. To validate the observed complex kinetics between CREB and FOS activities, we performed immunostaining for pCREB and FOS proteins, as well as fluorescence reporter assays at 1 hour and 24 hours after FSK stimulation at 1 hour and 24 hours after FSK stimulation (**Extended Data Fig. 5b-e**). The results showed that pCREB and FOS signals were heterogeneous and uncorrelated across a large fraction of cells, supporting the observations from CytoTape recording.

To investigate the molecular basis of CREB–FOS decoupling, we asked whether other signaling inputs influence whether CREB phosphorylation is transduced into FOS transcription (**Extended Data Fig. 6a** and **b**). Because the *Fos* promoter contains both the cAMP reponse element (CRE, regulated by cAMP→PKA→pCREB signaling) and the serum response element (SRE, regulated by MEK→ERK→pElk-1/SRF signaling), we hypothesized that CREB-FOS decoupling arises when ERK signaling is absent or ineffective to integrate inputs. Consistent with this hypothesis, CytoTape showed that inhibition of ERK signaling by U0126, a MEK inhibitor, abolished FOS induction under FSK stimulation, whereas activation of ERK signaling by epidermal growth factor (EGF) resulted in markedly strong FOS induction under FSK stimulation (**Extended Data Fig. 6c** and **d**). In both conditions, CREB remained robustly activated by FSK. These results

were independently validated by protein immunostaining against pCREB and FOS (**Extended Data Fig. 6e** and **f**). Compared to FSK alone, FSK+U0126 increased the portion of cells in the decoupled state while FSK+EGF did not decrease this portion (**Extended Data Fig. 6g** and **h**), indicating that ERK signaling is necessary but not sufficient for robust FOS induction and that other mechanism(s) beyond ERK signaling may contribute to CREB—FOS decoupling. These results suggest a signal- and state-dependent mechanism in IEG regulation, demonstrating the utility of CytoTape in dissecting temporal cellular signals.

Previous studies have reported both the coordinated and differential roles of distinct IEGs in mammalian neurons[43,67-70]. To investigate how IEGs are coordinately and differentially regulated across time, we applied CytoTape to simultaneously record the transcriptional dynamics driven by the *Arc* promoter and the *Egr1* promoter in primary cultured mouse hippocampal neurons (**Extended Data Fig. 7**). ARC and EGR1 were selected for their central and also potentially distinct roles in activity-dependent neuroplasticity: ARC has been shown to modulate synaptic strength via AMPA receptor trafficking and cytoskeletal remodeling, while EGR1 has been reported to drive longer-term gene expression essential for memory consolidation and neuronal adaptation[71]. Although previous studies reported that both can be induced by neuronal depolarization (*e.g.*, via KCl stimulation) or activation of the cAMP signaling pathway (*e.g.*, via FSK stimulation), the long-term temporal correlations of their behaviors within single neurons remain unclear.

To probe the joint transcription dynamics of *Arc* and *Egr1*, we stimulated cultured neurons with either 55 mM KCl for 2 hours or 25 μM FSK for 1 hour on day 17 after transfection of CytoTape constructs (**Extended Data Fig. 7a**), and incorporated sequential HaloTag dye switches to reconstruct continuous temporal trajectories (**Extended Data Fig. 7b**). Following KCl stimulation, we observed three distinct transcriptional response patterns (**Extended Data Fig. 7c** and **d**). Type 1 neurons ("synchronous mode") showed concurrent strong induction of both *Arc* and *Egr1* (**Extended Data Fig. 7c** and **d**, left panel). In Type 2 neurons ("divergent mode"), *Arc* exhibited a biphasic response, with two distinct peaks following a single KCl stimulation, while *Egr1* showed only a weak induction (**Extended Data Fig. 7c** and **d**, middle panel). In contrast, Type 3 neurons ("*Egr1* silent mode") displayed minimal *Arc* and *Egr1* activation (**Extended Data Fig. 7c** and **d**, right panel). These distinctions were confirmed by side-by-side statistical comparisons of single traces among the three groups (**Extended Data Fig. 7e**, **Fig. S18b**). The initiation kinetics of the *Egr1* signal was faster in Type 2 neurons than in Type 3, while no significant difference was observed between Type 1 and Type 2. Type 1 neurons displayed a rapid decline in *Egr1* signal after 3 days of recording, in contrast to the sustained weak response observed in Type 3 neurons. The *Arc* signal in Type 2 neurons peaked twice after stimulation, with the onset and decay of the first peak occurring well before the time

when the *Arc* signal in Type 1 neurons reached their single peaks. Type 3 neurons, on the other hand, showed a more attenuated *Arc* response, distinct from the dynamics observed in Types 1 and 2.

When stimulated with forskolin, we also identified three distinct transcriptional response types, though the signal behaviors differed markedly from those observed under KCl stimulation (**Extended Data Fig. 7f-h**). Type 1 neurons (“synchronous mode”) displayed tightly coordinated induction of both *Arc* and *Egr1* signals (**Extended Data Fig. 7f** and **g**, left panel). In type 2 neurons (“divergent mode”), *Arc* exhibited one strong activation, and *Egr1* expression showed oscillatory waveforms (**Extended Data Fig. 7f** and **g**, middle panel). In contrast, Type 3 neurons (“*Egr1* silent mode”) showed robust *Arc* activations with minimal *Egr1* activation (**Extended Data Fig. 7f** and **g**, right panel). Comparative analysis (**Extended Data Fig. 7h**, **Fig. S18c**) showed that *Egr1* signal intensity was highest in Type 1 neurons, where a single strong peak of *Egr1* activity was observed. In contrast, Type 2 neurons showed oscillatory *Egr1* waveforms at significantly lower signal amplitudes compared to those in Type 1 neurons. For *Arc*, the overall signal strength was similar across all three neuron types. However, in Type 2 neurons, *Arc* signal reached the peak and then returned to baseline faster than that in Type 1 neurons. These correlation analyses provide a temporal perspective to the previously reported regulatory link between *Arc* and *Egr1* in neurons, suggesting a coupling between the timing of *Arc* transcription and the amplitude and the number of *Egr1* transcriptional event(s)[67,68].

The transcriptional modes and waveforms from multiplexed recordings are in agreement with single-activity recordings of *Arc*- and *Egr1*-promoter-driven gene expression, respectively, under identical stimulation conditions (**Fig. S18a**), reporting comparable dynamics of each activity. This result indicates that multiplexing on CytoTape does not alter its ability to record each individual signal, at least at the time scale we validated in this study. In conclusion, these multiplexing results demonstrate the power of CytoTape to dissect within-cell correlations among multiple cellular activities, which are otherwise inaccessible from single-activity recordings alone, facilitating a new kind of study on the complex dynamics and interactions in gene regulation networks as well as their couplings to signaling pathways.

Spatiotemporally resolved, multi-brain-region, single-cell continuous recording *in vivo*

To extend CytoTape’s utility for *in vivo* applications, we first evaluated its performance in the mouse brain via adeno-associated virus (AAV)-mediated gene delivery into the hippocampus. As expected, CytoTape formed thin and flexible fibers in the living mouse brain (**Extended Data Fig. 8a**), which were also significantly thinner than those observed with XRI in *vivo* (**Extended Data Fig. 8b**). This also demonstrates our design strategy works both in cell culture and *in vivo*. However, we found that CytoTape forms

multiple fibers (> 70% of neurons formed over three fibers per soma) within each soma that are closely spaced (**Extended Data Fig. 8c**), which could make conventional microscopy inadequate for accurately resolving and quantifying individual fibers in intact tissue. In comparison, the vast majority of cultured neurons only contain one or two soma-localized CytoTape fibers (**Fig. S6g**). We next performed structural monomer design and screening in the living mouse brain to optimize CytoTape for *in vivo* applications. This round of design and screening aimed to identify a variant that could simultaneously minimize fiber number and maintain thin fiber morphology. We retained the L349K mutation in 1POK for all *in vivo* variants, as it reduces fiber thickness (**Extended Data Fig. 1a**, **Fig. S4**), and further optimized the CytoTape design for use in the living brain by testing linker lengths of 6, 10, 12, and 18 amino acids (**Extended Data Fig. 8b**) between 1POK(E239Y, L349K) and MBP. The resulting construct, CytoTape-vivo with a 6-residue linker, produced only one or two fibers per soma in over 70% of neurons (**Extended Data Fig. 8d**), while maintaining a fiber thickness comparable to CytoTape and significantly thinner than XRI (**Extended Data Fig. 8b**). We performed immunohistochemical-based safety characterizations of CytoTape-vivo in mouse brains and found CytoTape-vivo expression in cell populations *in vivo* does not alter cellular and synaptic state markers, including NeuN as a neuronal marker, cleaved Caspase-3 as an apoptotic marker, GFAP as an astrocyte marker, Synaptophysin as a synaptic protein marker, Iba1 as a microglial marker, γH2AX as a DNA damage marker, Hsp70 and Hsp27 as cell physiological stress markers, and FOS protein as a cellular activity marker (**Extended Data Fig. 9**). Furthermore, we performed animal behavior tests, including open-field test, novel objective recognition test, and contextual fear conditioning, and demonstrated that CytoTape-vivo does not affect neuronal or circuit function *in vivo* (**Extended Data Fig. 10**). Thus, we proceed with CytoTape-vivo as the structural monomer for *in vivo* applications.

We then evaluated the multiplexed recording capability of CytoTape-vivo in the living mouse brain (**Fig. 5a**). We went on to simultaneously record the doxycycline (Dox)-inducible (Tet-On)[72,73] expression history and the *Fos*-promoter-driven monomer expression history (**Fig. 5b**, left panel), because they represent externally controlled and cell activity-dependent transcriptional programs, respectively, allowing us to benchmark CytoTape-vivo's ability to resolve distinct gene expression events *in vivo*. In addition, we performed volumetric confocal imaging of brain tissue sections expressing CytoTape-vivo (**Fig. 5b**, left panel) and developed a high-throughput image segmentation approach for CytoTape-vivo fibers (**Fig. 5b**, right panel; see the full 3D field of view in **Supplementary Video 1**; see methods details in **Methods**), enabling high-throughput extraction and analysis of CytoTape-vivo fibers in 3D tissue images.

We induced seizure behavior in mice via kainic acid (KA) injection on day 10, a known condition to activate *Fos* promoter across multiple brain regions[112], and administered Dox-containing drinking water at the same time, and then perfused the

mice on day 11 following AAV delivery (**Fig. 5c-f**). We observed accumulation of both Dox-dependent monomers (fused with a FLAG tag) and *Fos* signal monomers (fused with a V5 tag) at the two ends of individual fibers (**Fig. 5d**), indicating that CytoTape-vivo recorded both signals within single fibers *in vivo*. We further confirmed this observation from 13,644 CytoTape-vivo fibers in 8,639 neurons in the hippocampus (subregions CA1 and DG; **Fig. 5e**) and posterior parietal cortex (PPC; layer V) (**Fig. 5e**) of the same mouse, all imaged within 75 min under confocal microscopy. The results show that CytoTape-vivo can record multiplexed gene expression histories from large neuron populations across multiple brain regions and be read out via a single confocal snapshot, enabling high-throughput analysis of gene regulation trajectories *in vivo*. We further analyzed the amplitude of *Fos* signal monomers in CA1, DG, and PPC (**Fig. 5f**). We observed that neurons in the DG showed higher fold changes of *Fos* signal monomer level (compared to pre-Dox/KA baseline) than those in CA1 and PPC within the same brain, which is in accordance with previous findings[74] from immunostaining of endogenous FOS expression in mice under KA-induced seizure, although the endogenous FOS expression is regulated by additional mechanisms besides the *Fos* promoter activity, such as enhancer activity[75] and histone acetylation[76]. Unlike immunostaining, which captures only a single time point and thus cannot determine whether a signal is activated, unchanged, or inhibited relative to baseline, CytoTape-vivo provides temporal resolution, enabling direct comparison of post-KA/Dox and baseline signals to support causal inference.

To recover the time axis from CytoTape-vivo recordings, we utilized Dox-dependent monomers as timestamps for *in vivo* applications, capitalizing on their reversible on/off switching, widespread use in *in vivo* applications, simple and non-invasive administration via drinking water or food, and physiological compatibility[77,78]. We validated this concept by inducing seizure behavior in mice via KA injection and administering Dox-containing drinking water, both on day 10, replacing the Dox-containing water with standard drinking water on day 12, and then fixed the mouse brain on day 14 after AAV delivery (**Fig. 5g-j**). For the control group without Dox or KA administration, there is significantly less signal level of Dox-dependent signal monomer and *Fos* signal monomer compared with the experimental group with Dox and KA administration (**Fig. 5h** and **i**, **Extended Data Fig. 8e**). Using the computational approach we previously implemented for timestamps (**Fig. 2**), we paired the event times of Dox ON, Dox OFF, and fixation with spatial locations of the onset of the rise of Dox-dependent monomers, the onset of the decay of Dox-dependent monomers, and fiber termini, and reconstructed the time axis for the recorded signals from *Fos*-promoter-driven monomers (**Fig. 5h**). We recovered the time course of *Fos* signal monomer expression across 4,514 neurons in DG *in vivo* (**Fig. 5j**; see **Extended Data Fig. 8f** for the recovered time course of the Dox-dependent monomer expression). To test whether CytoTape-vivo can record over weeks, we induced seizure behavior in mice via KA

injection and administering Dox-containing drinking water, both on day 10, replacing the Dox-containing water to standard drinking water on day 13, and then fixed the mouse brain on day 18 after AAV delivery (**Fig. 5k**, representative confocal images of CytoTape-vivo are shown in **Extended Data Fig. 8g**). We recovered the time course of *Fos* signal monomer expression across 14,123 neurons in CA1 *in vivo* (**Fig. 5l**; see **Extended Data Fig. 8h** for the recovered time course of the Dox-dependent monomer expression and **Extended Data Fig. 8i** for all 14,123 single traces of *Fos* signal monomer expression waveform). We also successfully mapped the weeks-long recording of *Fos* promoter activity in CA1 in *vivo* (**Fig. 5m** and **n**). These observations provide examples of the unique type of readout CytoTape-vivo empowers, and do not represent a full scientific study that involves extensive experimentation beyond the scope of this technology development work. Together, these results demonstrate the power of CytoTape-vivo for multiplexed, spatiotemporally resolved, large-scale, single-cell high-throughput recording of gene regulation dynamics *in vivo*.

CytoTape shows superior recording performance compared to live GFP imaging. Long-term recording without continuous imaging: Spatial scalability recording across cell population and *in vivo*

CytoTape offers superior spatial scalability compared to live imaging or time-lapse GFP imaging for tracking gene expression across large cell populations over extended time. While GFP imaging requires continuous microscopy and is constrained by the intrinsic trade-off between spatial scale and spatial resolution, photobleaching effect, and motion artifacts in dividing/migrating cells and *in vivo*, CytoTape addresses these issue by *in situ* recording of transcriptional dynamics within intracellular protein assemblies to be read out post hoc. This decoupling from real-time imaging enables simultaneous analysis of large numbers of cells across broad spatial regions using standard post-fixation imaging techniques, making CytoTape particularly well-suited for high-throughput, single-cell-resolved studies over large tissue volumes (**Fig. S19** and **S20**). **Long-term recording without continuous imaging:** Live-cell GFP imaging requires sustained access to advanced microscopy systems and constant environmental control, often introducing phototoxicity and photobleaching over time[79,80]. CytoTape, by contrast, continuously and passively encodes gene expression dynamics intracellularly over days to weeks, without requiring real-time monitoring or extended imaging sessions. **Multiplexing fidelity:** Different fluorescent proteins require different amounts of time to begin exhibiting fluorescence (e.g., ~1-2 h for EGFP; ~3-4 h or more for red-shifted FPs)[81], leading to delayed readouts for multiplexing. These discrepancies complicate multiplexed analysis. CytoTape has sub-hour temporal resolution (**Fig. 2**) and can encode transcriptional activity into monomers (same structure) with unique epitope tags. This enables precise reconstruction of asynchronous events, independent of fluorophore kinetics, exceeding the practical spectral limit of live-cell FP imaging, which

is typically limited to 2–3 non-overlapping channels due to significant crosstalk from FP's wide emission spectral windows[82].

To further demonstrate that CytoTape can faithfully reveal hours- and days-scale transcriptional events that GFP live imaging cannot, we directly compared both methods in tracking CREB expression following forskolin stimulation. As shown in **Fig. S19**, we found that it becomes increasingly difficult to monitor single-cell GFP dynamics over time due to cell division in HEK cells, which disrupts lineage tracking. In contrast, CytoTape reliably recorded CREB transcriptional dynamics at single-cell resolution across thousands of cells with a single confocal image. Thus, compared to GFP, CytoTape enables large-scale, single-cell-resolved readout of gene expression kinetics over extended timescales. Furthermore, we tested the performance of the FOS-GFP live imaging system under unstimulated conditions (**Fig. S20**). We observed that GFP fluorescence intensity gradually increased over time, even in the absence of stimulation. However, immunofluorescence staining showed that the actual FOS protein levels in cells were not detectably changed under the same conditions. This suggests that the GFP system has a significant "baseline drift" artifact likely due to the accumulation of GFP molecules in cells over time from the steady baseline expression, which can interfere with interpretations of the actual gene regulation dynamics. In contrast, CytoTape exhibits a flat baseline in the absence of stimulation (**Fig. S14** and **S16**), minimizing background noise and improving the reliability of stimulus-induced signal recording. We also tested whether live GFP imaging can distinguish two stimulation events separated by a 12-hour interval. To minimize the impact of cell density on single-cell GFP signal tracing during live imaging, we seeded HEK cells at very low density. As shown in **Fig. S21**, CytoTape clearly resolves two distinct peaks, whereas GFP imaging does not. These results demonstrate that CytoTape offers superior temporal resolution compared to live GFP imaging. Thus, the above results demonstrate that CytoTape is far superior to live GFP imaging and indicate that the results shown in **Fig. 3-4** and **Extended Data Fig. 7** cannot be obtained by live GFP imaging.

Discussion, limitation, and future direction

In this study, we developed CytoTape, a genetically encoded, modular protein assembly-based system for multiplexed, analog recording of cellular activities at single-cell resolution and multi-week timescales. Through computationally assisted rational design, CytoTape produces thin, flexible, thread-like protein assemblies that achieve long-term, multiplexed recording in both dividing and post-mitotic cell types, complementing prior systems such as XRI and iPAK4 (**Table S9**, **Fig. S24**). CytoTape is an analog recorder that embeds temporal signals along the spatial dimension, recording continuously over the time axis with the recorded signal intensities across time points being directly comparable. This unique feature enables measurement of complex temporal waveforms, a desired capability for analyzing complex cellular

dynamics. To achieve spatial encoding of the time axis, we fused CytoTape to HaloTag and introduced sequential switches of HaloTag-ligand dyes that served as discrete timestamps on top of the continuously recorded analog signals along CytoTape assembly, with timestamp precision down to minutes-scale. Interpolation of these timestamps and their corresponding real-world time points enabled the reconstruction of individualized, continuous time axes for each cell. We demonstrated that CytoTape can encode transcriptional responses driven by a range of activity-dependent promoters, including immediate early gene activity (*Fos*, *Egr1*, and *Arc*), transcription factor activity (CREB, NPAS4, and FOS), and cellular stress-associated activity (*Hspa1a*), in response to a variety of cellular stimuli such as neuronal depolarization, chemical activation of signaling pathways, and heat shock, across cell types. Furthermore, CytoTape supports simultaneous recording of up to five transcriptional signals within a single fiber and revealed heterogeneous expression dynamics across cell populations. These capabilities establish CytoTape as a powerful platform for spatiotemporally resolved, multiplexed, down to minutes-scale temporal resolution, and weeks-long analog recording in live cells. In contrast to single-timepoint snapshot readout methods, CytoTape provides temporal resolution to enable comparison of signals across history and support causal inference. By enabling multiplexed recording of cellular activities over multi-week timescales, the CytoTape toolkit could open new directions to decode long-term cellular processes—such as tracking the spatiotemporal signatures and differential roles of IEG activities in neurons[83,84], revealing how cellular stress and immune pathways interact within single cancer cells in healthy and diseased states and under drug exposure[85,86], and mapping spatially resolved gene expression histories during cell development or regeneration[87,88], to name a few.

Building on CytoTape, we further optimized the system into CytoTape-vivo, a variant specifically designed for spatiotemporally resolved and scalable single-cell recording in the living brain. CytoTape-vivo retains the core architecture of CytoTape but incorporates drinking water-delivered doxycycline-inducible timestamps to enable noninvasive temporal labeling. We validated CytoTape-vivo by simultaneous recording of Dox-dependent and *Fos*-promoter-driven gene expression dynamics under seizure behavior, capturing over 14,000 neurons in the same brain under ~2 hours of post-fixation confocal imaging time, and observed spatial heterogeneity in *Fos*-promoter-driven gene expression activity over the hippocampus and cortex of single mice. These findings demonstrate that CytoTape-vivo extends CytoTape's capabilities to *in vivo* contexts, enabling long-term gene expression recording with cellular resolution across large spatial volumes *in vivo*.

Recent advances in nucleic acid-based molecular recording have enabled storage of physiological histories within the genome. CRISPR integrase-based systems such as Record–seq[89] and Retro-Cascorder[90] achieve transcriptome-wide RNA-to-DNA conversion and barcode editing. INSCRIBE[91] further preserve spatial and cellular

contexts via *in situ* readout of nucleic acid barcodes in cultured cells, providing a single-signal readout per cell. This system establishes a scalable platform for genetic recording and *in situ* reconstruction of signaling histories in single cells, potentially advancing the quantitative analysis of cell–cell heterogeneity and communication during development and disease. However, these approaches typically do not offer continuous time recording and are primarily optimized for use in prokaryotic cells or mammalian cell cultures. By contrast, CytoTape enables non-destructive, single-cell continuous recording of transcriptional dynamics with temporal resolution down to minutes-scale, recording duration for up to three weeks, and multiplexing capability to allow three-color time encoding and five-color transcriptional dynamics encoding, all on a single CytoTape fiber. CytoTape can be delivered transiently by routine DNA transfection and AAV transduction techniques without genome editing, functions in both post-mitotic and dividing cells, and requires only standard immunostaining and conventional microscopy for readout. Furthermore, CytoTape-vivo enables multiplexed recording of transcriptional activities across multiple brain regions in the living mouse brain at single-cell resolution. While nucleic acid-based methods are powerful for transcriptome profiling and lineage tracing, CytoTape and CytoTape-vivo complement these toolkits by enabling use cases where continuous tracking of one or multiple transcriptional dynamics is desired in mammalian cells *in vitro* and *in vivo*. Existing live-cell and *in vivo* imaging techniques trade-off between resolution and scale. Imaging modalities like MRI, CT, and ultrasound provide brain-wide imaging access but not single cell resolution. High-resolution light microscopy systems provide single cell resolution but suffer from limited field of view size, limited access to deep tissue due to tissue absorption and scattering of light, and the photobleaching effect when imaging fluorescent reporters (usually limited to one or two fluorescent reporters in single cells *in vivo* due to spectral overlap). The CytoTape toolkit breaks this tradeoff between resolution and scale by encoding multiple kinds of cellular activities stably in protein assemblies for readout via post-fixation/post-mortem imaging techniques that support both high resolution and large scale.

Several limitations of the CytoTape toolkit are noted. First, although CytoTape has been demonstrated to simultaneously record up to five dynamic nodes in gene regulatory networks, the complexity of many intracellular processes may require multiplexing beyond five signals to fully dissect the temporal principles in much larger networks. Second, the current system is limited to transcriptional readouts, whereas many other cell physiology-relevant signals, such as signaling activities faster than minutes-scale[92] or translational dynamics[70], are not directly accessible with this platform.

To address these limitations, we envision several future directions below. First, CytoTape could be integrated with other cell activity reporter systems, such as the Fucci system[93] to correlate transcriptional activity with cell cycle progression, and multi-

round immunostaining techniques to visualize many more kinds of molecular tags, thus dramatically increasing the number of simultaneously recordable cellular signals, *i.e.*, the multiplexing capacity, along a single assembly. Expanding the recorder repertoire to include reporters for calcium[94,95] and kinase activities[96] would further broaden CytoTape's utility, while additional protein or system engineering efforts may be required to push the minutes-scale temporal precision down to seconds-scales and beyond for these faster cellular activities. To scale the system to tissue-wide or whole-organism contexts, CytoTape could be paired with systematic gene delivery methods[97] and high-throughput imaging modalities such as light-sheet microscopy. Future works may also combine CytoTape recording with spatial omics readout in tissue, to generate synergistic datasets that leverage the strengths of both kinds of technologies. Incorporating AI-powered analysis pipelines would further enable large-scale, multimodal (space × time × cell type × molecular state × cellular connectivity) reconstructions of gene regulation dynamics at single-cell resolution. Beyond recording, intracellular architectures like CytoTape may have potential to serve as molecular scaffolds for a broad range of future applications, including synthetic cellular barcoding[98], regulation of cellular behavior or enzymatic activity[99], implementation of synthetic gene circuits[100], construction of engineered molecular logic systems[101], and development of human-designed interfaces between cells and external systems[51].

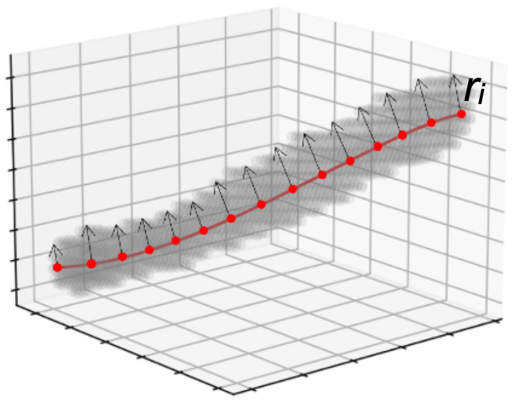

$$D_i = \frac{\sum_{i=1}^{n} r_i}{n} \times 2$$

Supplementary Fig. S1 Quantification of protein fiber width

The fiber was segmented and skeletonized using the algorithms described in the Methods section. The gray, tubular-shaped structure represents the fiber mask in 3D space, with the red line in the center indicating the extracted skeleton. Red dots mark representative skeleton points that are evenly distributed along the skeleton at an interval of 1.5 voxels. r_i denotes the minimum distance from each skeleton point (red dot) to the nearest boundary of the segmented fiber, illustrated by a dark gray arrowed line. D_i denotes the fiber thickness.

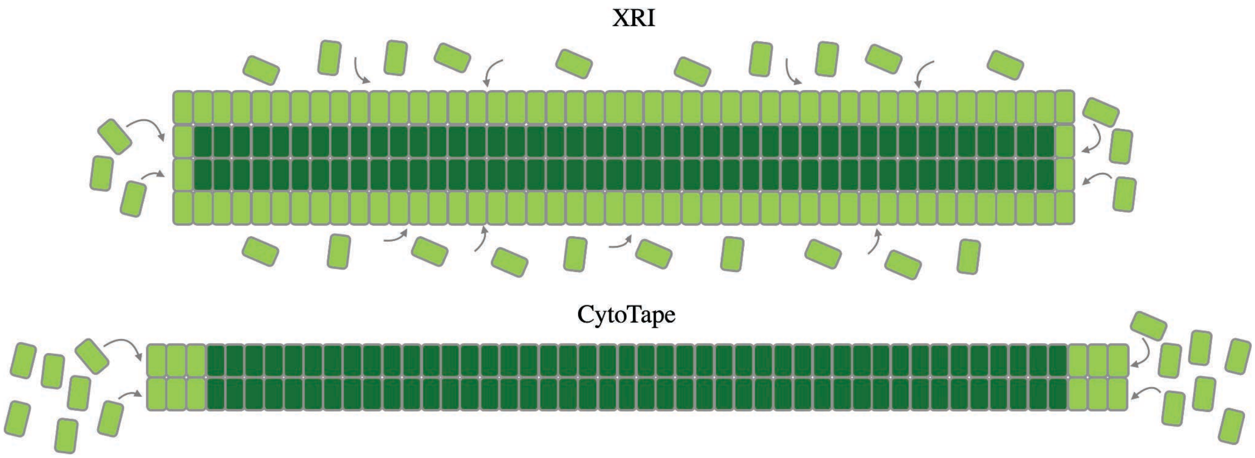

Supplementary Fig. S2 Schematic of structural monomer binding kinetics in XRI and CytoTape during weeks-long recording (after 7 days)

Dark green and light green rectangles represent previously incorporated and newly expressed structural monomers, respectively.

a

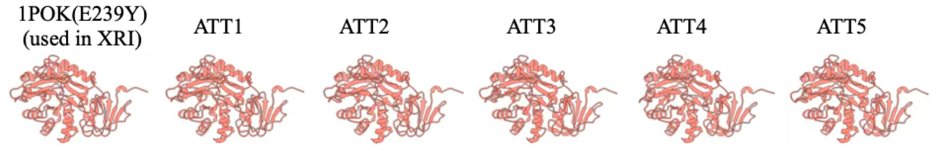

RMSD (Å)\SI	1POK(E239Y)	ATT1	ATT2	ATT3	ATT4	ATT5
1POK(E239Y)		0.767	0.767	0.790	0.733	0.803
ATT1	0.203		0.718	0.759	0.692	0.733
ATT2	0.2	0.237		0.746	0.705	0.754
ATT3	0.196	0.238	0.117		0.71	0.767
ATT4	0.267	0.337	0.274	0.262		0.703
ATT5	0.163	0.243	0.207	0.221	0.283	

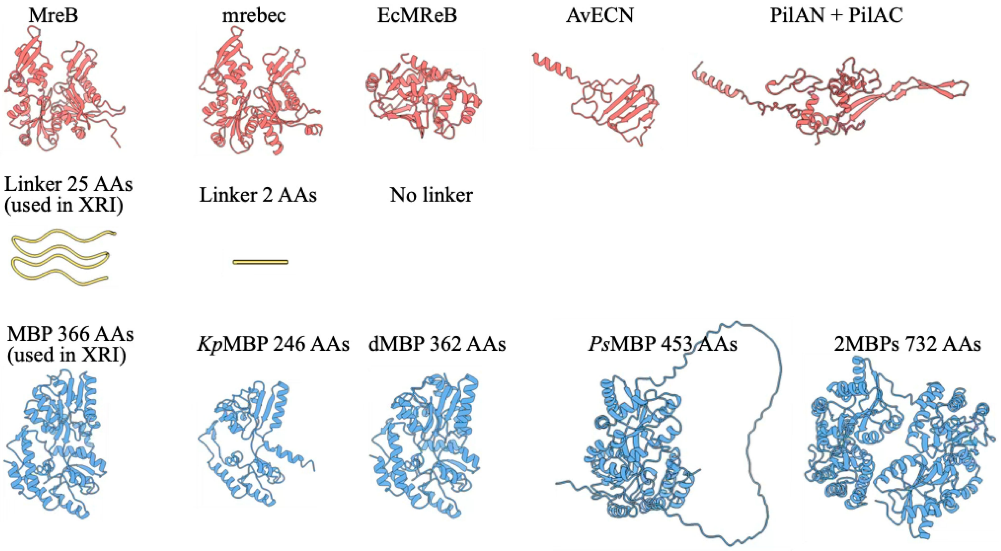

b

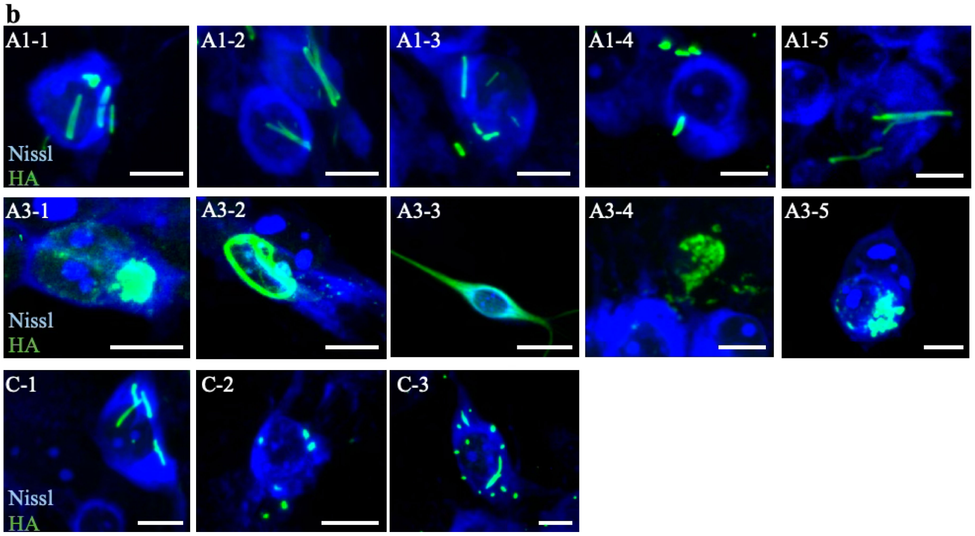

Supplementary Fig. S3 Protein assembly design screening and characterization in cultured neurons

(**a**) Atomic structures used in monomer designs. First row, five artificial scaffolds (artificial ticker tapes (ATTs); used in designs A1-1 to A1-5) generated by *CPDiffusion* for replacing 1POK(E239Y). The table below shows the sequence identity (SI) and root mean square deviation (RMSD) between each pair of scaffolds. The *CPDiffusion*-generated structures share a similar architecture with 1POK(E239Y), but vary in the amino acid sequences. Second row, natural scaffold for replacing 1POK(E239Y) (used in designs A3-1 to A3-5). Third row, linker length optimization (used in designs B-1 and C-4); fourth row, MBP homologs identified via BLAST search and their engineered variants. Note that the 2MBPs shown in the third row represent a direct fusion of two MBPs (366 AAs) (used in designs C-1 to C-5). All the atomic structures are predicted by AlphaFold3. (**b**) Confocal images of cultured mouse hippocampal neurons expressing design variants fused to the HA tag, and after fixation on day 7, Nissl staining, and immunostaining against the HA tag. The constructs transfected into neurons are listed in **Supplementary Table S1**. Scale bars, 10 µm.

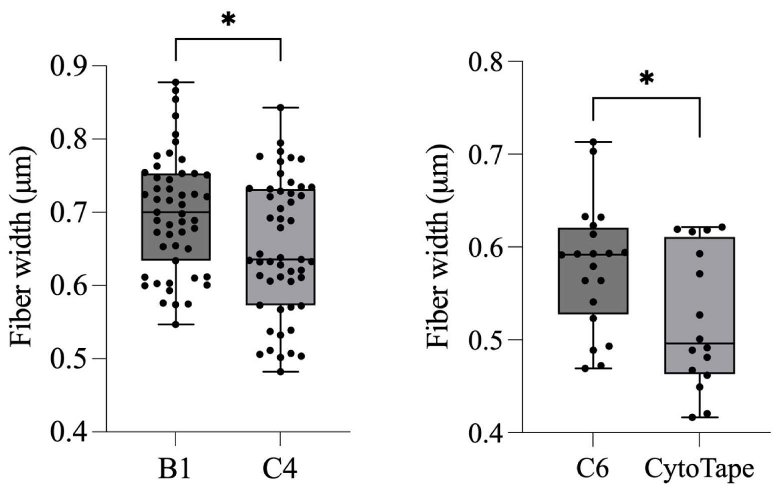

Supplementary Fig. S4 Statistical analysis of protein assembly width for B1 and C4 (left panel) and C6 and CytoTape (right panel)

Left panel, A total of 51 fibers from B1 and 51 fibers from C4 were analyzed. Right panel, A total of 20 fibers from C6 and 16 fibers from CytpTape were analyzed. *, $P < 0.05$; Mann–Whitney U test. Middle line in box plot, median; box boundary, interquartile range; whiskers, minimum and maximum; black dots, individual data points.

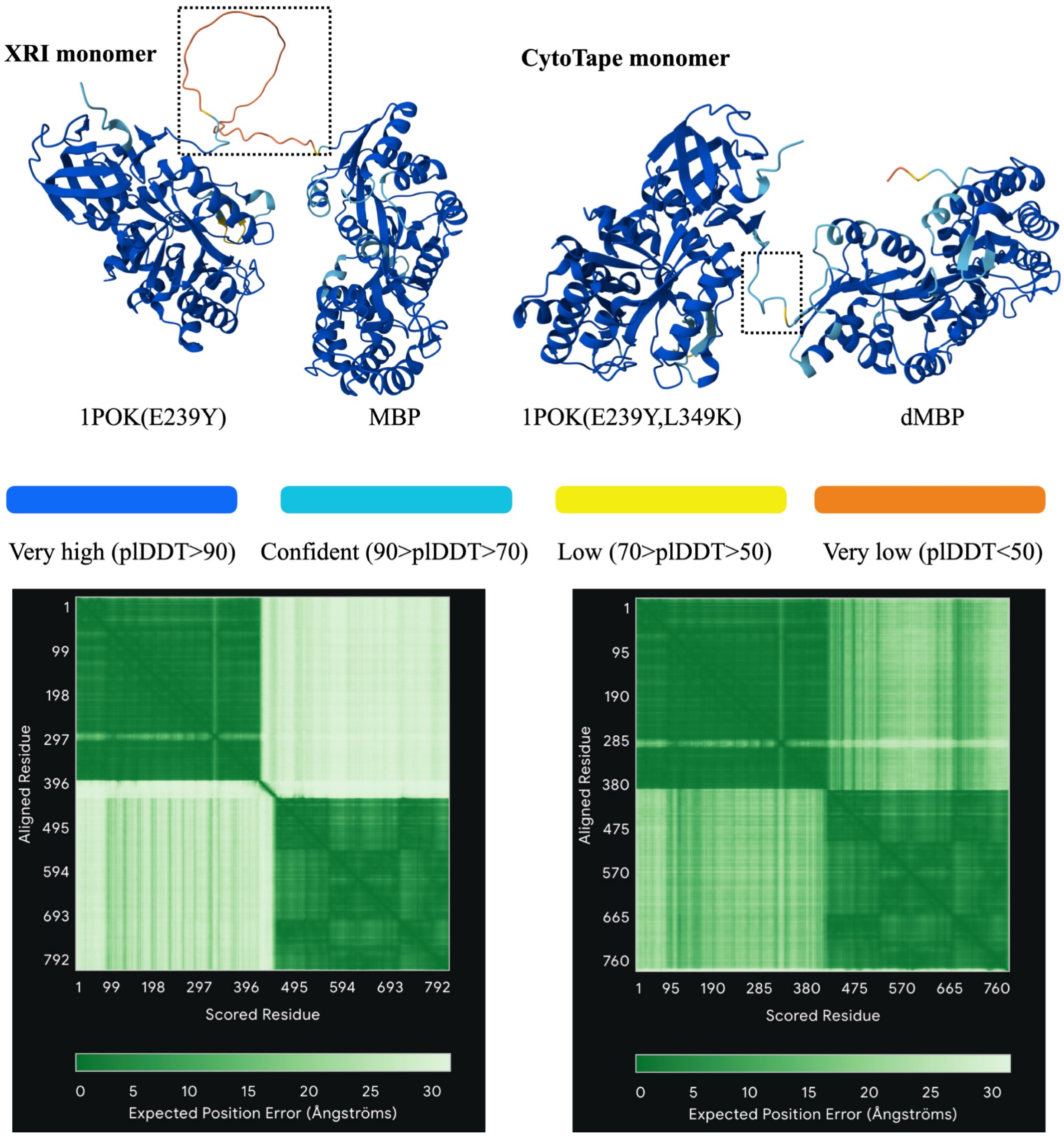

Supplementary Fig. S5 Confidence analysis of AlphaFold3-predicted models for XRI and CytoTape monomers

The dashed black rectangle highlights the linker (amino acid structure) between the two domains. Lower panels represent the PAE heatmap of XRI monomer (left panel) and CytoTape monomer (right panel). The linker25, HA epitope tag, and linker3 are located at positions 391 to 427 in the XRI monomer. Overall iPTM of XRI monomer and CytoTape monomer is 0.55 and 0.7, respectively. All the panels are visualized by the AF3 online system at alphafoldserver.com.

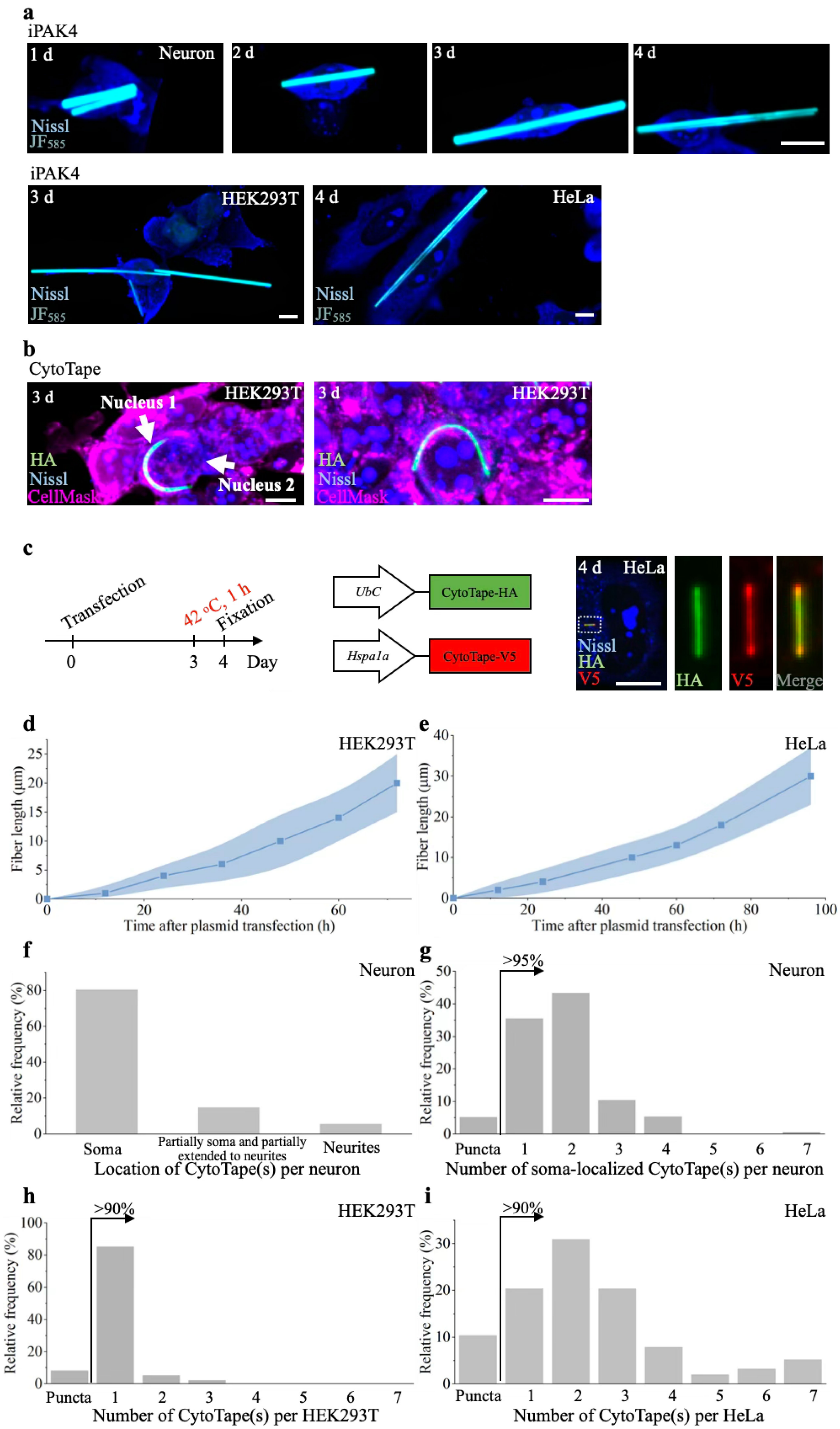
a
iPAK4
1 d
Neuron
2 d
3 d
4 d
Nissl
JF585
iPAK4
3 d
HEK293T
4 d
HeLa
b
CytoTape
Nucleus 1
Nucleus 2
HA
CellMask
c
Transfection
42 oC, 1 h
Fixation
0
3
4
Day
UbC
CytoTape-HA
Hspa1a
CytoTape-V5
V5
Merge
d
e
Fiber length (μm)
Time after plasmid transfection (h)
f
g
Neuron
Relative frequency (%)
Soma
Partially soma and partially extended to neurites
Neurites
Location of CytoTape(s) per neuron
>95%
Puncta
Number of soma-localized CytoTape(s) per neuron
h
i
>90%
Number of CytoTape(s) per HEK293T
Number of CytoTape(s) per HeLa

Supplementary Fig. S6 Analysis of iPAK4 and CytoTape fiber formation in cultured neurons, HEK, and HeLa cells

(**a**) Representative confocal images of cultured mouse hippocampal neurons (top panels), as well as HEK and HeLa cells (bottom panels) expressing iPAK4 with iPAK4-HaloTag. Cells were labeled with JF_{585} dye added to the culture, fixed at different time points, and subsequently stained with Nissl. Scale bars, 10 μm. (**b**) shows CytoTape fibers in dividing HEK cells. The left panel shows a HEK cell undergoing division, and the right panel shows the completion of cell division. (**c**) Left panel, the *Hspa1a* promoter-driven expression experiment timeline. Middle panel, the constructs transfected into HeLa cells. Right panel, confocal image of HeLa cell expressing CytoTape-based constructs, taken after fixation on day 4, Nissl staining and immunostaining against the HA tag and the V5 tag. Scale bar, 10 μm. The three rows of rectangular panels on the right are enlarged views of the regions marked by white dashed rectangles in the left column of square panels. The growth kinetics of CytoTape in (**d**) HEK cell and (**e**) HeLa cell. 20 CytoTapes from two cell cultures were analyzed for length calculations. Blue boundary, s.d. (**f**) Histogram of the number of CytoTapes located in the neuron soma, partially soma and partially extended to neurites, and neurites (n = 50 neurons from five cultures). (**g**) Histogram of the number of soma-localized CytoTapes per neuron (n = 47 neurons from four cultures). '> 95%' with an arrow, > 95% of the neurons have CytoTape(s) rather than puncta. Histogram of the number of CytoTapes per (**h**) HEK cell or (**i**) HeLa cell (n = 69 HEK cells from six cultures, n = 40 HeLa cells from two cultures). '> 90%' with an arrow, > 90% of the cells have CytoTape(s) rather than puncta.

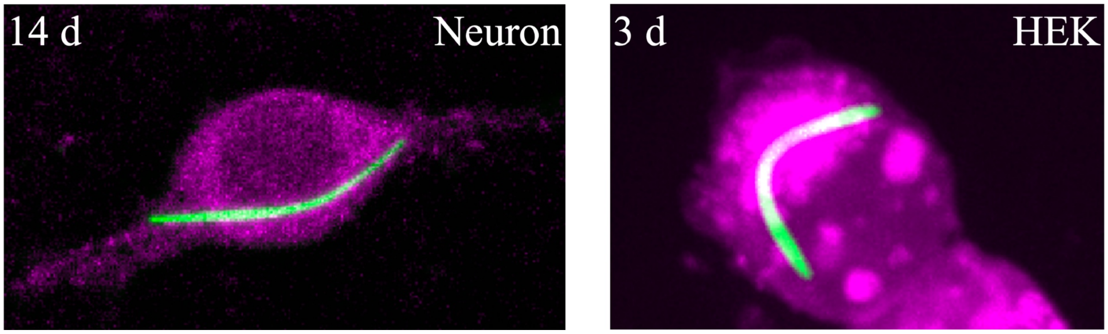

Supplementary Fig. S7 CytoTape does not deform cell membranes or alter cell morphology

CytoTape was expressed in cultured neurons (left panel) and HEK cells (right panel) for 14 days and 3 days, respectively. CytoTape was visualized by HA tag immunostaining (green), and CellMask staining (magenta) was used to label cell morphology.

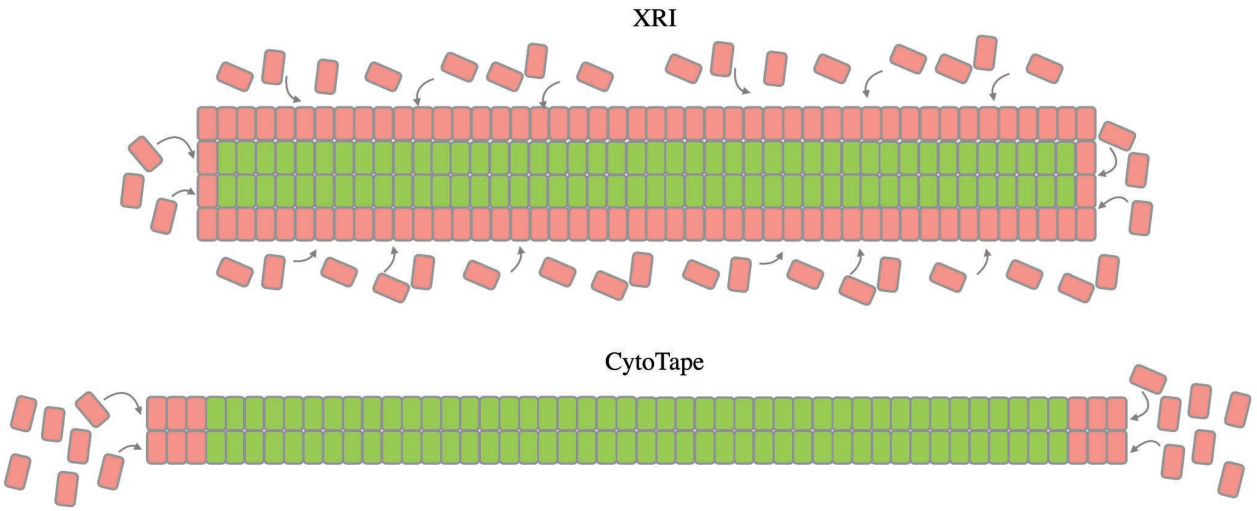

Supplementary Fig. S8 Schematic of signal monomer binding kinetics of XRI and CytoTape during weeks-long recording (after 7 days)

Green and red rectangles represent structural monomers and signal monomers, respectively.

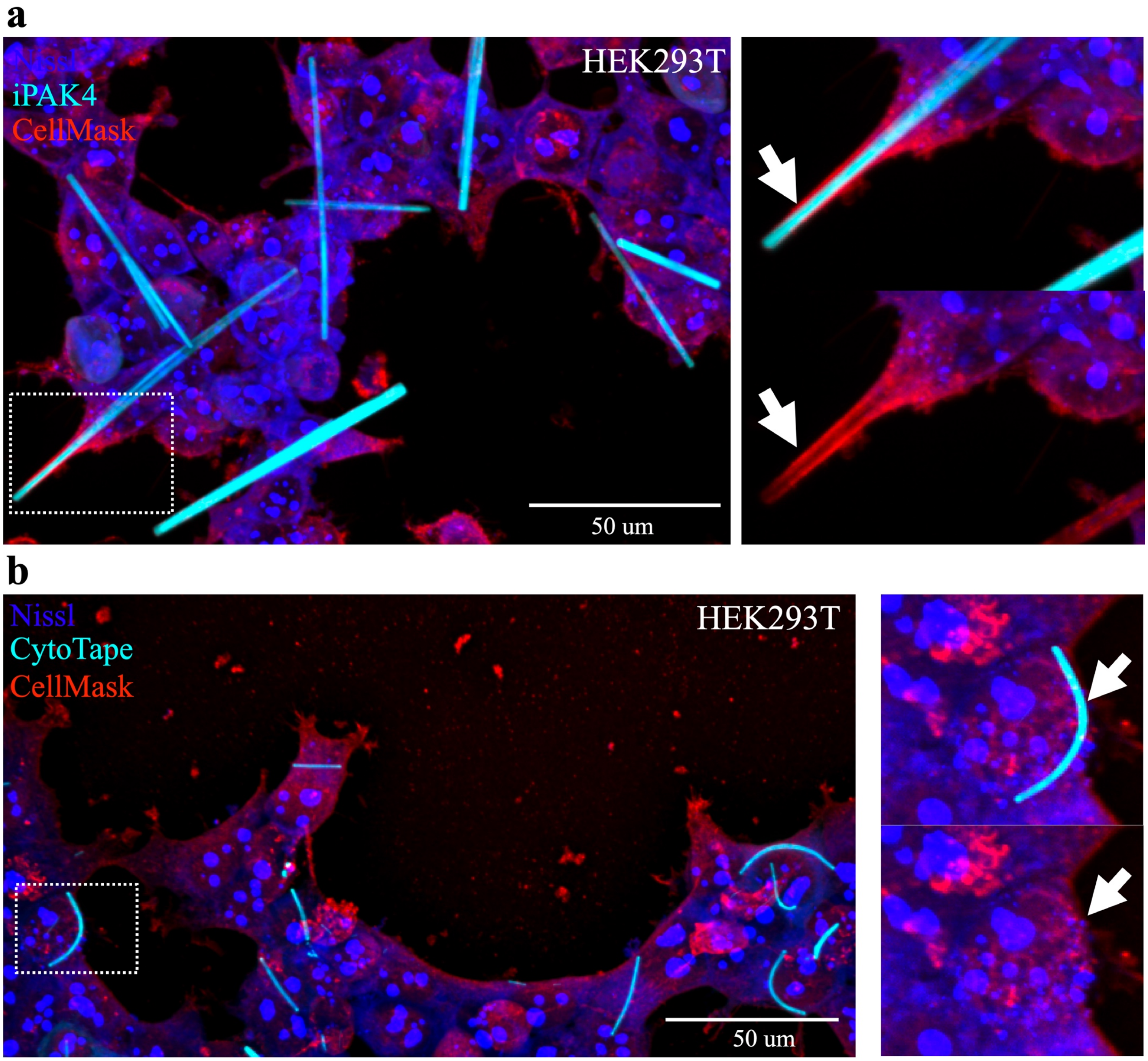

Supplementary Fig. S9 iPAK4 deforms cell membranes and alters cell morphology, whereas CytoTape does not

(**a**) iPAK4 (cyan) and (**b**) CytoTape (cyan) were expressed for 3 days in HEK cells. Nissl (blue) and CellMask (red) staining were used to label cell morphology. The right panel shows an enlarged view of the dashed rectangle in the left panel. iPAK4 was visualized using the JF_{585} dye, and CytoTape was visualized using HA tag antibody immunostaining. Scale bar, 50 µm.

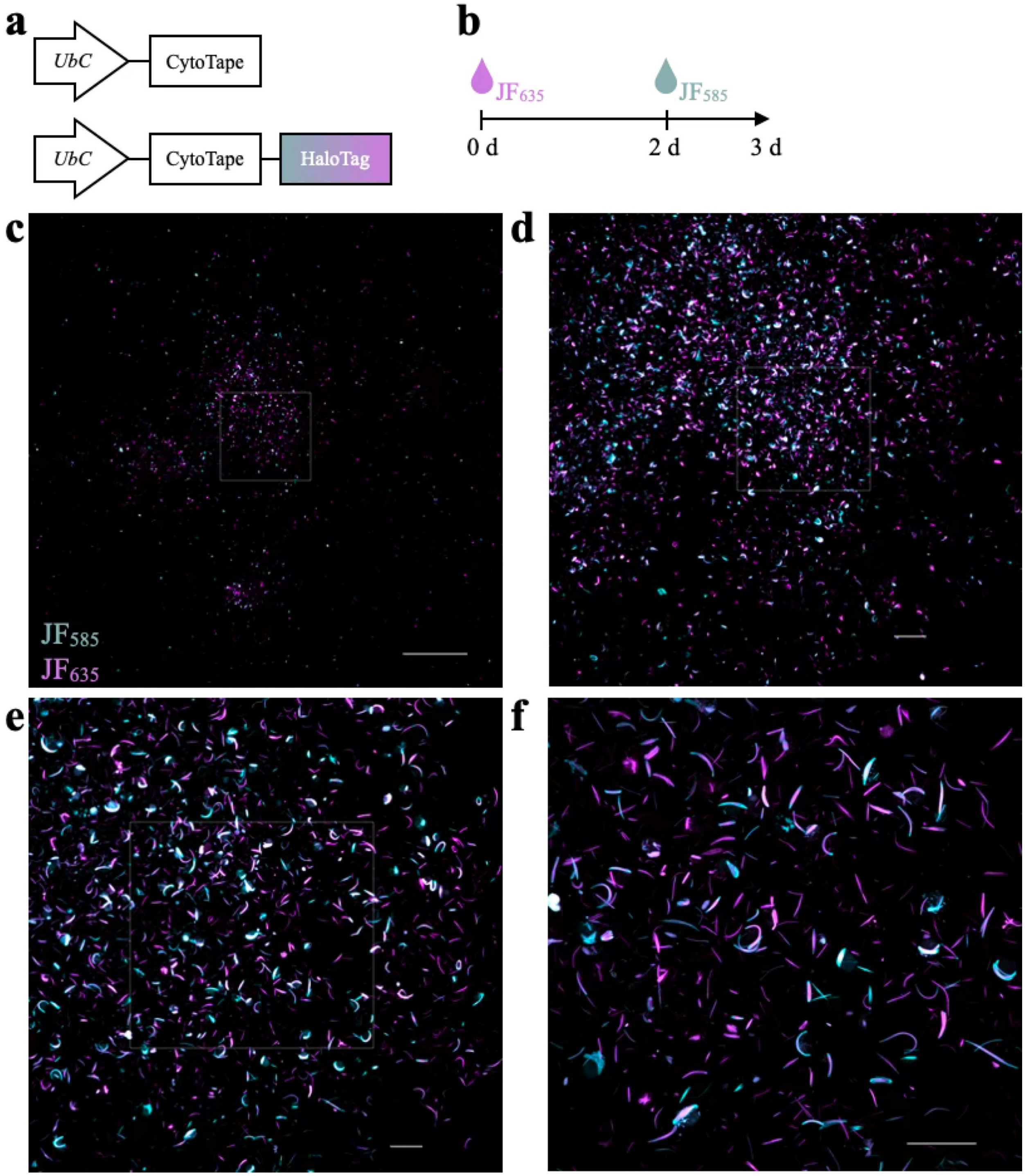

Supplementary Fig. S10 Scalable temporal labeling readout of CytoTape in HEK cells

(**a**) Schematic of the constructs transfected into HEK cells. (**b**) Time points of JF_{585} and JF_{635} addition and fixation. The JF_{585} and JF_{635} dyes were used for labeling time within the CytoTape. Images of CytoTape labeled with JF_{585} and JF_{635} in HEK cells, taken

after fixation on day 3. The scale bar in (**c**) represents 500 μm, the scale bar in (**d**) represents 100 μm, and the scale bars in (**e**) and (**f**) represent 50 μm. The objective lenses used for (**c**), (**d**), (**e**), and (**f**) were 4×, 10×, 20×, and 40×, respectively. Some fibers do not display clear timestamp transitions in these low-magnification images because the optimal image contrast for each color channel to clearly visualize the timestamp transitions varies across individual cells.

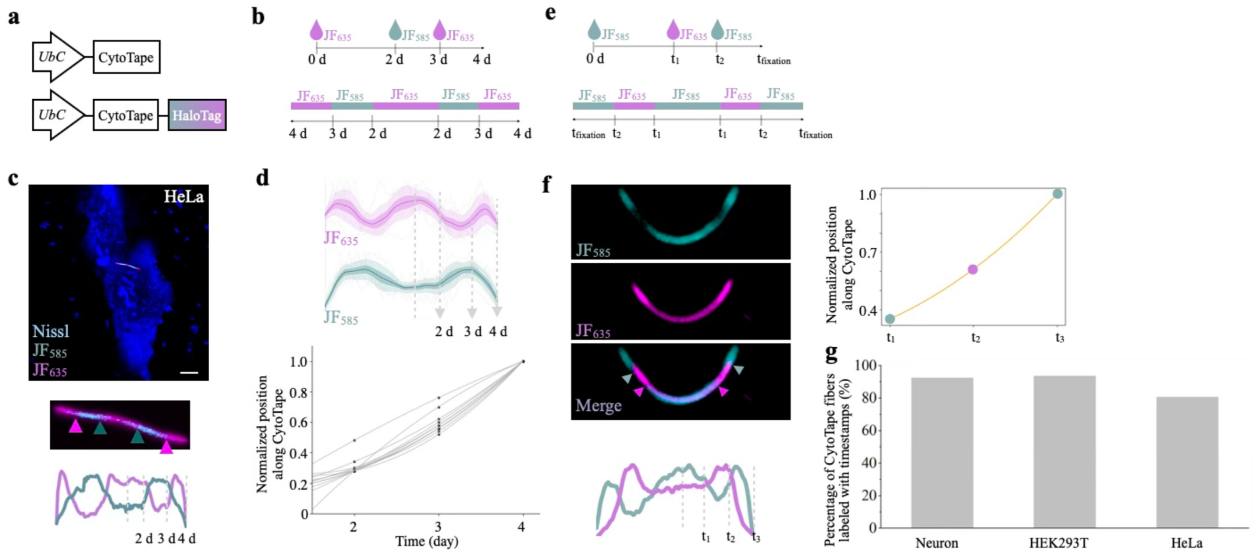

Supplementary Fig. S11 CytoTape encodes temporal information in HeLa cells, HEK cells, and cultured neurons

(**a**) Schematic of the constructs transfected into HeLa cells. (**b**) Top panel, time points of JF_{585} and JF_{635} addition and fixation. Bottom panel, expected dye distribution along the CytoTape. The JF_{585} and JF_{635} dyes were used for labeling time within the CytoTape. (**c**) A representative confocal image of CytoTapes with timestamps in HeLa cells, which is taken after fixation on day 4 and Nissl staining. The middle row and bottom row show the CytoTape with timestamps and fluorescence line profiles, respectively. Scale bar, 10 µm. (**d**) Top panel, statistical analysis of fluorescence line profiles from the experiments described in (**b**) n = 8 CytoTapes from 8 HeLa cells from two cultures. Bottom panel, interpolation between the spatial axis along the fiber and the time axis using the timestamps from top panel. Time axis recovery via B-spline curve fitting of timestamps and fixation point. Each raw trace was normalized to its peak to show relative changes before averaging. Thick centerline, mean; darker boundary, s.e.m.; lighter boundary, s.d.; lighter thin lines, data from individual CytoTapes. (**e**) Time points of dye addition and fixation and expected dye distribution along the CytoTape. (**e**) JF_{585} and JF_{635} were used for labeling time within the CytoTape. (**f**) Images of timestamps with two dye switches. The right panel shows fluorescence line profiles and fitted curves, respectively. The details of the interpolation between the spatial axis along the fiber and the time axis using timestamps are described in Methods. (**g**) Percentage of CytoTape fibers labeled with timestamps in cultured neurons, HEK, and HeLa cells (n = 40 CytoTapes from 40 neurons from five cultures; n = 50 CytoTapes from 50 HEK cells from three cultures; n = 30 CytoTapes from 30 HeLa cells from two cultures).

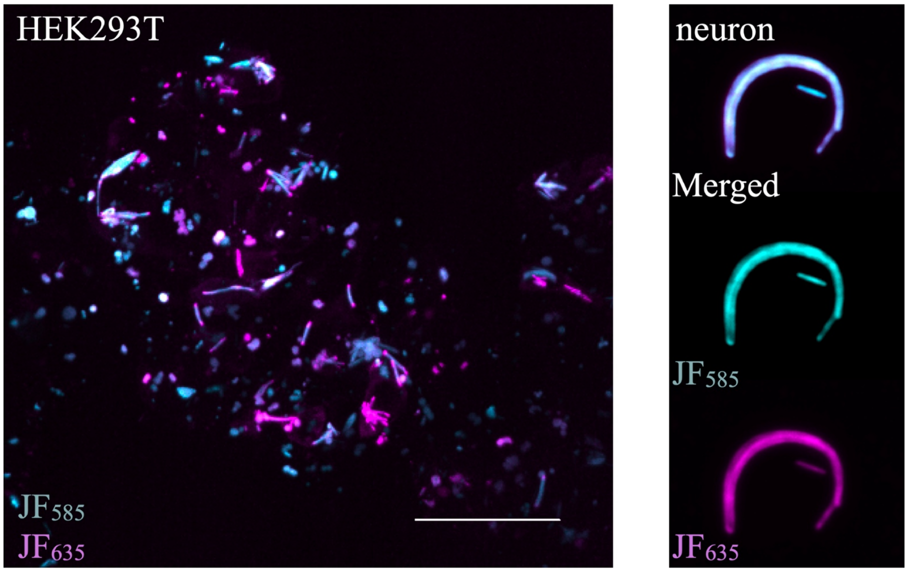

Supplementary Fig. S12 XRI loses temporal information in HEK for 3 days and cultured neurons for 15 days

Left panel, JF_{585} and JF_{635} were added to HEK cell cultures on day 0 and day 2, respectively, and cells were fixed on day 3 after transfection. Scale bar, 50 µm. Right panel, JF_{585} and JF_{635} were added to neuronal cultures on day 5 and day 10, respectively, and neurons were fixed on day 15 after transfection.

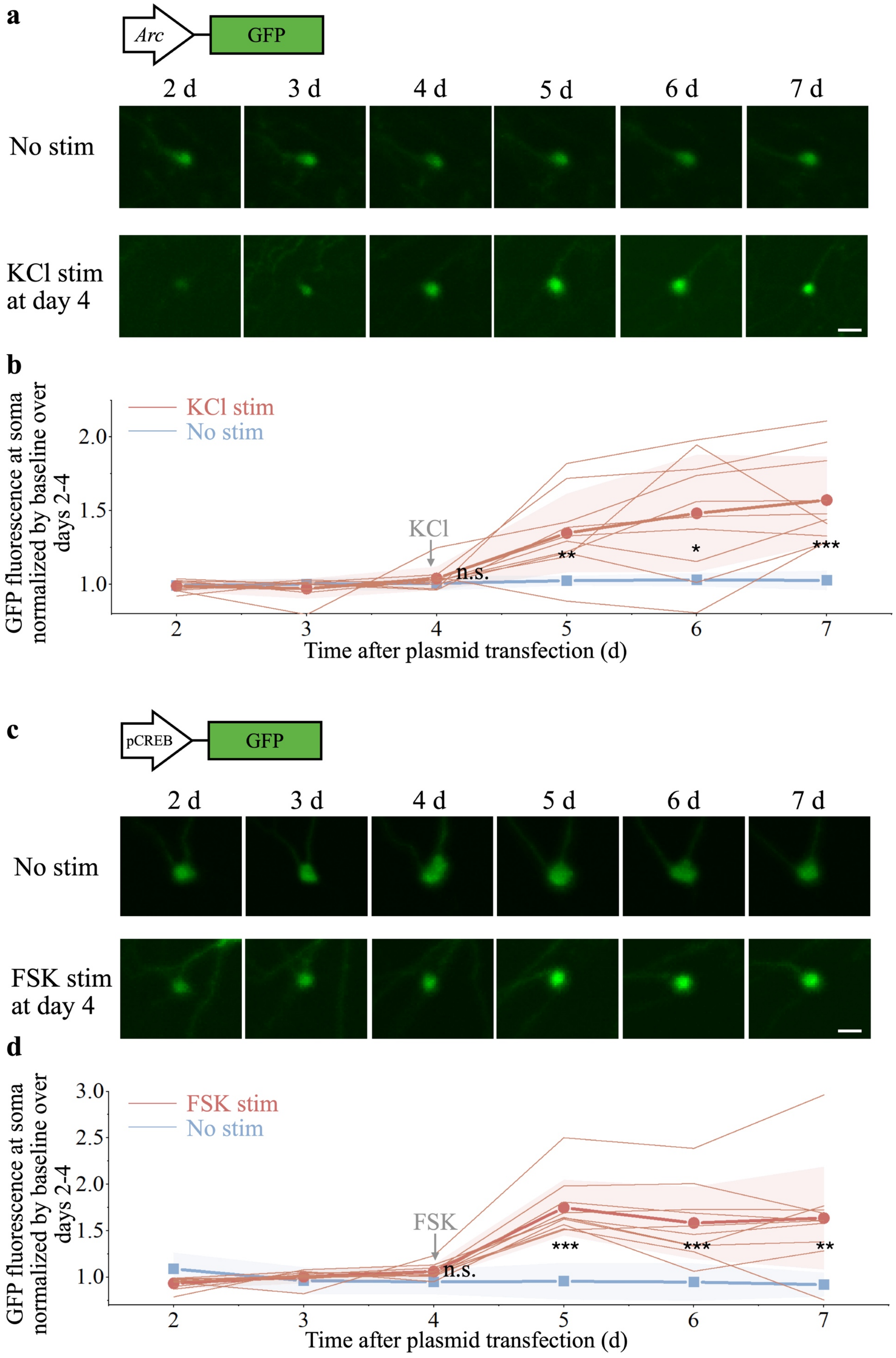

a
Arc
GFP
2 d
3 d
4 d
5 d
6 d
7 d
No stim
KCl stim
at day 4
b
GFP fluorescence at soma
normalized by baseline over
days 2-4
KCl stim
No stim
2.0
1.5
1.0
KCl
n.s.
**
*
***
2
3
4
5
6
7
Time after plasmid transfection (d)
c
pCREB
GFP
2 d
3 d
4 d
5 d
6 d
7 d
No stim
FSK stim
at day 4
d
GFP fluorescence at soma
normalized by baseline over
days 2-4
FSK stim
No stim
3.0
2.5
2.0
1.5
1.0
FSK
n.s.
***
***
**
2
3
4
5
6
7
Time after plasmid transfection (d)

Supplementary Fig. S13 Time courses of *Arc*-promoter-driven expression and CREB activity in cultured neurons measured by timelapse imaging of GFP reporter

(**a**) Construct schematic of GFP driven under *Arc*-promoter and representative confocal images of live cultured mouse hippocampal neurons in the GFP channel 2-7 days (2d-7d) after plasmid transfection, without (upper row) and with (lower row) 55 mM KCl stimulation for 1 hour on day 4. All images were captured under the same imaging conditions. Scale bar, 10 µm. (**b**) GFP fluorescence at soma (normalized by the average GFP fluorescence at soma over days 2-4) versus time (n = 10 neurons from 2 cultures for “No stim” group; n = 10 neurons from 2 cultures for “KCl stim” group). Thick centerline, mean; lighter boundary, s.d.; thin light lines, individual data for stimulation group. *, $P < 0.05$; **, $P < 0.01$; ***, $P < 0.001$; n.s., not significant; Mann–Whitney U test. See **Supplementary Table S11** for details of statistical analysis. (**c**) Construct schematic of GFP driven under pCREB-promoter and representative confocal images of live cultured mouse hippocampal neurons in the GFP channel 2-7 days (2d-7d) after plasmid transfection, without (upper row) and with (lower row) 15 µM FSK stimulation for 1 hour on day 4. All images were captured under the same imaging condition. Scale bar, 10 µm. (**d**) GFP fluorescence at soma (normalized by the average GFP fluorescence at soma over days 2-4) versus time (n = 10 neurons from 2 cultures for “No stim” group; n = 10 neurons from 2 cultures for “FSK stim” group). Thick centerline, mean; lighter boundary, s.d.; thin light lines, individual data for stimulation group. *, $P < 0.05$; **, $P < 0.01$; ***, $P < 0.001$; n.s., not significant; Mann–Whitney U test. See **Supplementary Table S11** for details of statistical analysis.

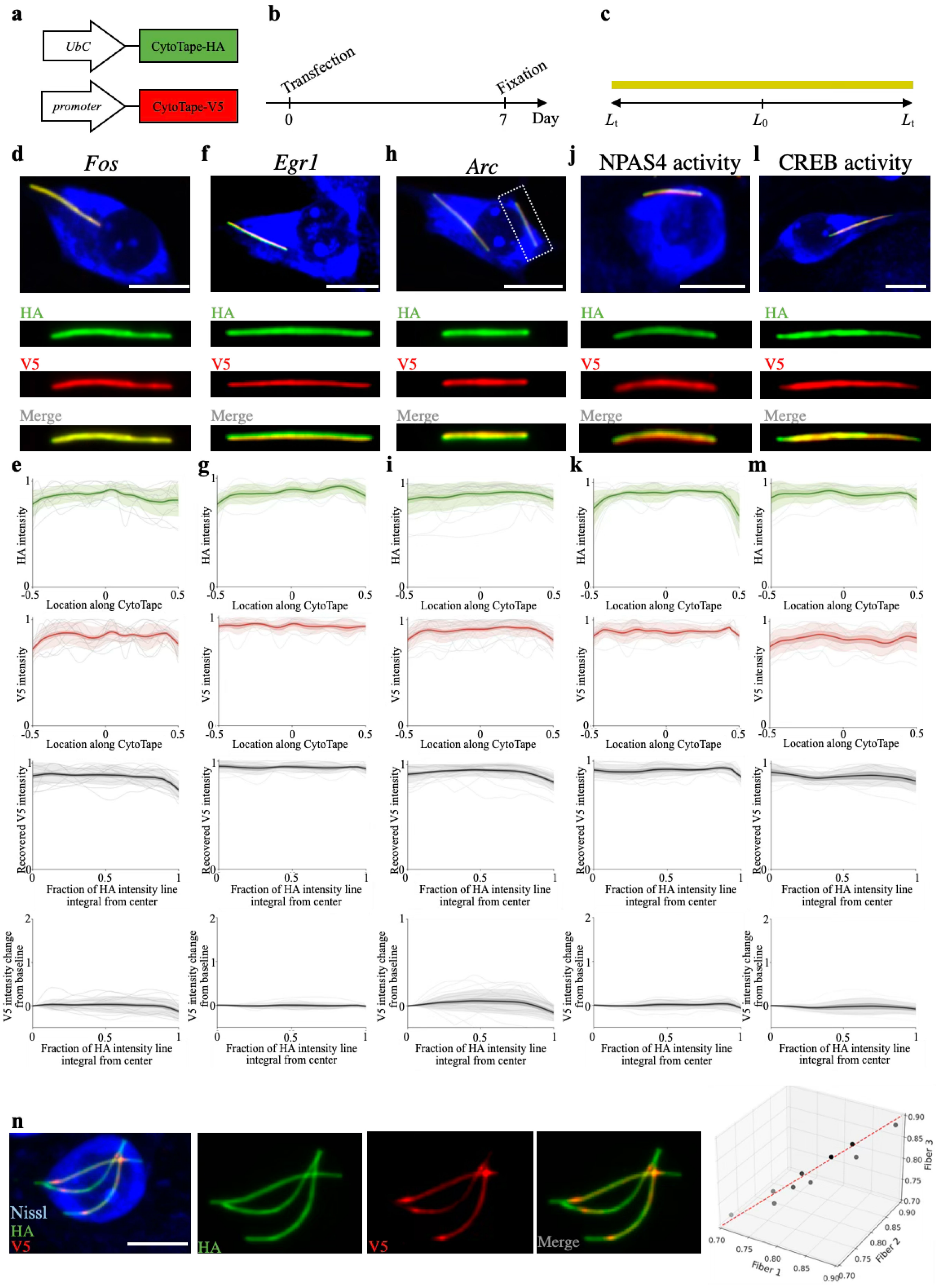
a
UbC
CytoTape-HA
promoter
CytoTape-V5
b
Transfection
Fixation
0
7
Day
c
Lt
L0
Lt
d
Fos
f
Egr1
h
Arc
j
NPAS4 activity
l
CREB activity
HA
V5
Merge
e
g
i
k
m
HA intensity
Location along CytoTape
V5 intensity
Recovered V5 intensity
Fraction of HA intensity line integral from center
V5 intensity change from baseline
n
Nissl
HA
V5
Merge
Fiber 1
Fiber 2
Fiber 3

Supplementary Fig. S14 Control experiments and cross-validation of transcriptional recorders for tracking gene regulation dynamics with CytoTape

(**a**) Schematic of the constructs transfected into neuron cultures and (**b**) the experimental timeline. (**c**) Expected HA tag and V5 tag distribution along the CytoTape. (**d**), (**f**), (**h**), (**j**), and (**l**) Representative images of cultured neurons expressing the constructs shown in (**a**). Images were captured after fixation, Nissl staining, and immunostaining for HA and V5 tags. Enlarged views of the CytoTape in the top-row panels are shown in the three rows of rectangular panels below. Scale bars, 10 μm. (**e**), (**g**), (**i**), (**k**), and (**m**) Profiles of HA and V5 signal intensities along CytoTape, based on the experiment in (**b**)). First row, HA intensity profile; second row, V5 intensity profile; third row, recovered V5 signal, calculated from the intensity profiles, plotted as a function of the fraction of the HA intensity line integral; fourth row, V5 signal relative to baseline (calculated as the ratio of V5 signal to the center V5 signal) plotted as a function of the fraction of the HA intensity line integral. In the first three rows, raw traces were normalized to their peaks to highlight relative changes before averaging. Data were from *Fos*, n = 12 CytoTapes from 12 neurons from two cultures; *Egr1*, n = 13 CytoTapes from 13 neurons from two cultures; *Arc*, n = 24 CytoTapes from 24 neurons from three cultures; NPAS4 activity, n = 11 CytoTapes from 11 neurons from two cultures; CREB activity, n = 9 CytoTapes from 9 neurons from two cultures. Thick centerline, mean; darker boundary near the centerline, s.e.m.; lighter boundary, s.d.; thin light lines, individual CytoTape data. (**n**) A confocal image of a neuron showing three fibers. The experimental conditions are the same as in (**d**). The right panel shows the V5 signal peak position plotted against the HA signal. Each gray sphere represents a neuron (n = 10). The red dashed line represents x = y = z. Scale bar, 10 μm.

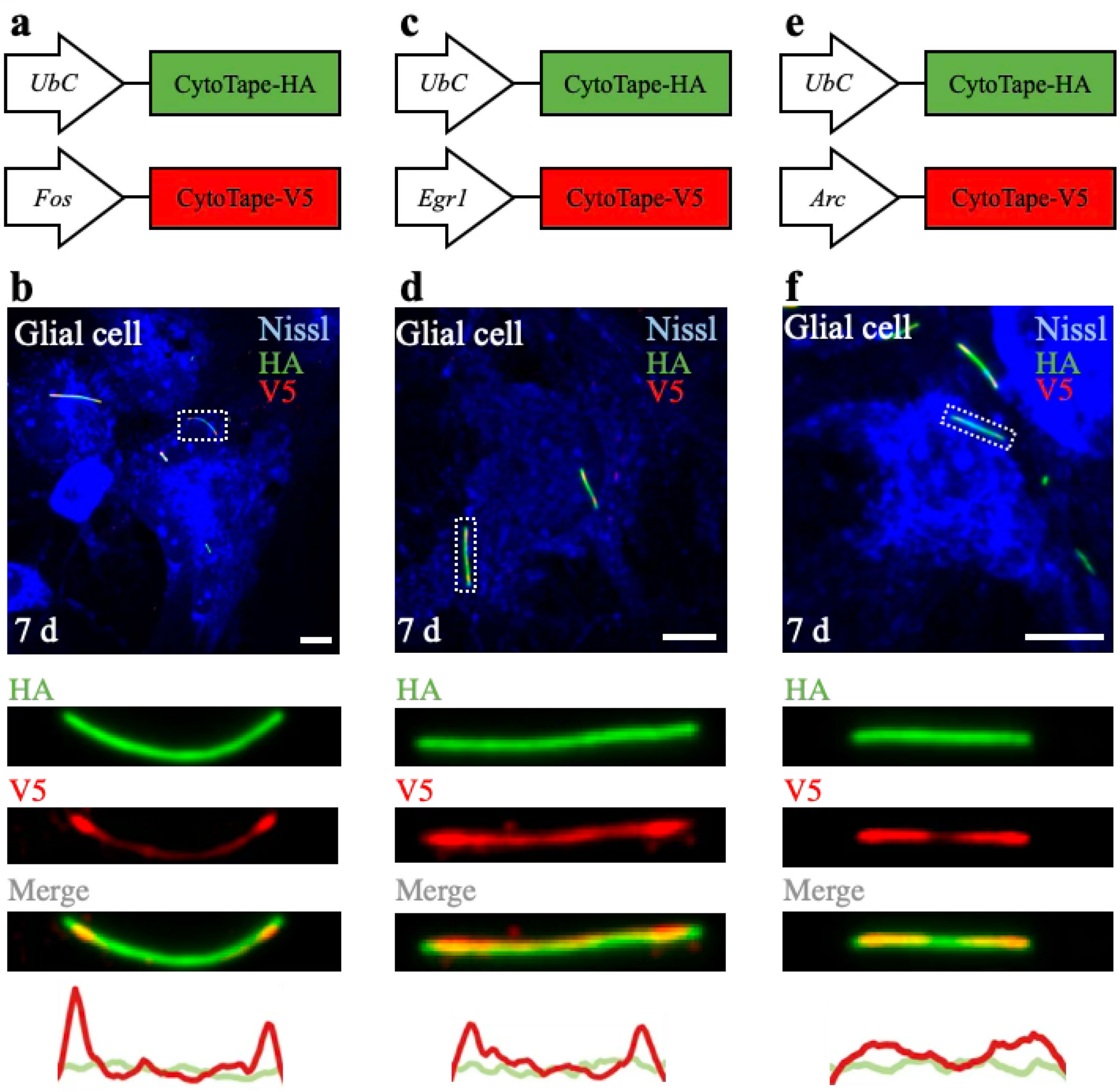

Supplementary Fig. S15 CytoTape enables waveform recording of *Fos*-, *Egr1*-, and *Arc*-promoter activities in cultured mouse hippocampal glial cells under chemical stimulation

(**a**), (**c**), and (**e**) Schematic of the constructs transfected into cultured mouse hippocampal glial cells. CytoTape-HA refers to CytoTape fused to the HA tag, and CytoTape-V5 refers to CytoTape fused to the V5 tag. (**b**), (**d**), and (**f**) Representative images of cultured mouse hippocampal glial cells expressing the constructs shown in (**a**), (**c**), and (**e**). Images were captured after fixation, Nissl staining, and immunostaining for HA and V5 tags. Glial cells were stimulated with 55 mM KCl for 1 h on day 5 and fixed on day 7. Enlarged views of the white-marked regions in the top-row panels are

shown in the three rows of rectangular panels below. The bottom row shows the fluorescence line profiles of HA and V5 tags. Scale bars, 10 μm.

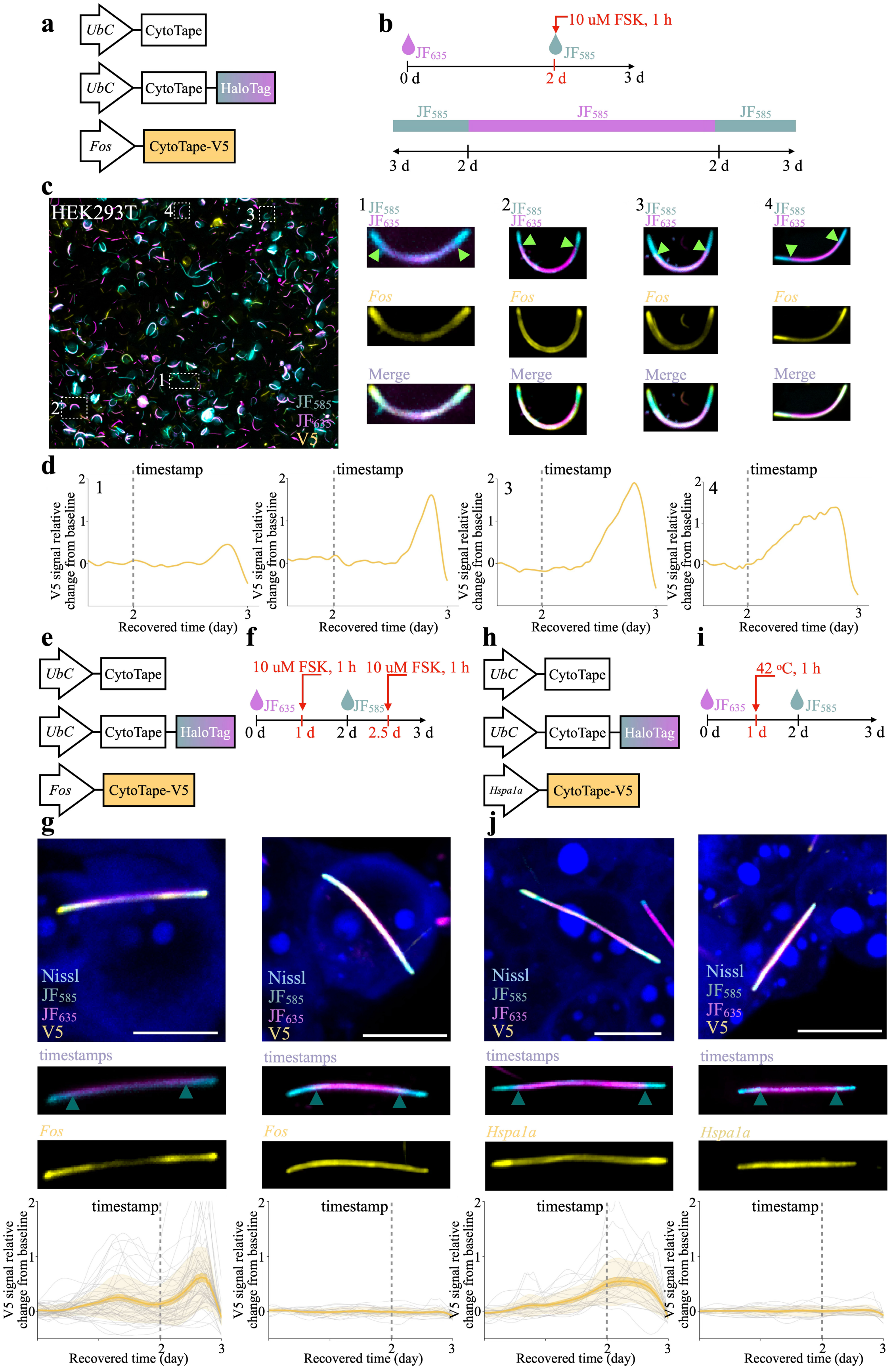
a
UbC
CytoTape
HaloTag
Fos
CytoTape-V5
b
10 uM FSK, 1 h
JF635
JF585
0 d
2 d
3 d
c
HEK293T
JF585
JF635
V5
Fos
Merge
d
timestamp
V5 signal relative change from baseline
Recovered time (day)
e
f
10 uM FSK, 1 h
2.5 d
h
Hspa1a
i
42 oC, 1 h
g
j
Nissl
timestamps

Supplementary Fig. S16 CytoTape enables recording of *Fos*- and *Hspa1a*-promoter activities in HEK cells

(**a**) Schematic of the constructs transfected into HEK cells. (**b**) Top panel, time points of JF_{585} and JF_{635} dyes addition and stimulation (10 μM FSK for 1 h). Bottom panel, expected dye distribution along the protein fiber. The JF_{585} and JF_{635} dyes were added to the HEK cell culture on day 0 and day 2, and fixation was performed on day 3. (**c**) Left panel, low magnification images of CytoTape in HEK cells, which were taken after fixation on day 3, and Nissl staining and immunostaining against the V5 tag. Right panel, enlarged confocal images of CytoTapes indicated in left panel. Arrows indicate the positions of dye switches within the protein fiber. (**d**) *Fos* promoter signal (from (**c**)) relative change from baseline plotted against recovered time (interpolated with timestamps and fixation time) after plasmid transfection. (**e**,**h**) Schematic of the constructs transfected into HEK cells and (**f,i**) the experimental timeline. The JF_{585} and JF_{635} dyes were added to the HEK cell culture on day 0 and day 2, and fixation was performed on day 3. (**g**) and (**j**) Images of HEK cells expressing the constructs shown in (**e**) and (**h**) after stimulation with FSK for 1 h at different time points (left panels) and without stimulation (right panels). Images were captured after fixation, Nissl staining, and immunostaining for V5 tag. Enlarged views of the CytoTape in the top-row panels are shown in the two rows of rectangular panels below. The second-row and third-row panels show the timestamps and activity-dependent promoter-driven expression, respectively. The third-row panel shows statistical analysis of V5 signal relative change from baseline plotted against recovered time after plasmid transfection. Stimulation group, data are from n = 10 CytoTapes for *Fos* from 10 HEK cells from two cultures; n = 13 CytoTapes for *Hspa1a* from 13 HEK cells from two cultures. Control group, data are from n = 10 CytoTapes for *Fos* from 10 HEK cells from two cultures; n = 10 CytoTapes for *Hspa1a* from 10 HEK cells from two cultures. The dashed gray lines indicate the timestamps, marking the points after which the recovered time is accurate. Thick centerline, mean; darker boundary near the centerline, s.e.m.; lighter boundary, s.d.; thin light lines, individual CytoTape data. Scale bars, 10 μm.

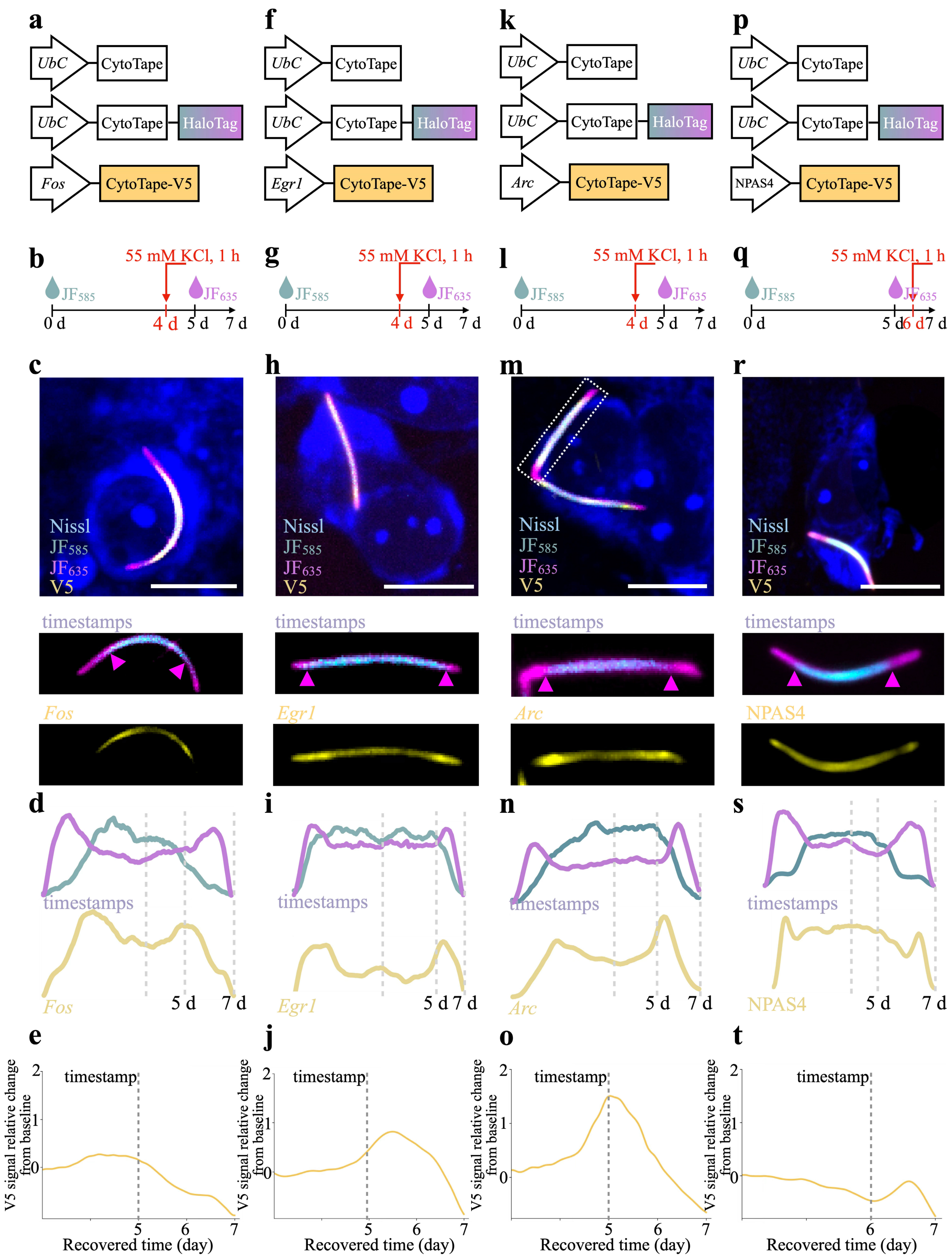
a
UbC
CytoTape
HaloTag
Fos
CytoTape-V5
f
Egr1
k
Arc
p
NPAS4
b
55 mM KCl, 1 h
JF585
JF635
0 d
4 d
5 d
7 d
g
l
q
6 d
c
h
m
r
Nissl
V5
timestamps
d
i
n
s
e
j
o
t
V5 signal relative change from baseline
timestamp
Recovered time (day)

Supplementary Fig. S17 CytoTape enables recording of *Fos*-, *Arc*-, and *Egr1*-promoter activities and NPAS4 activity in cultured neurons

(**a**), (**f**), (**k**), and (**p**) Schematic of the constructs transfected into cultured mouse hippocampal neurons and (**b**), (**g**), (**l**), and (**q**) the experimental timeline. The JF_{585} and JF_{635} dyes were added to neuron cultures on day 0 and day 5, respectively, and fixation was performed on day 7. Neurons were stimulated with 55 mM KCl for 1 h on day 4 for *Fos*, *Arc*, *Egr1* and day 6 for NPAS4. (**c**), (**h**), (**m**), and (**r**) Images of cultured neurons expressing the constructs shown in (**a**), (**f**), (**k**), and (**p**) following stimulation with 55 mM KCl for 1 h. Images were captured after fixation, Nissl staining, and immunostaining for V5 tags. Enlarged views of the CytoTape in the top-row panels are shown in the two rows of rectangular panels below. The second-row and third-row panels show the timestamps and activity-dependent promoter-driven gene expression, respectively. Scale bars, 10 μm. (**d**), (**i**), (**n**), and (**s**) The fluorescence line profiles of JF_{585}, JF_{635}, and V5 tags. (**e**) *Fos*, (**j**) *Egr1*, (**o**) *Arc*, and (**t**) NPAS4 signal relative change from baseline plotted against recovered time (interpolated with timestamps and fixation time) after calcium phosphate transfection. The dashed gray lines indicate the timestamps, marking the points after which the recovered time is accurate.

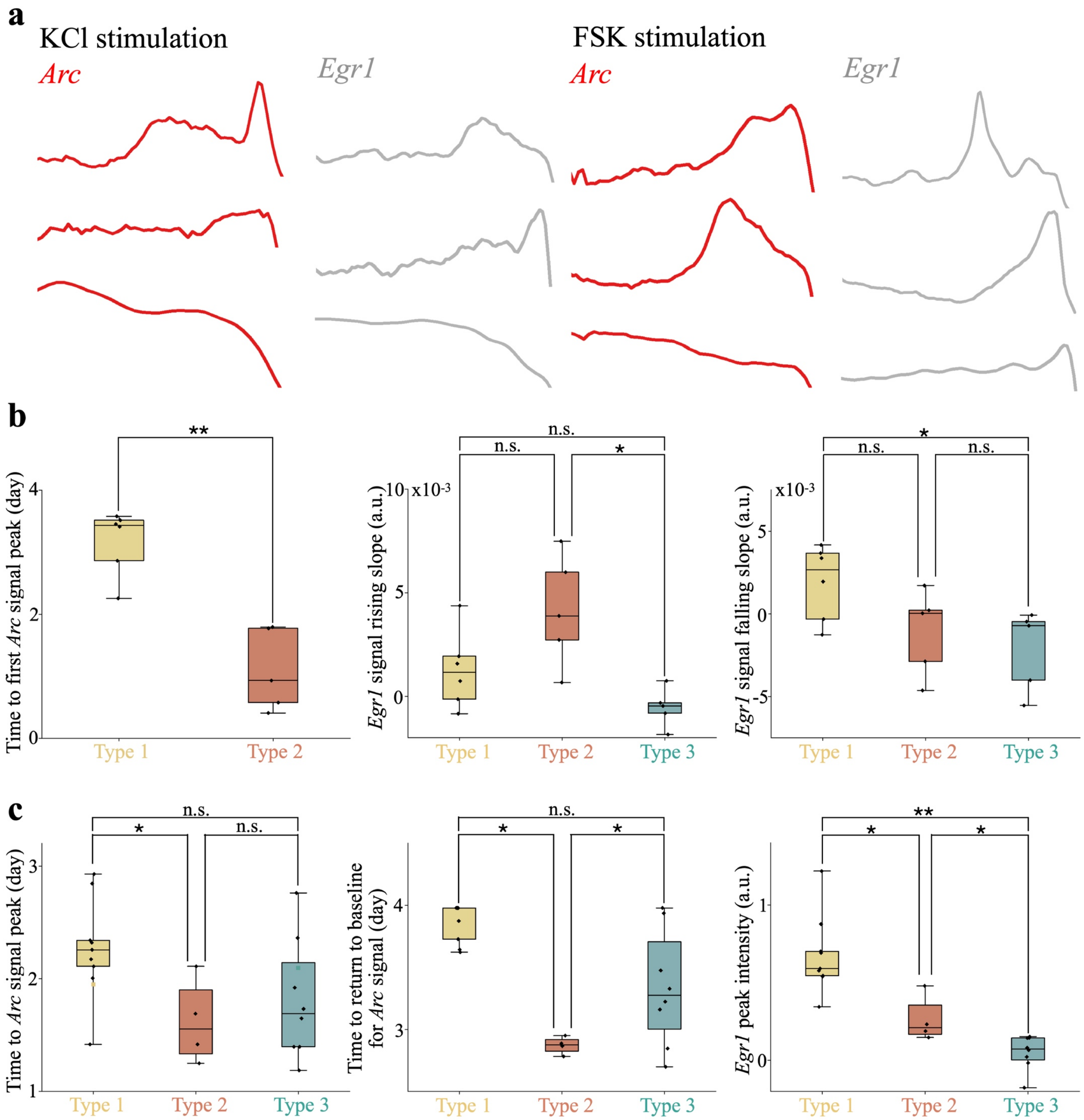

Supplementary Fig. S18 Temporal analysis of multiplexed recording of *Arc*- and *Egr1*-promoter-driven expression histories with CytoTape in cultured neurons

(**a**) Examples of single traces of *Arc* and *Egr1* signals under KCl (left panel) and FSK stimulation (right panel) recorded in separate CytoTapes without multiplexing. (**b**) Scatter plots showing the time between stimulation point (day 17) and the first peak maximum amplitude point of *Arc* signal (left panel, only Type 1 and Type 2 data are shown as Type 3 has no peak), the rising slope of the *Egr1* signal (middle panel), and the falling slope of the *Egr1* signal (right panel) across three neuronal subtypes under

KCl stimulation. Data are from n = 16 CytoTapes from 16 neurons from three cultures. (**c**) Scatter plots showing the time between stimulation point (day 17) and the peak maximum amplitude point of *Arc* signal (left panel), the time between stimulation point (day 17) and peaks' returning to baseline point for *Arc* signal (middle panel), and the peak maximum intensity of the *Egr1* signal (right panel) across three neuronal subtypes under FSK stimulation. Data are from n = 21 CytoTapes from 21 neurons from three cultures. Middle line in box plot, median; box boundary, interquartile range; whiskers, minimum and maximum; black dots, individual data points. *, $P < 0.05$; **, $P < 0.01$; n.s., not significant; Kruskal–Wallis analysis of variance followed by Dunn's post hoc tests. See **Supplementary Table S11** for details of statistical analysis.

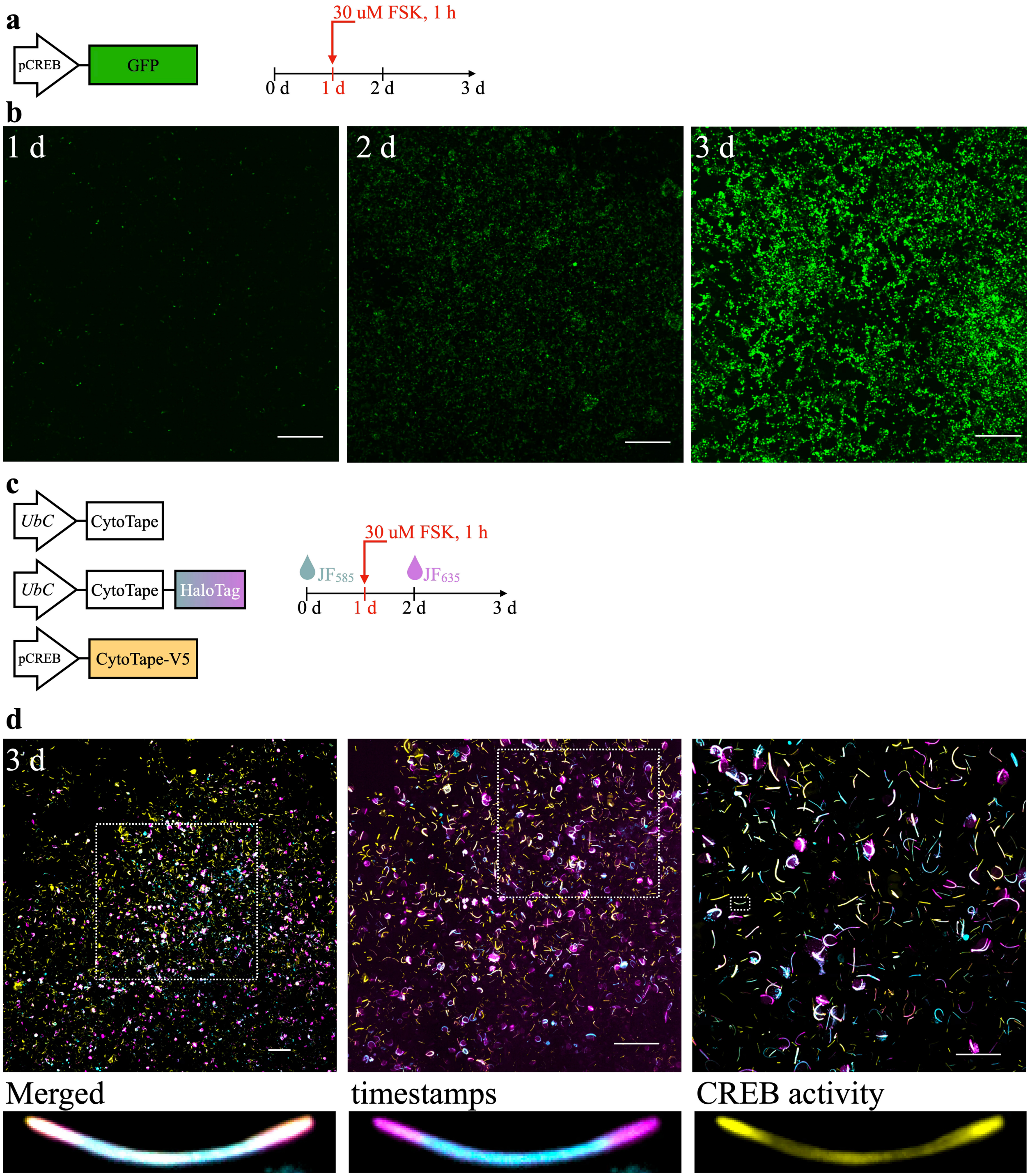

Supplementary Fig. S19 Comparison of CREB activity following forskolin stimulation recorded by live GFP imaging and CytoTape in HEK cells

(**a**) Schematic of the constructs transfected into HEK cells (left panel) and time points of stimulation (30 µM FSK for 1 h) (right panel). (**b**) Confocal images of live imaging of GFP driven by the pCREB promoter. Scale bar, 100 µm. (**c**) Schematic of the constructs

transfected into HEK cells (left panel) and time points of JF_{585} and JF_{635} addition and fixation and stimulation (30 μM FSK for 1 h) (right panel). The JF_{585} and JF_{635} dyes were used for labeling time within the CytoTape. (**d**) Low magnification images of CytoTape in HEK cells, which is taken after fixation on day 3, and immunostaining against the V5 tag. The objective lenses used for the left panel, middle panel, and right panel were 10×, 20×, and 40×, respectively. Some fibers in these low-magnification images do not display clear timestamp transitions or pCREB signals because the optimal image contrast to visualize these fluorescence features in each color channel varies across individual cells.

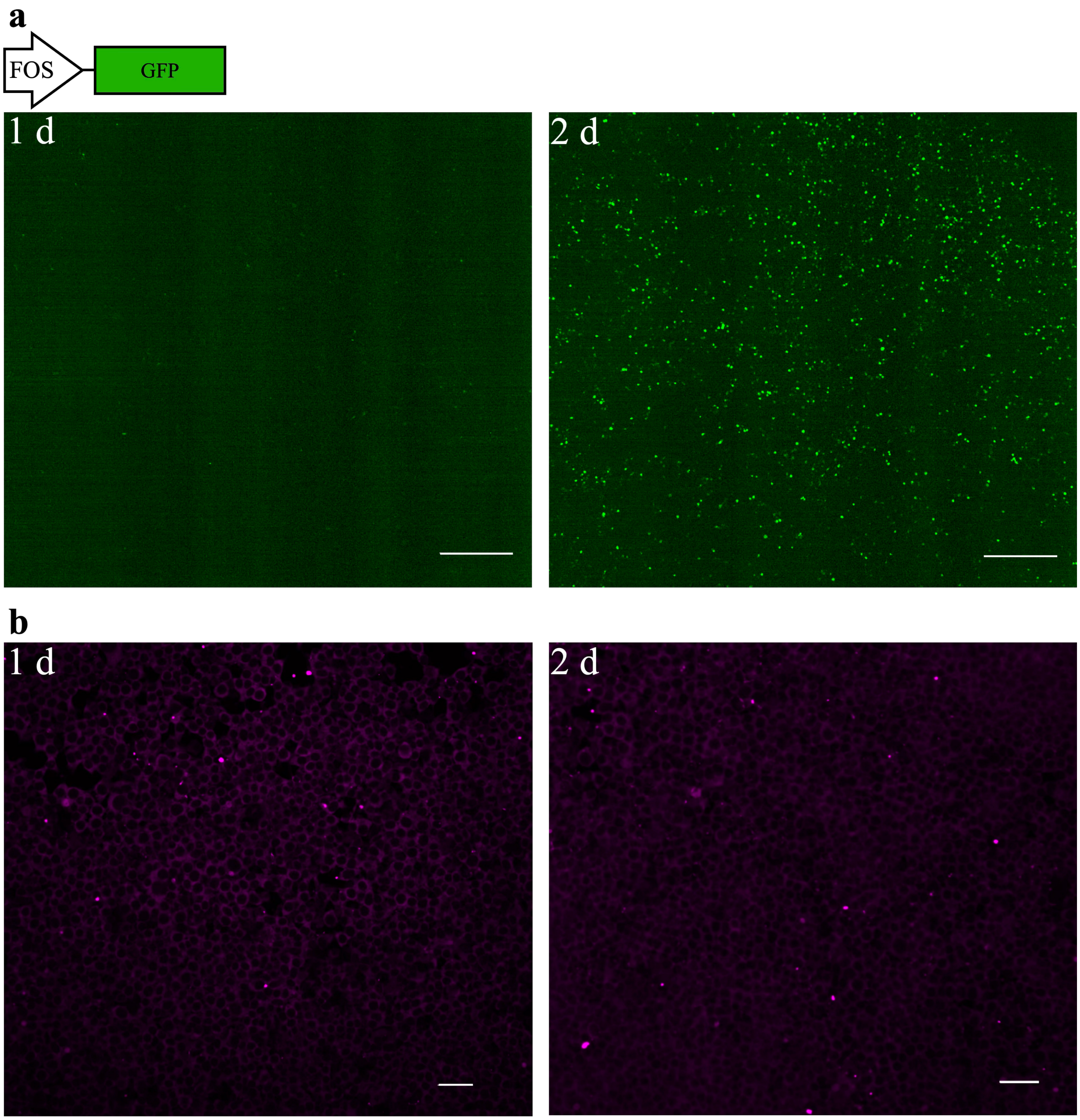

Supplementary Fig. S20 Live-cell GFP reporter imaging exhibits baseline drift over time unrelated to the biological activity of interest

(**a**) Confocal images of live imaging of GFP driven by the *F*-RAM promoter. Scale bar, 100 μm. (**b**) Confocal images of FOS protein immunostaining in cells fixed at 24 hours (left panel) and 48 hours (right panel) after plasmid transfection. Scale bar, 50 μm.

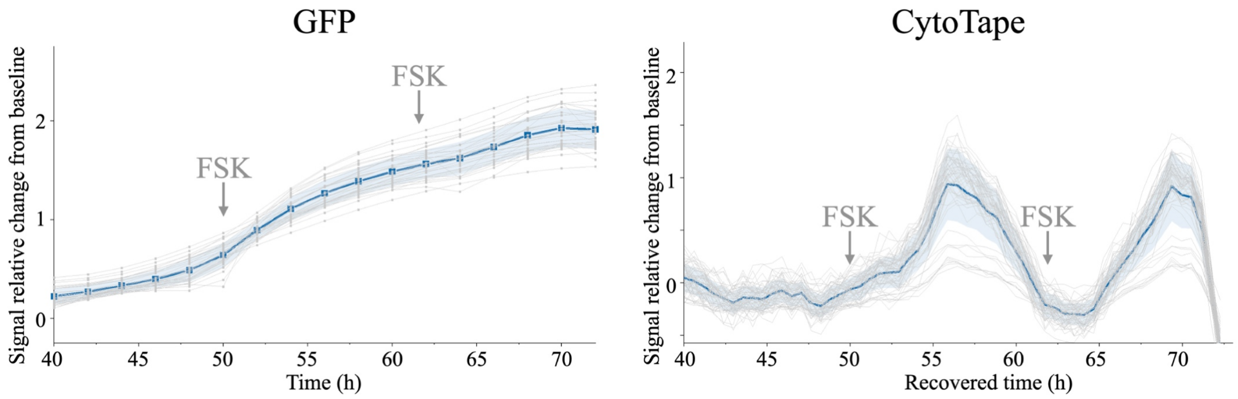

Supplementary Fig. S21 CytoTape captures two sequential stimulations of CREB activity undetectable by time-lapse GFP imaging under low dose forskolin treatments in HEK cells

10 μM FSK was added to the culture at 50 and 62 hours after plasmid transfection, with each stimulation lasting 1 hour. GFP time-lapse images were captured every 2 hours. GFP imaging was performed in 31 HEK cells, and CytoTape recording in 60 HEK cells.

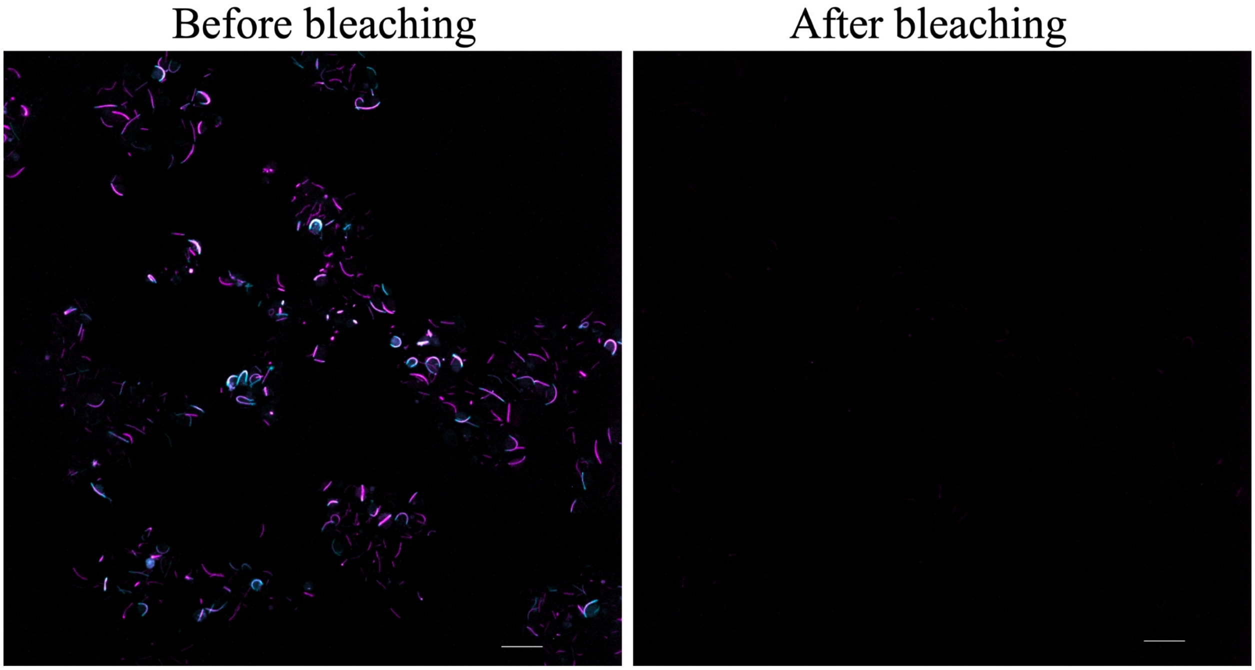

Supplementary Fig. S22 Confocal images of JF_{585} and JF_{635} on CytoTapes in HEK cells before and after photobleaching

The same field of view was imaged under identical conditions before (left) and after (right) photobleaching of the JF dyes by white LED light for 50 minutes. The image contrast is identical between the two images. Scale bar, 50 μm.

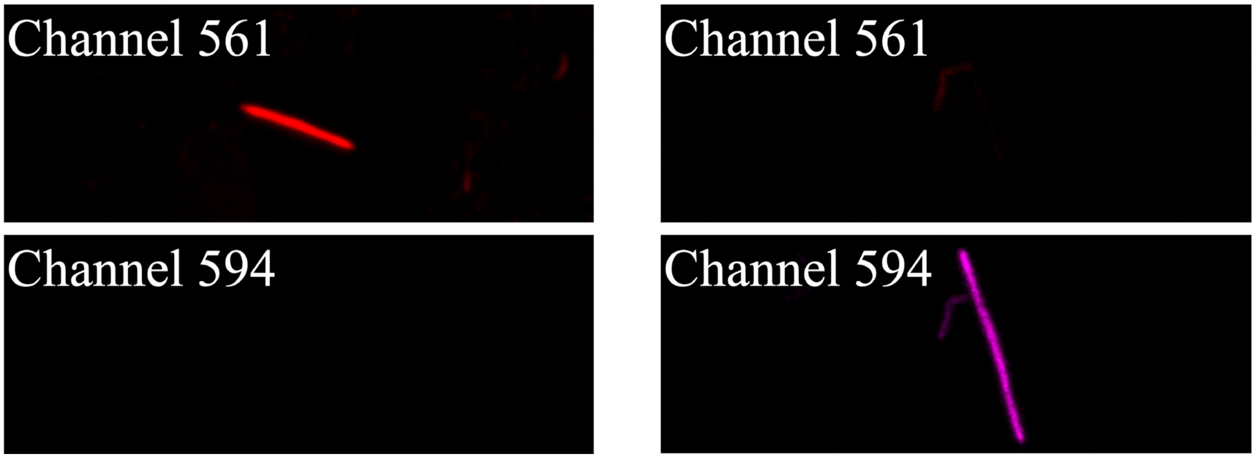

Supplementary Fig. S23 No optical crosstalk was observed between the Alexa Fluor 546 dye in the 561 nm channel and the Alexa Fluor 594 dye in the 594 nm channel under the imaging conditions used in this work

Two fields of view (left and right) were imaged under confocal microscopy. The imaging conditions and image contrast for each channel are identical between the two fields of view.

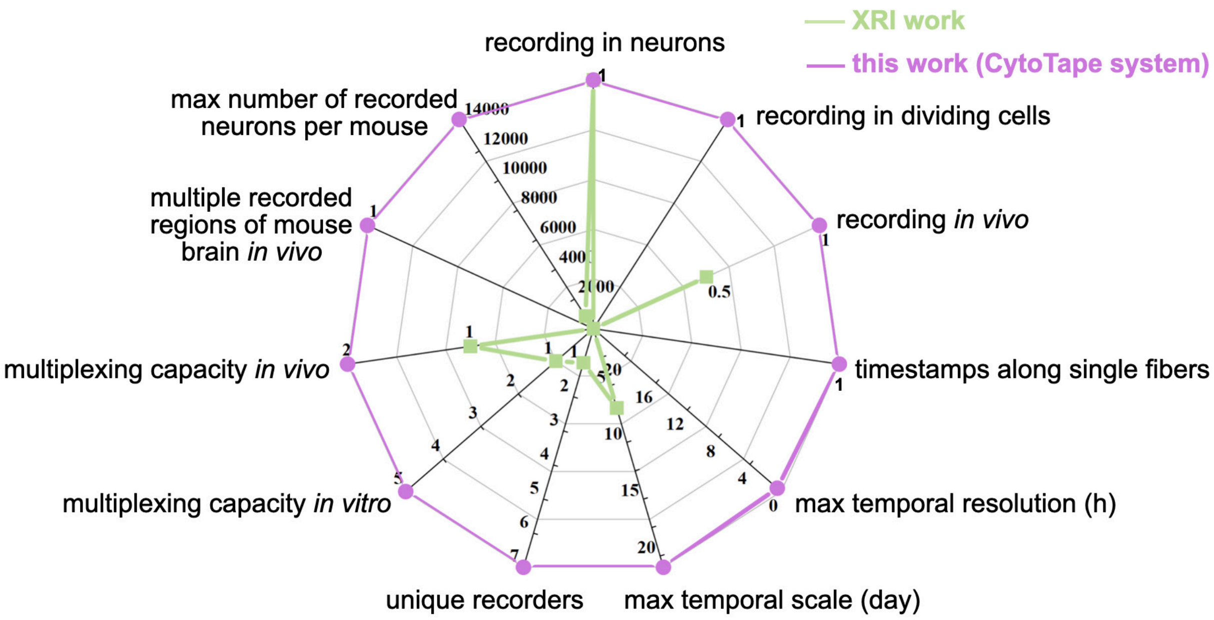

Supplementary Fig. S24 Comparison of CytoTape and XRI systems

Radar plot illustrating major capabilities of the XRI system (green) and CytoTape system (purple). Axes represent recording performance metrics, including (clockwise from top) recording in neurons, recording in dividing cells, recording in vivo, timestamps along a single fiber, maximum temporal resolution, maximum temporal scale, number of unique recorders, multiplexing capacity in vitro and in vivo, multiple recorded brain regions in vivo, and maximum number of recorded neurons per mouse. Radial grids denote quantitative or categorical scales for each feature. For “recording in neurons,” “recording in dividing cells,” “recording in vivo,” and “timestamps along a single fiber,” a value of 0 indicates no physiological signal recording capability or single-cell time axis, while a value of 1 indicates the presence of such capability. For “multiple recorded brain regions *in vivo*”, a value of 0 indicates that the system lacks this capability, whereas a value of 1 indicates that the capability is present. A value of 0.5 for “recording in vivo” denotes the system preserves temporal information but lacks an explicit single-cell time axis. All the XRI results are obtained from Nat. Biotechnol. 41, 640–651 (2023).

Supplementary Video S1 Tissue-wide computational segmentation of CytoTape-vivo fibers and cell morphology for in vivo applications

The video sequentially displays: (1) cell morphology from Nissl staining under confocal microscopy; (2) cell segmentation via the Tape Reader pipeline; (3) CytoTape-vivo fibers from structural monomer immunofluorescence under confocal microscopy; (4) fiber segmentation via the Tape Reader pipeline; (5) centerlines of segmented fibers identified by the Tape Reader pipeline, for downstream extraction of recorded signals; and (6) a zoomed-out overlay of segmented cell morphology and fiber centerlines in the mouse brain tissue.

Supplementary Table S1 Sequences of protein motifs used in this study

Motif name	Amino acid sequence	Ref.
1POK (E239Y)	MIDYTAAGFTLLQGAHLYAPEDRGICDVLVANGKIIAVASNI<br>PSDIVPNCTVVDLSGQILCPGFIDQHVHLIGGGGEAGPTTR<br>TPEVALSRLTEAGVTSVVGLLGTDSISRHPESLLAKTRALN<br>EEGISAWMLTGAYHVPSRTITGSVEKDVAIIDRVIGVKCAIS<br>DHRSAAPDVYHLANMAAESRVGGLLGGKPGVTVFHMGDS<br>KKALQPIYDLLENCDVPISKLLPTHVNRNVPLFYQALEFARK<br>GGTIDITSSIDEPVAPAEGIARAVQAGIPLARVTLSSDGNGS<br>QPFFDDEGNLTHIGVAGFETLLETVQVLVKDYDFSISDALR<br>PLTSSVAGFLNLTGKGEILPGNDADLLVMTPELRIEQVYAR<br>GKLMVKDGKACVKGTFETA	[8]
1POK (E239Y, E149I)	MIDYTAAGFTLLQGAHLYAPEDRGICDVLVANGKIIAVASNI<br>PSDIVPNCTVVDLSGQILCPGFIDQHVHLIGGGGEAGPTTR<br>TPEVALSRLTEAGVTSVVGLLGTDSISRHPESLLAKTRALN<br>EEGISAWMLTGAYHVPSRTITGSVIKDVAIIDRVIGVKCAISD<br>HRSAAPDVYHLANMAAESRVGGLLGGKPGVTVFHMGDSK<br>KALQPIYDLLENCDVPISKLLPTHVNRNVPLFYQALEFARKG<br>GTIDITSSIDEPVAPAEGIARAVQAGIPLARVTLSSDGNGSQ<br>PFFDDEGNLTHIGVAGFETLLETVQVLVKDYDFSISDALRPL<br>TSSVAGFLNLTGKGEILPGNDADLLVMTPELRIEQVYARGK<br>LMVKDGKACVKGTFETA	Mutated in this work
1POK (E239Y, L349K)	MIDYTAAGFTLLQGAHLYAPEDRGICDVLVANGKIIAVASNI<br>PSDIVPNCTVVDLSGQILCPGFIDQHVHLIGGGGEAGPTTR<br>TPEVALSRLTEAGVTSVVGLLGTDSISRHPESLLAKTRALN<br>EEGISAWMLTGAYHVPSRTITGSVEKDVAIIDRVIGVKCAIS<br>DHRSAAPDVYHLANMAAESRVGGLLGGKPGVTVFHMGDS<br>KKALQPIYDLLENCDVPISKLLPTHVNRNVPLFYQALEFARK<br>GGTIDITSSIDEPVAPAEGIARAVQAGIPLARVTLSSDGNGS<br>QPFFDDEGNLTHIGVAGFETLLETVQVLVKDYDFSISDALR<br>PLTSSVAGFLNLTGKGEIKPGNDADLLVMTPELRIEQVYAR<br>GKLMVKDGKACVKGTFETA	Mutated in this work
Maltose binding protein from *E. coli* (MBP, 366 AAs)	KIEEGKLVIWINGDKGYNGLAEVGKKFEKDTGIKVTVEHPD<br>KLEEKFPQVAATGDGPDIIFWAHDRFGGYAQSGLLAEITPD<br>KAFQDKLYPFTWDAVRYNGKLIAYPIAVEALSLIYNKDLLPN<br>PPKTWEEIPALDKELKAKGKSALMFNLQEPYFTWPLIAADG<br>GYAFKYENGKYDIKDVGVDNAGAKAGLTFLVDLIKNKHMN<br>ADTDYSIAEAAFNKGETAMTINGPWAWSNIDTSKVNYGVT<br>VLPTFKGQPSKPFVGVLSAGINAASPNKELAKEFLENYLLT<br>DEGLEAVNKDKPLGAVALKSYEEELAKDPRIAATMENAQK<br>GEIMPNIPQMSAFWYAVRTAVINAASGRQTVDEALKDAQT	[102]
Maltose binding protein from *K. pneumonia*	MKTEEGKLVIWINGDKGYNGLAEVGKKFEKDTGIKVTVEHP<br>DKLEEKFPQVAATGDGPDIIFWAHDRFGGYAQSGLLAEITP<br>DKAFQDKLYPFTWDAVRYNGKLIAYPIAVEALSLIYNKDLLP<br>NPPKTWEEIPALDKELKAKGKSALMFNLQEPYFTWPLIAAD	BLAST

e (*Kp*MBP, 246 AAs)	GGYAFKYENGKYDIKDVGVDNAGAKAGLTFLVDLIKNKHM NADTDYSIAEAAFNKGETAMTINGPWAWSNICLLYTSDAAD	
Maltose binding protein from *P. stutzeri* (*Ps*MBP, 453 AAs)	MAYFVRLRATAYAPHLRPPPPGGGGCRWHGTRRGWNDC QSQRPAAGGTKNKKPRGTRSMNTKFWCLATIGLAATFSLP LPALAAIEEGKLVVWINGDKGYKGLAEVGKRFTAETGIPVE VAHPDSATDKFQQAAATGNGPDIFIWAHDRIGEWAKSGLL TPVTPSAETKSGIADFSWQAVTYDNKLWGYPISVETIGLIYN KALVDTPPKSFDDVLALNETLAPQGKRAILWDYNNTYFTW PLLSAKGGYVFEQTDGGYNVKSTGVNNAGAKAGAKVLRE LIDKGVMPKGADYSVAEAAFNKGDSAMMISGPWAWSNIEK SGIDFGVAPIPAIDGEAGKPFVGVAAALLNAASPNKDLAVE FLENYLLEVDGLKTVNADVPLGAVANTAYMEELSSNPHIKA TFENAQMGQPMPNVPEMGAFWSSMAAALTNITSGRQDV DAALDDAAKRITR	BLAST
N-terminal truncated maltose binding protein from *E. coli* (dMBP, 362 AAs)	GKLVIWINGDKGYNGLAEVGKKFEKDTGIKVTVEHPDKLEE KFPQVAATGDGPDIIFWAHDRFGGYAQSGLLAEITPDKAFQ DKLYPFTWDAVRYNGKLIAYPIAVEALSLIYNKDLLPNPPKT WEEIPALDKELKAKGKSALMFNLQEPYFTWPLIAADGGYAF KYENGKYDIKDVGVDNAGAKAGLTFLVDLIKNKHMNADTD YSIAEAAFNKGETAMTINGPWAWSNIDTSKVNYGVTVLPTF KGQPSKPFVGVLSAGINAASPNKELAKEFLENYLLTDEGLE AVNKDKPLGAVALKSYEEELAKDPRIAATMENAQKGEIMP NIPQMSAFWYAVRTAVINAASGRQTVDEALKDAQT	Mutated from MBP in this work
Halotag (Halotag7)	MAEIGTGFPFDPHYVEVLGERMHYVDVGPRDGTPVLFLHG NPTSSYVWRNIIPHVAPTHRCIAPDLIGMGKSDKPDLGYFF DDHVRFMDAFIEALGLEEVVLVIHDWGSALGFHWAKRNPE RVKGIAFMEFIRPIPTWDEWPEFARETFQAFRTTDVGRKLII DQNVFIEGTLPMGVVRPLTEVEMDHYREPFLNPVDREPLW RFPNELPIAGEPANIVALVEEYMDWLHQSPVPKLLFWGTP GVLIPPAEAARLAKSLPNCKAVDIGPGLNLLQEDNPDLIGSE IARWLSTLEISG	[103]
MreB from *B. subtilis*	MFGIGARDLGIDLGTANTLVFVKGKGIVVREPSVVALQTDT KSIVAVGNDAKNMIGRTPGNVVALRPMKDGVIADYETTAT MMKYYINQAIKNKGMFTRKPYVMVCVPSGITAVEERAVIDA TRQAGARDAYPIEEPFAAAIGANLPVWEPTGSMVVDIGGG TTEVAIISLGGIVTSQSIRVAGDEMDDAIINYIRKTYNLMIGD RTAEAIKMEIGSAEAPEESDNMEIRGRDLLTGLPKTIEITGK EISNALRDTVSTIVEAVKSTLEKTPPELAADIMDRGIVLTGG GALLRNLDKVISEETKMPVLIAEDPLDCVAIGTGKALEHIHLF KGK	[16]
MreBCD proteins from *E. coli*	MLKKFRGMFSNDLSIDLGTANTLIYVKGQGIVLNEPSVVAIR QDRAGSPKSVAAVGHDAKQMLGRTPGNIAAIRPMKDGVIA DFFVTEKMLQHFIKQVHSNSFMRPSPRVLVCVPVGATQVE RRAIRESAQGAGAREVFLIEEPMAAAIGAGLPVSEATGSMV VDIGGGTTEVAVISLNGVVYSSSVRIGGDRFDECIINYVRRN YGSLIGEATAERIKHEIGSAYPGDEVREIEVRGRNLAEGVP	[15]

	RGFTLNSNEILEALQEPLTGIVSAVMVALEQCPPELASDISERGMVLTGGGALLRNLDRLLMEETGIPVVVAEDPLTCVARGGGKALEMIDMHGGDLFSEE	
Actin-related protein MreB from *E. coli*	MKLAPYILELLTSVNRTNGTADLLVPLLRELAKGRPVSRTTLAGILDWPAERVAAVLEQATSTEYDKDGNIIGYGLTLRETSYVFEIDDRRLYAWCALDTLIFPALIGRTARVSSHCAATGAPVSLTVSPSEIQAVEPAGMAVSLVLPQEAADVRQSFCCHVHFFASVPTAEDWASKHQGLEGLAIVSVHEAFGLGQEFNRHLLQTMSSRTP	[14]
PilAN from *Geobacter*	MANYPHTPTQAAKRRKETLMLQKLRNRKGFTLIELLIVVAIIGILAAIAIPQFSAYRVKAYNSAASSDLRNLKTALESAFADDQTYPPES	[13]
PilAC from *Geobacter*	MKKIITIVAMLLAMQGIAIAAGKIPTTTMGGKDFTFKPSTNVSVSYFTTNGATSTAGTVNTDYAVNTKNSSGNRVFTSTNNTSNIWYIENDAWKGKAVSDSDVTALGTGDVGKSDFSGTEWKSQ	[13]
AvECN from *P. calidifontis*	MNGGKLALLMVTLAAMGTLVLPSTTSLFLGQHMWYNISGTGNNLPCEKCHADVFAEFKNNPGAHKTIGGGTDTVEHIRAACGECHRTSVVGTFASGDGTSATPGQEAHAAATIACMACHEFGPNGNAPYSGAPVAGGFDNVTTDTASSPYNYDNGDTTYGTKEAHQTFIERAVEDKTLIDSNEACIACHTYVPVKINWTHKVSLEFNCTYEYNTGTSGVTTHYNVTNWTVNGTRYTTVFGNTTGNGSVNDASNWPGWYPYSW	[12]
ATT1	MQDYSAAGHTLLKGAHLYAPEDRGICDVLIANGKIISLASDIPSDIVPDCTVIDLTGQIMCPGFMDQHTHMTGGGGEAGDQTRTPQAALSRLTAAGVTSAIGLLGTDATSRHPEALLAKTRALNREGITAYMLTGSYHVPSRTITGDVEKDVATLARVIGVKCAISDHRSSSPNVRILATMAADARVGGLIGGKPGVTHFHMGSSRKLLLPIFDLLQNCDVPLGKLLPTHTNRNEPLFYQALEFARRGGTIDITTGIDHPVAPAQGIARAREAGIPLARVTLSSDGNGSQPIFDEQGNIVQIGIAGFDALAETIQKLVRSYDFTLANALRPLTSSVAGFLNLEGKGEIQPGFDADLLVLTPQLRVEHVLAKGKLMVKDGKACVRGTYEQA	Generated in this work
ATT2	MVDFSAAGNTLLQGGHLYAPEDRGICDILIANGKIIAVTSNIPADIVAHCTVVDLTGMIACPGFIDQHVHVTGGGGEAGPQQRTDRVALSRLTAAGVTSVIGLLGTDSISRHPEALLAQCRELNKEGITAWMLTGAYHVPSRTILGSVEYDVAIIDRAIGVKCAISDHRSSSPDVKMLADQAADSRVGGLLGGKPGVSCFHMGNDRRSLQPLYDLLQNCDVPAGKLLPTHVNRNTPLYYQALEFARRGGTIDITSSIDEPVTPSEGISWAQEAGIPLARICLSSDGNASVPQYDEEGCLTEISIAGFESLAETVQLLVRTRNFSISDALRPFTSSVAGFLDLEGKGQILPGHDADILVMTPEMRIEQVLARGELMVRDGRACVWGDYEDA	Generated in this work
ATT3	MIDYSAAGHILLQGAHLYAPDARGICDVLLADGKIIAVASNIPADLVPNCTVLDATGMNLCPGFVDQHVHVIGGGGEAGPHTRTPEAALSRLTEAGVTTVVGLLGTDSISRHPEALLAKCRAF	Generated in this work

	NREGISAWMLTGAYHVPSRTITGNVEKDVAEIALVIGVKCCI SDHRSSSPDTYLLGTMAADARVGGLLGGKPGVSVFHMGS SRWGLQPIFDLLDNCDVPMGKLLPTHVNRNTPLFYEALEF ARRGGTIDITTGIHKPVAPGEGIARAVEAGIPLARITLSSDGN GSQPEYDDEGNIIDMGIAGFESLLETVQTLVRDFDFSISDAL RPGTSSVAGFLNLQGKGQILPGYDADILVLTPQLRLEEVLA KGKVMVRDGKACVKGDYEVA	
ATT4	MVDFSSAGYTLLQGAHLYAPEAWGICDVLLSNGKIIAVTANI PADIVPHCTVIDLTGQILCPGFFDQHVHVTGGGGEAGNVS RTPEVDLSRLTTAGVTTTLGLLGTDAISRHPEALLAMTREL NELGISSWMLTGTYHVPSKTVTGNVEKDVALISKAVGVKC AVSDHRSAAPETRIVGNMAAQAYVGGLLGGKAGVSLFHM GSDRKGLQPFYDTLQNCDVPMGKLLPTHVNQNIPLYYQAL EWARRGGVIDITSSIDKPIAPSDAIARAVEAGIPLARLTLASD GNGSQPLFDDQGNLVHIGVSGFEWLLETVQELVRDRNYTI SDALRPLTSAVAGFLELQGKGEIEPGFDADLLIMTAQLRLE QVYARGRLVVRDGRACVRGTREDA	Generate d in this work
ATT5	MIDYTHAGFTLLQGAHLYAPEDRGICDVLLANGRIIAVSSNI PADIVPDCTVLDATGLILCPGFIDQHQHVIGGGGEAGPNTR TPEVALSRFTTAGVTIVIGLLGTDALSRHPEALLAKCRALDE EGISAWMLTGSYHVPSRTITGSVEKDVAIIDRVIGVKCAVSD HRSSSPDTYELATAAADSRVGGLLGGKPGCSVFHMGNDK KGLQPLFDLLENCNVPIGKLLPTHVNRNTPLFYEALEFARR GGTIDLTSSIDQPIAPAEGIALAVEAGIPLSKVTLSSDGNGS QPHYDQQGDLTQISVAGFETLLETVKELVRDYDFSIATALR PFTSSVAGFLDLHGKGEILTGFDADLLVMSPELRVLQVLAR GKNMVRDGKACVRGEYEDA	Generate d in this work
HA (HA tag)	YPYDVPDYA	
V5 (V5 tag)	GKPIPNPLLGLDST	
Etag (E tag)	GAPVPYPDPLEPR	
OLLAS (OLLAS tag)	SGFANELGPRLMGK	
FLAG (FLAG tag)	DYKDDDDK	
Linker2	GG	
Linker3	GSG	
Linker5	GGGSG	
Linker6	GGSGGT	
Linker25	GGSGGTGGSGGTGGSGGTGGSGGTG	

Supplementary Table S2 Constructs of signal monomer used in cultured neurons in this study

**Construct** (promoters are underlined)	Reference for promoter sequence
Fos-1POK(E239Y, L349K)-dMBP-V5	[1]
SARE_ArcMin-1POK(E239Y, L349K)-dMBP-V5	[42]
6x*CRE*_CMVMin-1POK(E239Y, L349K)-dMBP-V5	[45]
Egr1-1POK(E239Y, L349K)-dMBP-V5	[34]
F-RAM-1POK(E239Y, L349K)-dMBP-V5	[104]
N-RAM-1POK(E239Y, L349K)-dMBP-V5	[104]
Egr1-1POK(E239Y, L349K)-dMBP-OLLAS	[34]
SARE_ArcMin-1POK(E239Y, L349K)-dMBP-OLLAS	[42]

Supplementary Table S3 Constructs of signal monomer used in HEK293T in this study

**Construct** (promoters are underlined)	Reference for promoter sequence
Fos-1POK (E239Y, L349K)-dMBP-V5	[1]
SARE_ArcMin-1POK(E239Y, L349K)-dMBP-V5	[42]
6x*CRE*_CMVMin-1POK(E239Y, L349K)-dMBP-V5	[45]
Egr1-1POK(E239Y, L349K)-dMBP-Etag	[34]
F-RAM-1POK(E239Y, L349K)-dMBP-OLLAS	[104]
N-RAM-1POK(E239Y, L349K)-dMBP-FLAG	[104]
Hspa1a-1POK(E239Y, L349K)-dMBP-V5	[35]
Fos-1POK(E239Y, L349K)-dMBP-HA	[1]

Supplementary Table S4 Constructs of signal monomer used in HeLa in this study

**Construct** (promoters are underlined)	Reference for promoter sequence
Hspa1a-1POK(E239Y, L349K)-dMBP-V5	[35]

Supplementary Table S5 Constructs of timestamp monomer used in cell culture in this study

**Construct** (promoters are underlined)	Reference for HaloTag sequence
UbC-1POK(E239Y, L349K)-dMBP-HA-gsg-HaloTag	[103]

Supplementary Table S6 Constructs used in the mouse brain *in vivo* in this study

**Construct** (promoters are underlined)	Reference for promoter sequence
UbC-1POK(E239Y, L349K)-linker6-MBP-HA	[1]
Fos-1POK(E239Y, L349K)-dMBP-V5	[1]
hSyn-rtTA	Tet-On 3G tetracycline-inducible expression systems (TaKaRa Cat# 631165)
TRE-1POK(E239Y)-linker25-FLAG-linker3-MBP	Tet-On 3G tetracycline-inducible expression systems (TaKaRa Cat# 631165)

Supplementary Table S7 Constructs of structural monomer tested in the mouse brain *in vivo* in this study

Name	**Construct** (promoters are underlined)
CytoTape-vivo	*UbC*-1POK(E239Y, L349K)-linker6-MBP-HA
CytoTape variant 1 (linker10)	*UbC*-1POK(E239Y, L349K)-linker10-MBP-HA
CytoTape variant 2 (linker12)	*UbC*-1POK(E239Y, L349K)-linker12-MBP-HA
CytoTape variant 3 (linker18)	*UbC*-1POK(E239Y, L349K)-linker18-MBP-HA

Supplementary Table S8 Constructs of structural monomer designs tested in this study

**Name**	**Construct** (promoters are underlined)	**Morphology in cultured mouse neurons (unless noted otherwise)**
A1-1	*UbC*-ATT1-Liner25-HA-Linker3-MBP	Short fibers
A1-2	*UbC*-ATT2-Liner25-HA-Linker3-MBP	Short fibers
A1-3	*UbC*-ATT3-Liner25-HA-Linker3-MBP	Short fibers
A1-4	*UbC*-ATT4-Liner25-HA-Linker3-MBP	Short fibers
A1-5	*UbC*-ATT5-Liner25-HA-Linker3-MBP	Short fibers
A2-1	*UbC*-1POK(E239Y, E149I)-Linker25-HA-Linker3-MBP	Fibers
A2-2	*UbC*-1POK(E239Y, L349K)-Linker25-HA-Linker3-MBP	Fibers
A3-1	*UbC*-mhYFP-Linker5-B. subtilis MreB(mrebbs)	Puncta
A3-2	*UbC*-mhYFP-Linker5-E. coli(mrebec)	Intertwined fibers
A3-3	*UbC*-mGFP-Linker5-EcMReB	Intertwined fibers
A3-4	*UbC*-PilAN-Linker3-HA-Linker3-PilAC	Puncta
A3-5	*UbC*-AvECN-Linker3-HA	Puncta
B-1	*UbC*-1POK(E239Y)-Linker2-MBP-HA	Fibers
C-1	*UbC*-1POK(E239Y)-Liner25-HA-Linker3-*Kp*MBP	Fibers
C-2	*UbC*-1POK(E239Y)-Liner25-HA-Linker3-*Ps*MBP	Puncta
C-3	*UbC*-1POK-Liner25-HA-Linker3-MBP-Linker3-MBP	Puncta
C-4	*UbC*-1POK(E239Y)-Linker2-dMBP-HA	Fibers
C-5	*UbC*- mGFP-P2A-1POK(E239Y)-Liner25-HA-Linker3-MBP	Fibers
C-6	*UbC*-1POK(E239Y)-dMBP-HA	Fibers
CytoTape	*UbC*-1POK(E239Y, L349K)-dMBP-HA	Fibers, in cultured mouse neurons; fibers, in neurons in mouse brain *in vivo*
CytoTape variant 1 (linker10)	*UbC*-1POK(E239Y, L349K)-Linker10-MBP-HA	Fibers, in cultured mouse neurons; fibers, in neurons in mouse brain *in vivo*

CytoTape variant 2 (linker12)	*UbC*-1POK(E239Y, L349K)-Linker12-MBP-HA	Fibers, in cultured mouse neurons; fibers, in neurons in mouse brain *in vivo*
CytoTape variant 3 (linker18)	*UbC*-1POK(E239Y, L349K)-Linker18-MBP-HA	Fibers, in cultured mouse neurons; fibers, in neurons in mouse brain *in vivo*
CytoTape-vivo	*UbC*-1POK(E239Y, L349K)-Linker6-MBP-HA	Fibers, in cultured mouse neurons; fibers, in neurons in mouse brain *in vivo*

Supplementary Table S9 Comparison of XRI, iPAK4, and CytoTape in multiple cell types in cell culture and *in vivo*

**Tech Specs**	**XRI**[1]	**iPAK4**[2]	**CytoTape** (this work)
Morphology in cultured neurons	fiber	fiber	fiber
Morphology in HEK cells	puncta and fiber	fiber	fiber
Morphology in HeLa cells	intertwined fiber	not tested	fiber
Morphology in cultured glial cells	not tested	not tested	fiber
Protruding outward and significantly deforming the cell membrane when the fiber is longer than the cell	not observed	observed	not observed
Time recovery method in cell culture	Time calibration via tamoxifen-induced Cre/FLEX system	Timestamps via HaloTag and dye switches	Timestamps via HaloTag and dye switches
Time recovery method *in vivo*	No time recovery; validated the tamoxifen-	not tested	TRE/Dox (Tet-On) system

	induced Cre/FLEX system		
Shortest resolvable timestamp interval tested in cell culture (hour)	No timestamp; with time calibration, the precision is ~24 h	1	0.5
Longest recording duration reported in cultured neurons (day)	7	3	21
Longest recording duration reported in HEK cells (day)	not tested	1	5
Longest recording duration reported in HeLa cells (day)	not tested	not tested	4
Number of developed recorders for distinct cell physiological activities	1 (*Fos* transcriptional activity)	1 (*Fos* transcriptional activity)	7 (*Fos*, *Arc*, *Egr1*, and *Hspa1a* transcriptional activities; CREB, NPAS4, and FOS protein activities)
Reported number of simultaneously recorded	1	1	5

distinct cell physiological activities			

Supplementary Table S10 List of reagents and resources used in this study

Supplementary Table S11 Details of statistical analysis

Supplementary references

1 Linghu, C. *et al.* Recording of cellular physiological histories along optically readable self-assembling protein chains. *Nat. Biotechnol.* **41**, 640-651 (2023).

2 Lin, D. *et al.* Time-tagged ticker tapes for intracellular recordings. *Nat. Biotechnol.* **41**, 631-639 (2023).

3 Yang, S. *et al.* Membrane curvature governs the distribution of Piezo1 in live cells. *Nat. Commun.* **13**, 7467 (2022).

4 Sinha, B. *et al.* Cells Respond to Mechanical Stress by Rapid Disassembly of Caveolae. *Cell* **144**, 402-413 (2011).

5 Baskaran, Y. *et al.* An in cellulo-derived structure of PAK4 in complex with its inhibitor Inka1. *Nat. Commun.* **6**, 8681 (2015).

6 Li, T. L. *et al.* Engineering a Genetically Encoded Magnetic Protein Crystal. *Nano Lett.* **19**, 6955-6963 (2019).

7 Schönherr, R. *et al.* A streamlined approach to structure elucidation using in cellulo crystallized recombinant proteins, InCellCryst. *Nat. Commun.* **15**, 1709 (2024).

8 Garcia-Seisdedos, H., Empereur-Mot, C., Elad, N. & Levy, E. D. Proteins evolve on the edge of supramolecular self-assembly. *Nature* **548**, 244-247 (2017).

9 Levin, T. *et al.* Profiling the physiological impact of aberrant folded-state protein filamentation in cells. *Mol. Syst. Biol.* **21**,1306–1324 (2025).

10 Zhou, B. *et al.* A conditional protein diffusion model generates artificial programmable endonuclease sequences with enhanced activity. *Cell Discov.* **10**, 95 (2024).

11 Tan, Y., Zhou, B., Zheng, L., Fan, G. & Hong, L. Semantical and Geometrical Protein Encoding Toward Enhanced Bioactivity and Thermostability. *eLife* **13**, RP98033 (2025).

12 Baquero, D. P. *et al.* Extracellular cytochrome nanowires appear to be ubiquitous in prokaryotes. *Cell* **186**, 2853-2864.e2858 (2023).

13 Gu, Y. *et al.* Structure of Geobacter pili reveals secretory rather than nanowire behaviour. *Nature* **597**, 430-434 (2021).

14 Srinivasan, R., Mishra, M., Murata-Hori, M. & Balasubramanian, M. K. Filament Formation of the Escherichia coli Actin-Related Protein, MreB, in Fission Yeast. *Curr. Biol.* **17**, 266-272 (2007).

15 Kruse, T., Bork-Jensen, J. & Gerdes, K. The morphogenetic MreBCD proteins of Escherichia coli form an essential membrane-bound complex. *Mol. Microbiol.* **55**, 78-89 (2005).

16 Dempwolff, F., Reimold, C., Reth, M. & Graumann, P. L. Bacillus subtilis MreB Orthologs Self-Organize into Filamentous Structures underneath the Cell Membrane in a Heterologous Cell System. *PLOS ONE* **6**, e27035 (2011).

17 Altschul, S. F. *et al.* Gapped BLAST and PSI-BLAST: a new generation of protein database search programs. *Nucleic Acids Res.* **25**, 3389-3402 (1997).

18 Shilton, B. H., Shuman, H. A. & Mowbray, S. L. Crystal Structures and Solution Conformations of a Dominant-negative Mutant ofEscherichia coliMaltose-binding Protein. *J. Mol. Biol.* **264**, 364-376 (1996).

19 Gething, M.-J. & Sambrook, J. Protein folding in the cell. *Nature* **355**, 33-45 (1992).

20 Saragovi, A. *et al.* Analysis of cellular water content in T cells reveals a switch from slow metabolic water gain to rapid water influx prior to cell division. *J. Biol. Chem.* **298**, 101795 (2022).
21 Satorras, V. c. G., Hoogeboom, E. & Welling, M. E(n) Equivariant Graph Neural Networks. *PMLR* **139**, 9323-9332 (2021).
22 Lin, Z. *et al.* Evolutionary-scale prediction of atomic-level protein structure with a language model. *Science* **379**, 1123-1130 (2023).
23 Zhou, B. *et al.* Protein Engineering with Lightweight Graph Denoising Neural Networks. *J. Chem. Inf. Model.* **64**, 3650-3661 (2024).
24 Ingraham, J., Garg, V., Barzilay, R. & Jaakkola, T. Generative models for graph-based protein design. *Adv. Neural Inf. Process. Syst.* **32** (2019).
25 Jumper, J. *et al.* Highly accurate protein structure prediction with AlphaFold. *Nature* **596**, 583-589 (2021).
26 Rabadan, M. A. *et al.* An in vitro model of neuronal ensembles. *Nat. Commun.* **13**, 3340 (2022).
27 Mattson, M. P., Rychlik, B., Chu, C. & Christakos, S. Evidence for calcium-reducing and excito-protective roles for the calcium-binding protein calbindin-D28k in cultured hippocampal neurons. *Neuron* **6**, 41-51 (1991).
28 Kaech, S. & Banker, G. Culturing hippocampal neurons. *Nat. Protoc.* **1**, 2406-2415 (2006).
29 Molnár, E. Long-term potentiation in cultured hippocampal neurons. *Semin. Cell Dev. Biol.* **22**, 506-513 (2011).
30 Lin, Y.-C. *et al.* Genome dynamics of the human embryonic kidney 293 lineage in response to cell biology manipulations. *Nat. Commun.* **5**, 4767 (2014).
31 Zhang, J. *et al.* Sall4 modulates embryonic stem cell pluripotency and early embryonic development by the transcriptional regulation of Pou5f1. *Nat. Cell Biol.* **8**, 1114-1123 (2006).
32 Mo, J.-S. *et al.* Cellular energy stress induces AMPK-mediated regulation of YAP and the Hippo pathway. *Nat. Cell Biol.* **17**, 500-510 (2015).
33 Brady, O. A. *et al.* The transcription factors TFE3 and TFEB amplify p53 dependent transcriptional programs in response to DNA damage. *eLife* **7**, e40856 (2018).
34 Dussmann, P. *et al.* Live in vivo imaging of Egr-1 promoter activity during neonatal development, liver regeneration and wound healing. *BMC Dev. Biol.* **11**, 28 (2011).
35 Ortner, V., Ludwig, A., Riegel, E., Dunzinger, S. & Czerny, T. An artificial HSE promoter for efficient and selective detection of heat shock pathway activity. *Cell Stress Chaperones* **20**, 277-288 (2015).
36 Saha, R. N. *et al.* Rapid activity-induced transcription of Arc and other IEGs relies on poised RNA polymerase II. *Nat. Neurosci.* **14**, 848-856 (2011).
37 Kawashima, T., Okuno, H. & Bito, H. A new era for functional labeling of neurons: activity-dependent promoters have come of age. *Front. Neural Circuits* **8**, 37 (2014).
38 Guzowski, J. F., McNaughton, B. L., Barnes, C. A. & Worley, P. F. Environment-specific expression of the immediate-early gene Arc in hippocampal neuronal ensembles. *Nat. Neurosci.* **2**, 1120-1124 (1999).

39 Ebisuya, M., Yamamoto, T., Nakajima, M. & Nishida, E. Ripples from neighbouring transcription. *Nat. Cell Biol.* **10**, 1106-1113 (2008).
40 Cohen-Armon, M., Yeheskel, A. & Pascal, J. M. Signal-induced PARP1-Erk synergism mediates IEG expression. *Signal Transduct. Target. Ther.* **4**, 8 (2019).
41 Roy, D. S. *et al.* Memory retrieval by activating engram cells in mouse models of early Alzheimer's disease. *Nature* **531**, 508-512 (2016).
42 Kawashima, T. *et al.* Synaptic activity-responsive element in the Arc/Arg3.1 promoter essential for synapse-to-nucleus signaling in activated neurons. *Proc. Natl. Acad. Sci.* **106**, 316-321 (2009).
43 Sun, X. *et al.* Functionally Distinct Neuronal Ensembles within the Memory Engram. *Cell* **181**, 410-423.e417 (2020).
44 Brito, D. V. C. *et al.* Biphasic Npas4 expression promotes inhibitory plasticity and suppression of fear memory consolidation in mice. *Mol. Psychiatry* **29**, 1929-1940 (2024).
45 Wu, Y., von Hauff, I. V., Jensen, N., Rossner, M. J. & Wehr, M. C. Improved Split TEV GPCR β-arrestin-2 Recruitment Assays via Systematic Analysis of Signal Peptide and β-arrestin Binding Motif Variants. *Biosensors (Basel)* **13**, 48 (2022).
46 Dash, P. K., Karl, K. A., Colicos, M. A., Prywes, R. & Kandel, E. R. cAMP response element-binding protein is activated by Ca2+/calmodulin- as well as cAMP-dependent protein kinase. *Proc. Natl. Acad. Sci.* **88**, 5061-5065 (1991).
47 Wang, H., Xu, J., Lazarovici, P., Quirion, R. & Zheng, W. cAMP Response Element-Binding Protein (CREB): A Possible Signaling Molecule Link in the Pathophysiology of Schizophrenia. *Front. Mol. Neurosci.* **11**, 255 (2018).
48 Zhang, X. *et al.* Genome-wide analysis of cAMP-response element binding protein occupancy, phosphorylation, and target gene activation in human tissues. *Proc. Natl. Acad. Sci.* **102**, 4459-4464 (2005).
49 Bourtchuladze, R. *et al.* Deficient long-term memory in mice with a targeted mutation of the cAMP-responsive element-binding protein. *Cell* **79**, 59-68 (1994).
50 Malik, A. N. *et al.* Genome-wide identification and characterization of functional neuronal activity–dependent enhancers. *Nat. Neurosci.* **17**, 1330-1339 (2014).
51 Linghu, C. *et al.* Spatial Multiplexing of Fluorescent Reporters for Imaging Signaling Network Dynamics. *Cell* **183**, 1682-1698.e1624 (2020).
52 Nambu, M. F., Lin, Y.-J., Reuschenbach, J. & Tanaka, K. Z. What does engram encode?: Heterogeneous memory engrams for different aspects of experience. *Curr. Opin. Neurobiol.* **75**, 102568 (2022).
53 Gui, Y. *et al.* c-Fos regulated by TMPO/ERK axis promotes 5-FU resistance via inducing NANOG transcription in colon cancer. *Cell Death Dis.* **15**, 61 (2024).
54 Monje, P., Hernández-Losa, J., Lyons, R. J., Castellone, M. D. & Gutkind, J. S. Regulation of the transcriptional activity of c-Fos by ERK. A novel role for the prolyl isomerase PIN1. *J. Biol. Chem.* **280**, 35081-35084 (2005).
55 Richter, K., Haslbeck, M. & Buchner, J. The heat shock response: life on the verge of death. *Mol. Cell* **40**, 253-266 (2010).
56 Maxwell, B. A. *et al.* Ubiquitination is essential for recovery of cellular activities after heat shock. *Science* **372**, eabc3593 (2021).
57 Arai, M., Osanai, H., Snell, C. C., Kitamura, T. & Ogawa, S. K. Combinative protein expression of immediate early genes c-Fos, Arc, and Npas4 along

aversive-and reward-related neural networks. *bioRxiv*, 2025.2004. 2021.649441 (2025).
58 Lalanne, J.-B. *et al.* Multiplex profiling of developmental cis-regulatory elements with quantitative single-cell expression reporters. *Nat. Methods* **21**, 983-993 (2024).
59 Wen, X. *et al.* Single-cell multiplex chromatin and RNA interactions in ageing human brain. *Nature* **628**, 648-656 (2024).
60 Lissek, T. *et al.* Npas4 regulates medium spiny neuron physiology and gates cocaine-induced hyperlocomotion. *EMBO Rep.* **22**, e51882 (2021).
61 Shaywitz, A. J. & Greenberg, M. E. CREB: A Stimulus-Induced Transcription Factor Activated by A Diverse Array of Extracellular Signals. *Annu. Rev. Biochem.* **68**, 821-861 (1999).
62 Miyashita, T., Kikuchi, E., Horiuchi, J. & Saitoe, M. Long-Term Memory Engram Cells Are Established by c-Fos/CREB Transcriptional Cycling. *Cell Rep.* **25**, 2716-2728.e2713 (2018).
63 Ichiki, T. *et al.* Cyclic AMP response element-binding protein mediates reactive oxygen species-induced c-fos expression. *Hypertension* **42**, 177-183 (2003).
64 Sinha, S. *et al.* Behavior-related gene regulatory networks: A new level of organization in the brain. *Proc. Natl. Acad. Sci.* **117**, 23270-23279 (2020).
65 Lepeu, G. *et al.* The critical dynamics of hippocampal seizures. *Nat. Commun.* **15**, 6945 (2024).
66 Lenstra, T. L., Rodriguez, J., Chen, H. & Larson, D. R. Transcription Dynamics in Living Cells. *Annu. Rev. Biophys.* **45**, 25-47 (2016).
67 Li, L., Carter, J., Gao, X., Whitehead, J. & Tourtellotte, W. G. The neuroplasticity-associated arc gene is a direct transcriptional target of early growth response (Egr) transcription factors. *Mol. Cell. Biol.* **25**, 10286-10300 (2005).
68 Penke, Z., Chagneau, C. & Laroche, S. Contribution of Egr1/zif268 to Activity-Dependent Arc/Arg3.1 Transcription in the Dentate Gyrus and Area CA1 of the Hippocampus. *Front. Behav. Neurosci.* **5**, 48 (2011).
69 Gonzales, B. J., Mukherjee, D., Ashwal-Fluss, R., Loewenstein, Y. & Citri, A. Subregion-specific rules govern the distribution of neuronal immediate-early gene induction. *Proc. Natl. Acad. Sci.* **117**, 23304-23310 (2020).
70 Evans, H. T. *et al.* Mapping the spatiotemporal dynamics of de novo protein synthesis during long-term memory formation. *bioRxiv*, 2025.2004.2017.649250 (2025).
71 Gallo, F. T., Katche, C., Morici, J. F., Medina, J. H. & Weisstaub, N. V. Immediate Early Genes, Memory and Psychiatric Disorders: Focus on c-Fos, Egr1 and Arc. *Front. Behav. Neurosci.* **12**, 79 (2018).
72 Gossen, M. *et al.* Transcriptional Activation by Tetracyclines in Mammalian Cells. *Science* **268**, 1766-1769 (1995).
73 Zhou, X., Vink, M., Klaver, B., Berkhout, B. & Das, A. T. Optimization of the Tet-On system for regulated gene expression through viral evolution. *Gene Ther.* **13**, 1382-1390 (2006).
74 Ma, J.-H. *et al.* Type I interferon signaling enhances kainic acid-induced seizure severity. *bioRxiv*, 2024.2011.2013.623521 (2024).

75 Joo, J.-Y., Schaukowitch, K., Farbiak, L., Kilaru, G. & Kim, T.-K. Stimulus-specific combinatorial functionality of neuronal c-fos enhancers. *Nat. Neurosci.* **19**, 75-83 (2016).
76 Dyrvig, M. *et al.* Epigenetic regulation of Arc and c-Fos in the hippocampus after acute electroconvulsive stimulation in the rat. *Brain Res. Bull.* **88**, 507-513 (2012).
77 Choi, J.-H. *et al.* Interregional synaptic maps among engram cells underlie memory formation. *Science* **360**, 430-435 (2018).
78 Liu, X. *et al.* Optogenetic stimulation of a hippocampal engram activates fear memory recall. *Nature* **484**, 381-385 (2012).
79 Wiedenmann, J., Oswald, F. & Nienhaus, G. U. Fluorescent proteins for live cell imaging: Opportunities, limitations, and challenges. *IUBMB Life* **61**, 1029-1042 (2009).
80 Day, R. N. & Davidson, M. W. The fluorescent protein palette: tools for cellular imaging. *Chem. Soc. Rev.* **38**, 2887-2921 (2009).
81 Wu, P.-H. *et al.* A comparison of methods to assess cell mechanical properties. *Nat. Methods* **15**, 491-498 (2018).
82 Shaner, N. C., Steinbach, P. A. & Tsien, R. Y. A guide to choosing fluorescent proteins. *Nat. Methods* **2**, 905-909 (2005).
83 Beagan, J. A. *et al.* Three-dimensional genome restructuring across timescales of activity-induced neuronal gene expression. *Nat. Neurosci.* **23**, 707-717 (2020).
84 Minatohara, K., Akiyoshi, M. & Okuno, H. Role of Immediate-Early Genes in Synaptic Plasticity and Neuronal Ensembles Underlying the Memory Trace. *Front. Mol. Neurosci.* **8**, 78 (2015).
85 Wen, A. Y., Sakamoto, K. M. & Miller, L. S. The role of the transcription factor CREB in immune function. *J. Immunol.* **185**, 6413-6419 (2010).
86 Eckerling, A., Ricon-Becker, I., Sorski, L., Sandbank, E. & Ben-Eliyahu, S. Stress and cancer: mechanisms, significance and future directions. *Nat. Rev. Cancer* **21**, 767-785 (2021).
87 Liu, C. *et al.* Spatiotemporal mapping of gene expression landscapes and developmental trajectories during zebrafish embryogenesis. *Dev. Cell* **57**, 1284-1298.e1285 (2022).
88 Briggs, J. A. *et al.* The dynamics of gene expression in vertebrate embryogenesis at single-cell resolution. *Science* **360**, eaar5780 (2018).
89 Schmidt, F., Cherepkova, M. Y. & Platt, R. J. Transcriptional recording by CRISPR spacer acquisition from RNA. *Nature* **562**, 380-385 (2018).
90 Bhattarai-Kline, S. *et al.* Recording gene expression order in DNA by CRISPR addition of retron barcodes. *Nature* **608**, 217-225 (2022).
91 Hao, K. *et al.* Reconstructing signaling history of single cells with imaging-based molecular recording. *bioRxiv*, 2024.2010.2011.617908 (2024).
92 Zhang, Y. *et al.* Fast and sensitive GCaMP calcium indicators for imaging neural populations. *Nature* **615**, 884-891 (2023).
93 Sakaue-Sawano, A. *et al.* Visualizing Spatiotemporal Dynamics of Multicellular Cell-Cycle Progression. *Cell* **132**, 487-498 (2008).
94 Wang, W. *et al.* A light- and calcium-gated transcription factor for imaging and manipulating activated neurons. *Nat. Biotechnol.* **35**, 864-871 (2017).

95 Lee, D., Hyun, J. H., Jung, K., Hannan, P. & Kwon, H.-B. A calcium- and light-gated switch to induce gene expression in activated neurons. *Nat. Biotechnol.* **35**, 858-863 (2017).
96 Regot, S., Hughey, Jacob J., Bajar, Bryce T., Carrasco, S. & Covert, Markus W. High-Sensitivity Measurements of Multiple Kinase Activities in Live Single Cells. *Cell* **157**, 1724-1734 (2014).
97 Chan, K. Y. *et al.* Engineered AAVs for efficient noninvasive gene delivery to the central and peripheral nervous systems. *Nat. Neurosci.* **20**, 1172-1179 (2017).
98 Wroblewska, A. *et al.* Protein Barcodes Enable High-Dimensional Single-Cell CRISPR Screens. *Cell* **175**, 1141-1155.e1116 (2018).
99 Vanderstraeten, J. & Briers, Y. Synthetic protein scaffolds for the colocalisation of co-acting enzymes. *Biotechnol. Adv.* **44**, 107627 (2020).
100 Ryu, J. & Park, S. H. Simple synthetic protein scaffolds can create adjustable artificial MAPK circuits in yeast and mammalian cells. *Sci. Signal* **8**, ra66 (2015).
101 Chen, Z. *et al.* A synthetic protein-level neural network in mammalian cells. *Science* **386**, 1243-1250 (2024).
102 Kapust, R. B. & Waugh, D. S. Escherichia coli maltose-binding protein is uncommonly effective at promoting the solubility of polypeptides to which it is fused. *Protein Sci.* **8**, 1668-1674 (1999).
103 Frei, M. S. *et al.* Engineered HaloTag variants for fluorescence lifetime multiplexing. *Nat. Methods* **19**, 65-70 (2022).
104 Sørensen, A. T. *et al.* A robust activity marking system for exploring active neuronal ensembles. *eLife* **5**, e13918 (2016).
