# Supplementary material for: Scalable and multiplexed recorders of gene regulation dynamics across weeks
Source: Nature. 2026 Jan 26;652(8111):1038–48. doi: 10.1038/s41586-026-10156-9 (PMC13102694; doi:10.1038/s41586-026-10156-9)
Supplement: Supplementary file 2 — Reporting Summary [file 41586_2026_10156_MOESM2_ESM.pdf]

nature portfolio

Corresponding author(s): Changyang Linghu

Last updated by author(s): Jan 4, 2026

Reporting Summary

Nature Portfolio wishes to improve the reproducibility of the work that we publish. This form provides structure for consistency and transparency in reporting. For further information on Nature Portfolio policies, see our Editorial Policies and the Editorial Policy Checklist.

Statistics

For all statistical analyses, confirm that the following items are present in the figure legend, table legend, main text, or Methods section.

n/a	Confirmed	
☐	☒	The exact sample size (*n*) for each experimental group/condition, given as a discrete number and unit of measurement
☐	☒	A statement on whether measurements were taken from distinct samples or whether the same sample was measured repeatedly
☐	☒	The statistical test(s) used AND whether they are one- or two-sided<br>*Only common tests should be described solely by name; describe more complex techniques in the Methods section.*
☒	☐	A description of all covariates tested
☐	☒	A description of any assumptions or corrections, such as tests of normality and adjustment for multiple comparisons
☐	☒	A full description of the statistical parameters including central tendency (e.g. means) or other basic estimates (e.g. regression coefficient) AND variation (e.g. standard deviation) or associated estimates of uncertainty (e.g. confidence intervals)
☐	☒	For null hypothesis testing, the test statistic (e.g. *F*, *t*, *r*) with confidence intervals, effect sizes, degrees of freedom and *P* value noted<br>*Give P values as exact values whenever suitable.*
☒	☐	For Bayesian analysis, information on the choice of priors and Markov chain Monte Carlo settings
☐	☒	For hierarchical and complex designs, identification of the appropriate level for tests and full reporting of outcomes
☒	☐	Estimates of effect sizes (e.g. Cohen's *d*, Pearson's *r*), indicating how they were calculated

Our web collection on statistics for biologists contains articles on many of the points above.

Software and code

Policy information about availability of computer code

Data collection	Nikon NIS-Elements software was used for image acquisition on fluorescence microscopes.
Data analysis	Statistical analysis was performed in Prism 10 (GraphPad). Image analysis was performed in ImageJ (National Institutes of Health), napari (napari contributors; doi:10.5281/zenodo.3555620), and Python. The code developed in this work for in vivo image analysis, Tape Reader v1.0, can be found at https://github.com/LinghuLab/TapeReader. The code developed in this work for signal extraction and analysis of protein tape recordings, Tape Analyzer v1.0, can be found at https://github.com/LinghuLab/TapeAnalyzer. The structure of the protein monomer for simulations was predicted by previously published AlphaFold3 (alphafoldserver.com). Molecular dynamics simulations were performed using previously published GROMACS 2021.1 packages (manual.gromacs.org/2021.1). The previously published ProtSSN model for protein mutation prediction can be found at https://github.com/tyang816/ProtSSN. The previously published CPDiffusion model for protein sequence generation prediction can be found at https://github.com/bzho3923/CPDiffusion. Video analysis of mouse behavior was performed by ezTrack v1.2 (https://github.com/denisecailab/ezTrack) and LabGym v2.9.0 (https://github.com/umyelab/LabGym).

For manuscripts utilizing custom algorithms or software that are central to the research but not yet described in published literature, software must be made available to editors and reviewers. We strongly encourage code deposition in a community repository (e.g. GitHub). See the Nature Portfolio guidelines for submitting code & software for further information.

Data

Policy information about availability of data

All manuscripts must include a data availability statement. This statement should provide the following information, where applicable:

- Accession codes, unique identifiers, or web links for publicly available datasets
- A description of any restrictions on data availability
- For clinical datasets or third party data, please ensure that the statement adheres to our policy

The plasmids and the corresponding sequence of working CytoTape constructs reported in this paper are available at Addgene (plasmid IDs 239423-239430, 239616, and 250670-250672). The mouse brain in vivo recording datasets generated and analyzed in this study are available at Zenodo (https://doi.org/10.5281/zenodo.18123891). Supplementary Information includes the development and discussion of the CytoTape toolkit, Supplementary Figs. 1-24, Supplementary Video 1, Supplementary Tables 1-11, and Supplementary References. There is no restriction on data availability.

Research involving human participants, their data, or biological material

Policy information about studies with human participants or human data. See also policy information about sex, gender (identity/presentation), and sexual orientation and race, ethnicity and racism.

Reporting on sex and gender	N/A
Reporting on race, ethnicity, or other socially relevant groupings	N/A
Population characteristics	N/A
Recruitment	N/A
Ethics oversight	N/A

Note that full information on the approval of the study protocol must also be provided in the manuscript.

Field-specific reporting

Please select the one below that is the best fit for your research. If you are not sure, read the appropriate sections before making your selection.

[x] Life sciences [ ] Behavioural & social sciences [ ] Ecological, evolutionary & environmental sciences

For a reference copy of the document with all sections, see nature.com/documents/nr-reporting-summary-flat.pdf

Life sciences study design

All studies must disclose on these points even when the disclosure is negative.

Sample size	For cell culture experiments and mouse experiments (excluding behavioral tests), sample sizes were chosen based on our previously published molecular technology development work (Nature Biotechnology 41, 640–651, 2023; Cell 183, 1682-1698, 2020) and established literature in molecular biosensor and recorder development. For mouse behavioral tests, sample sizes were determined based on the expected variance and effect sizes reported in relevant previous studies (Nature 531, 508-512, 2016; Cell 181, 410-423, 2020). No statistical method was used to predetermine sample sizes. This approach is consistent with the primary objective of this work, which is to develop, validate, and demonstrate a novel molecular technology. We found the sample sizes sufficient to yield reproducible results.
Data exclusions	We applied multiple quality control criteria to ensure the computationally segmented fiber structures from mouse brain tissue accurately reflected fiber morphology and anatomical positioning. First, segmented structures with length smaller than 8 μm were excluded. Next, to ensure morphological accuracy of segmentation, segmented structures with non-fiber-like morphology were removed through principal component analysis (PCA). Specifically, we calculated the explained variance of the first principal component of the skeleton of the segmented structure and excluded segmented structures with values below 0.8. Finally, fibers located outside soma regions detected by Nissl staining were excluded from further analysis. In 14-day and 18-day mouse brain in vivo recording experiments, if there is more than one fiber in a cell, the longest fiber was used for subsequent analysis. For electrophysiological characterization of synaptic transmission, recordings were excluded if the series resistance exceeded 20 MΩ to ensure high-quality voltage clamp.
Replication	Experiments were replicated at least once. All attempts at replication were successful. The detailed experimental protocols are provided to facilitate replication by others.
Randomization	All biological replicates were treated identically, and randomization was not relevant to this technology development work.
Blinding	Samples for all biological replicates were obtained under identical conditions. Experimenters were blinded to manipulation when analyzing the data, except for mouse behavior tests where experimenters were not blinded to group identity.

Reporting for specific materials, systems and methods

We require information from authors about some types of materials, experimental systems and methods used in many studies. Here, indicate whether each material, system or method listed is relevant to your study. If you are not sure if a list item applies to your research, read the appropriate section before selecting a response.

Materials & experimental systems

n/a	Involved in the study
☐	☒ Antibodies
☐	☒ Eukaryotic cell lines
☒	☐ Palaeontology and archaeology
☐	☒ Animals and other organisms
☒	☐ Clinical data
☒	☐ Dual use research of concern
☒	☐ Plants

Methods

n/a	Involved in the study
☒	☐ ChIP-seq
☒	☐ Flow cytometry
☒	☐ MRI-based neuroimaging

Antibodies

Antibodies used

Primary antibodies (1:500 for immunofluorescence of cultured cells and brain slices):
HA Tag Monoclonal Antibody (clone name: C29F4), Rabbit IgG, Cell Signaling Technology Cat# 3724; RRID: AB_1549585
HA Tag Polyclonal Antibody, Chicken IgY, Invitrogen Cat# PA5-33243; RRID: AB_2550658
V5 Tag Monoclonal Antibody (clone name: SV5-Pk1), Mouse IgG2a, Invitrogen Cat# R960-25; RRID: AB_2556564
DYKDDDDK (FLAG) Tag Superclonal Antibody (clone names: 20H18L16, 20H1L23, 8H2L5, 8H8L17), Rabbit IgG, Invitrogen Cat# 740001;RRID: AB_2610628
OLLAS Tag Monoclonal Antibody (clone name: L2), Rat IgG1 kappa, Invitrogen Cat# MA5-16125; RRID: AB_11152481
E Tag Monoclonal Antibody (clone name: 11H12B3), Mouse IgG1, Invitrogen Cat# MA5-38276; RRID: AB_2898191
c-Fos Monoclonal Antibody (clone name: Ch108B5), Chicken IgY, Synaptic Systems Cat# 226 009; RRID: AB_2943525
Phospho-CREB (Ser133) Monoclonal Antibody (clone name: 87G3), Rabbit IgG, Cell Signaling Technology Cat# 9198; RRID: AB_2561044
Phospho-Elk-1 (Ser383) Monoclonal Antibody (clone name: 2B1), Mouse IgG1, Cell Signaling Technology Cat# 9186; RRID: AB_2277933
Egr1 Monoclonal Antibody (clone name: 15F7), Rabbit IgG, Cell Signaling Technology Cat# 4153; RRID: AB_2097038
Phospho-p44/42 MAPK (Erk1/2) (Thr202/Tyr204) Monoclonal Antibody (clone name: D13.14.4E), Rabbit IgG, Cell Signaling Technology Cat# 4370; RRID: AB_2315112
GFAP Monoclonal Antibody (clone name: D1F4Q), Rabbit IgG, Cell Signaling Technology Cat# 12389; RRID: AB_2631098
Cleaved Caspase-3 (Asp175) Monoclonal Antibody (clone name: 5A1E), Rabbit IgG, Cell Signaling Technology Cat# 9664; RRID: AB_2070042
HSP70 Polyclonal Antibody, Rabbit IgG, Cell Signaling Technology Cat# 4872; RRID: AB_2279841
HSP27 Monoclonal Antibody (clone name: G31), Mouse IgG1, Cell Signaling Technology Cat# 2402; RRID: AB_331761
Phospho-Histone H2A.X (γH2AX; Ser139) Antibody (clone name: JBW301), Mouse IgG1, Sigma Cat# 05-636; RRID: AB_309864
Synaptophysin Monoclonal Antibody (clone name: SVP-38), Mouse IgG1, Sigma Cat# S5768; RRID: AB_477523
NeuN Polyclonal Antibody, Guinea Pig IgG, Synaptic Systems Cat# 266 004; RRID: AB_2619988
Iba1 Polyclonal Antibody, Rabbit IgG, Wako Chemicals Cat# 019-19741; RRID: AB_839504
Ki67 Monoclonal Antibody (clone name: B56), Mouse IgG1, Abcam Cat# ab279653; RRID: AB_2934265
GRP78 BiP Polyclonal Antibody, Rabbit IgG, Abcam Cat# ab21685; RRID: AB_2119834
TOMM20 Monoclonal Antibody (clone name: EPR15581-54), Rabbit IgG, Abcam Cat# ab186735; RRID: AB_2889972

Fluorescent secondary antibodies (1:500 for immunofluorescence of cultured cells and brain slices):
Goat anti-Mouse IgG1 (Gamma 1 chain) Pre-absorbed Secondary Antibody, ATTO 425, Rockland Cat# 610-151-040; RRID: AB_2614850
Goat anti-Mouse IgG2a Cross-Adsorbed Secondary Antibody, Alexa Fluor 488, Invitrogen Cat# A-21131; RRID: AB_2535771
Goat anti-Guinea Pig IgG (H+L) Highly Cross-Adsorbed Secondary Antibody, Alexa Fluor 488, Invitrogen Cat# A-11073; RRID: AB_2534117
Goat anti-Rabbit IgG (H+L) Cross-Adsorbed Secondary Antibody, Alexa Fluor 488, Invitrogen Cat# A-11008; RRID: AB_143165
Goat anti-Rabbit IgG (H+L) Highly Cross-Adsorbed Secondary Antibody, Alexa Fluor Plus 488, Invitrogen Cat# A-32731; RRID: AB_2633280
Goat anti-Mouse IgG2a Cross-Adsorbed Secondary Antibody, Alexa Fluor 546, Invitrogen Cat# A-21133; RRID: AB_2535772
Goat anti-Rat IgG (H+L) Cross-Adsorbed Secondary Antibody, Alexa Fluor 546, Invitrogen Cat# A-11081; RRID: AB_2534125
Goat anti-Rabbit IgG (H+L) Cross-Adsorbed Secondary Antibody, Alexa Fluor 594, Invitrogen Cat# A-11012; RRID: AB_2534079
Goat anti-Chicken IgY (H+L) Cross-Adsorbed Secondary Antibody, Alexa Fluor Plus 647, Invitrogen Cat# A-32933; RRID: AB_2762845
Goat anti-Mouse IgG1 Cross-Adsorbed Secondary Antibody, Alexa Fluor 647, Invitrogen Cat# A-21240; RRID: AB_2535809

Additional details of primary antibodies, secondary antibodies, dyes, and other reagents used in this study are listed in Supplementary Table S10.

Validation

Validation statements for use in immunohistochemistry and relevant citations of the primary antibodies used in this study are listed on the manufacturers' website listed below. Citations of the antibodies can also be searched at www.citeab.com

anti-Etag (Invitrogen, Cat# MA5-38276), https://www.thermofisher.com/antibody/product/E-Tag-Antibody-clone-11H12B3-Monoclonal/MA5-38276
anti-V5 (Invitrogen, Cat# R960-25), https://www.thermofisher.com/antibody/product/V5-Tag-Antibody-clone-SV5-Pk1-Monoclonal/R960-25
anti-OLLAS (Invitrogen, Cat# MA5-16125), https://www.thermofisher.com/antibody/product/OLLAS-Tag-Antibody-clone-L2-Monoclonal/MA5-16125
anti-FLAG (Invitrogen, Cat# 740001), https://www.thermofisher.com/antibody/product/DYKDDDDK-Tag-Antibody-clone-20H18L16-20H1L23-8H2L5-8H8L17-Recombinant-Superclonal/740001
anti-HA (Cell Signaling Technology, Cat# 3724), https://www.cellsignal.com/products/primary-antibodies/ha-tag-c29f4-rabbit-monoclonal-antibody/3724
anti-HA (Invitrogen, Cat# PA5-33243), https://www.thermofisher.com/antibody/product/HA-Tag-Antibody-Polyclonal/PA5-33243
anti-c-Fos (Synaptic Systems, Cat# 226009), https://sysy.com/product/226009
anti-Phospho-CREB (Ser133) (Cell Signaling Technology, Cat# 9198), https://www.cellsignal.com/products/primary-antibodies/phospho-creb-ser133-87g3-rabbit-mab/9198
anti-GFAP (Cell Signaling Technology, Cat# 12389), https://www.cellsignal.com/products/primary-antibodies/gfap-d1f4q-xp-rabbit-mab/12389
anti-Cleaved Caspase-3 (Cell Signaling Technology, Cat# 9664), https://www.cellsignal.com/products/primary-antibodies/cleaved-caspase-3-asp175-5a1e-rabbit-mab/9664
anti-Hsp70 (Cell Signaling Technology, Cat# 4872), https://www.cellsignal.com/products/primary-antibodies/hsp70-antibody/4872
anti-Hsp27 (Cell Signaling Technology, Cat# 2402), https://www.cellsignal.com/products/primary-antibodies/hsp27-g31-mouse-mab/2402
anti-γH2AX (Millipore, Cat# 05-636), https://www.sigmaaldrich.com/US/en/product/mm/05636
anti-Synaptophysin (Sigma, Cat# S5768), https://www.sigmaaldrich.com/US/en/product/sigma/s5768
anti-NeuN (SYSY, Cat# 266004), https://sysy.com/product/266004
anti-Iba1 (Wako Chemicals, Cat# ab279653), https://fujifilmbiosciences.fujifilm.com/us/anti-iba1-goat.html
anti-Ki67 (Abcam, Cat# ab279653), https://www.abcam.com/en-us/products/primary-antibodies/ki67-antibody-b56-ab279653
anti-GRP78 BiP (Abcam, Cat# ab21685), https://www.abcam.com/en-us/products/primary-antibodies/grp78-bip-antibody-ab21685
anti-TOMM20 (Abcam, Cat# ab186735), https://www.abcam.com/en-us/products/primary-antibodies/tomm20-antibody-epr15581-54-mitochondrial-marker-ab186735
anti-Phospho-Elk-1 (Cell Signaling Technology, Cat# 9186), https://www.cellsignal.com/products/primary-antibodies/phospho-elk-1-ser383-2b1-mouse-mab/9186
anti-Egr1(Cell Signaling Technology, Cat# 4153), https://www.cellsignal.com/products/primary-antibodies/egr1-15f7-rabbit-mab/4153
anti-Phospho-p44/42 MAPK (Erk1/2) (Thr202/Tyr204) (D13.14.4E) (Cell Signaling Technology, Cat# 4370), https://www.cellsignal.com/products/primary-antibodies/phospho-p44-42-mapk-erk1-2-thr202-tyr204-d13-14-4e-xp-rabbit-mab/4370

Eukaryotic cell lines

Policy information about cell lines and Sex and Gender in Research

Cell line source(s)	HEK293T clone 17 (CRL-11268) and HeLa (CCL-2) cell lines from ATCC.
Authentication	The cell line was authenticated by the manufacturer via STR profiling.
Mycoplasma contamination	The cell lines were tested for mycoplasma contamination by the manufacturer to their standard levels of stringency (mycoplasma contamination was not detected).
Commonly misidentified lines (See ICLAC register)	No commonly misidentified cell lines were used in the study.

Animals and other research organisms

Policy information about studies involving animals; ARRIVE guidelines recommended for reporting animal research, and Sex and Gender in Research

Laboratory animals	Male and female Swiss Webster mice at postnatal day 0 or 1 (Taconic), male C57BL/6J mice at 2-5 months of age (Jackson Laboratory). Mice were maintained on a 12-hour light/dark cycle (lights on at 06:00 EST) in a temperature-controlled environment at 22±1°C, with a relative humidity of 30-50%. Mice were group-housed, except for pregnant females (individually housed prior to delivery) and mice post-surgery (individually housed for the remainder of the experiment).
Wild animals	The study did not involve wild animals.
Reporting on sex	Male and female Swiss Webster neonatal mice were used randomly in this study, delivered by pregnant females. Male C57BL/6J mice were used in this study.
Field-collected samples	The study did not involve field-collected samples.
Ethics oversight	All animal procedures were conducted in accordance with the United States National Institutes of Health Guide for the Care and Use of Laboratory Animals and were approved by the Institutional Animal Care and Use Committee of the institution where each procedure was conducted (University of Michigan, Icahn School of Medicine at Mount Sinai, or Massachusetts Institute of Technology).

Note that full information on the approval of the study protocol must also be provided in the manuscript.

Plants

Seed stocks	*Report on the source of all seed stocks or other plant material used. If applicable, state the seed stock centre and catalogue number. If plant specimens were collected from the field, describe the collection location, date and sampling procedures.*
Novel plant genotypes	*Describe the methods by which all novel plant genotypes were produced. This includes those generated by transgenic approaches, gene editing, chemical/radiation-based mutagenesis and hybridization. For transgenic lines, describe the transformation method, the number of independent lines analyzed and the generation upon which experiments were performed. For gene-edited lines, describe the editor used, the endogenous sequence targeted for editing, the targeting guide RNA sequence (if applicable) and how the editor was applied.*
Authentication	*Describe any authentication procedures for each seed stock used or novel genotype generated. Describe any experiments used to assess the effect of a mutation and, where applicable, how potential secondary effects (e.g. second site T-DNA insertions, mosiacism, off-target gene editing) were examined.*
